# Intentional Formation of Persistent Surface Redox Mediators by Adsorption of Polyconjugated Carbonyl Complexes to Pd Nanoparticles

**DOI:** 10.1021/jacs.4c15874

**Published:** 2025-04-04

**Authors:** Jason S. Adams, Mayank Tanwar, Haoyu Chen, Sucharita Vijayaraghavan, Tomas Ricciardulli, Matthew Neurock, David W. Flaherty

**Affiliations:** † Department of Chemical and Biomolecular Engineering, 14589 University of Illinois at Urbana-Champaign, Urbana, Illinois 61801, United States; ‡ Department of Chemical Engineering and Materials Science, 5635 University of Minnesota, Minneapolis, Minnesota 55455, United States; § School of Chemical and Biomolecular Engineering, 1372 Georgia Institute of Technology, Atlanta, Georgia 30332, United States

## Abstract

Adsorbing polyconjugated carbonyl and aromatic species to Pd nanoparticles forms persistent intermediates that mediate reactions between hydrogen and oxygen-derived species. These surface redox mediators form *in situ* and increase selectivities toward H_2_O_2_ formation (∼65–85%) compared to unmodified Pd nanoparticles (∼45%). Infrared spectroscopy, temperature-programmed oxidation measurements, and *ab initio* calculations show that these species adsorb irreversibly to Pd surfaces and persist over extended periods of catalysis. Combined rates and kinetic isotope effect measurements and simulations suggest that carbonyl groups of bound organics react heterolytically with hydrogen to form partially hydrogenated oxygenated complexes. Subsequently, these organic species transfer proton–electron pairs to O_2_-derived surface species via pathways that favor H_2_O_2_ over H_2_O formation on Pd nanoparticles. Computational and experimental measurements show redox pathways mediated by partially hydrogenated carbonyl species form H_2_O_2_ with lower barriers than competing processes while also obstructing O–O bond dissociation during H_2_O formation. For example, adsorption and hydrogenation of hexaketocyclohexane on Pd forms species that react with oxygen with high H_2_O_2_ selectivities (85 ± 8%) for 130 h on stream in flowing water without additional promoters or cosolvents. These paths resemble the anthraquinone auto-oxidation process (AAOP) used for industrial H_2_O_2_ production. These surface-bound species form partially hydrogenated intermediates that mediate H_2_O_2_ formation with high rates and selectivities, comparable to AAOP but on a single catalytic nanoparticle in pure water without organic solvents or multiunit reaction-separation chains. The molecular insights developed herein provide strategies to avoid organic solvents in selective processes and circumvent their associated process costs and environmental impacts.

## Graphical abstract

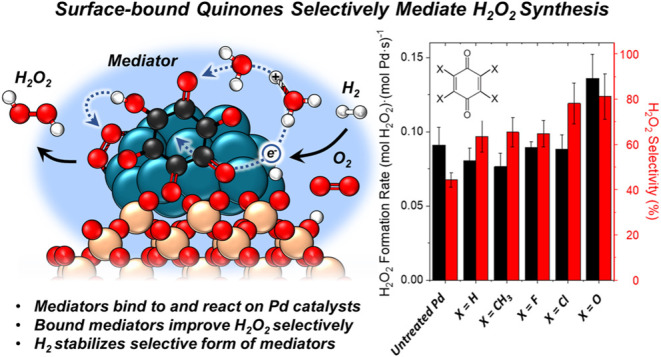

## 1 Introduction

The coordination of organic molecules with metal atoms influences catalytic reactions by altering the electronic and geometric structure of active sites, changing interactions with solvent molecules, and reacting directly with bound species.^1–5^ These phenomena are critical to the design of organometallic centers in homogeneous and enzymatic catalysts.^3,4^ In heterogeneous systems, organic species (e.g., capping agents) can control the size,^6–8^ shape,^9–11^ and composition of colloidal nanoparticles^12,13^ but also block active sites^14–16^ and alter rates and selectivities.^17–19^ For example, phosphine ligands inhibit the over-reduction of product molecules and improve selectivity for the partial hydrogenation of alkynes, carbonyls, and aromatics over late transition metals (e.g., Rh, Ni, Pd).^20–24^ However, it is unclear how the interface of organic species and metal surfaces impacts the active sites and mechanisms involved in converting small molecules at solid–liquid interfaces.^17,19,25–28^

Organic ligands can take on many forms, such as surfactants, ionic liquids, simple small molecules, or coadsorbates.^19,25,28^ These species can transfer charge with catalytic surfaces and reactant molecules and alter adsorption, activation, and desorption of surface intermediates,^25,29–31^ which impacts rates and selectivities for catalytic reactions.^19,25–28^ For example, organic species can bind to metal surfaces, disrupt contiguous ensembles of metal atoms, and block sites that mediate undesired reaction pathways.^32–35^ Such species may introduce bulky organic functions that affect the solvation sphere of catalytic surfaces^36,37^ or sterically hinder the binding of reactive intermediates.^32,35,38,39^ Furthermore, ligands can induce electric fields^40^ and introduce acid–base functionalities^41–46^ that stabilize or react with intermediates. These features offer several tactics to modify the reactivity of solid catalysts, but multiple interactions often coincide and obscure the origins of these effects.

Noble metals (e.g., Pd, Pt) are effective catalysts for hydrogenating oxygen and carbonyl-containing substrates, particularly in the presence of water molecules that aid the oxidation of adsorbed hydrogen atoms to protons and electrons during the reduction of such substrates.^47,48^ Notably, reactions among H_2_ and O_2_ over metal nanoparticles respond sensitively to the presence of organic species within protic solvents. For example, the adsorption of hexadecyl-2-hydroxyethyl-dimethylammonium dihydrogen phosphate (HHDMA) on Pd improves the selectivity to form H_2_O_2_ (∼80%) by increasing barriers of O–O bond dissociation relative to clean Pd surfaces.^32^ Here, the authors hypothesized that HHDMA binds strongly to Pd and induces steric hindrance, stabilizing dioxygen intermediates (e.g., O_2_*, OOH*, H_2_O_2_*). Later work examined a wider range of organic ligands and gave evidence that −OH functional groups present upon adsorbed ligands improved selectivities,^49^ consistent with observations for PVA-capped nanoparticles of Pd.^50,51^ These observations supported the interpretation that associated hydrogen-bonding networks inhibit O–O bond dissociation paths. However, subsequent computation indicated that the −OH functions of H-bonding thiolates (e.g., ethanethiol, β-mercaptoethanol, and thioglycolic acid) bound to metal surfaces preferentially stabilize monatomic oxygen (e.g., O*) over dioxygen (O_2_*) intermediates,^52^ suggesting that hydrogen-bonding networks should favor O–O bond cleavage and subsequent H_2_O formation over H_2_O_2_ formation. Instead, these authors hypothesized that −OH functional groups of adsorbates may present alternative proton-coupled electron transfer (PCET) mechanisms for O–O bond dissociation. Taken together, the reasons for differences in rates and selectivities for reactions among H_2_ and O_2_ caused by the addition of organic surface species remain controversial and may involve site blocking, hydrogen-bonding of surface species, solvation at the surface, and the introduction of additional PCET pathways at solid–liquid interfaces.

Recent findings from our group contributed to a further understanding of these complex systems. We demonstrated that organic solvent molecules (e.g., methanol) spontaneously react with metal surfaces to form surface complexes that mediate reactions among H_2_ and O_2_ on Pd nanoparticles.^53^ A combination of computation, isotopic, and kinetic measurements show that methanol binds to Pd to form hydroxymethyl (e.g., CH_2_OH*) redox mediators that provide low barrier pathways that reduce O_2_-derived intermediates by PCET steps. Moreover, these reactions form formaldehyde (CH_2_O*) that reacts spontaneously with H* to regenerate CH_2_OH* and propagates a cycle of cocatalyzed heterolytic reactions of H_2_ and O_2_ with lower barriers than on clean surfaces of Pd. These findings align with other reports showing that organic species with carbonyl or hydroxyl functions facilitate selective hydrogen transfer reactions over metal nanoparticles by heterolytic processes.^20,41,54–56^ Thus, these carbonyl and hydroxyl intermediates act as surface-bound mediators that cocatalyze hydrogenation reactions analogous to homogeneous outer sphere mediators such as TEMPO in electrocatalytic systems and NADH in enzymatic reactions.^57–59^

The present study builds upon our prior work while drawing inspiration from the anthraquinone auto-oxidation process (AAOP), the dominant method for industrial production of H_2_O_2_.^53,60^ In these systems, the carbonyl functions of quinone molecules react with H_2_ over Pd surfaces, generating partially hydrogenated quinone species that react with O_2_ and form H_2_O_2_ in separate homogeneous steps within organic media. Here, we demonstrate the *intentional* creation of persistent surface redox mediators that increase both rates and selectivities for H_2_O_2_ formation during steady-state catalysis in flowing water. Following *ex situ* adsorption of mediators from the solution, the modified Pd catalysts were tested within a fixed-bed reactor under a continuous flow of the reactants (H_2_ and O_2_) and pure liquid water. Measured rates, kinetic isotope effects, and catalyst stabilities, along with density functional theory (DFT) derived reaction energetics, suggest that PCET steps mediated by quinone-derived surface redox mediators on Pd nanoparticles allow H_2_ and O_2_ to react together through pathways that favor the production of H_2_O_2_. These surface-bound complexes increase H_2_O_2_ selectivities (up to 85%) relative to unmodified Pd (45%). Comparisons between experimentally measured and calculated activation barriers show that electrophilic mediators not only facilitate low-barrier pathways to form H_2_O_2_ but also inhibit O–O bond dissociation steps that yield H_2_O. The organic surface redox mediators persist on Pd surfaces, leading to high rates and selectivities for long reaction periods (>130 h on stream; turnover numbers above 10^6^), consistent with the postreaction characterization of the used catalysts and highly exothermic adsorption energies of the mediators. Consequently, these findings demonstrate the intentional creation of surface redox mediators that cocatalyze reactions of H_2_ and O_2_ in water, inhibit undesired reaction pathways, and eliminate the need for organic solvents for H_2_O_2_ production and related chemistries.

## 2 Experimental Methods

The concise details of the experimental methods are discussed below. More thorough descriptions are provided in the Supporting Experimental Methods section, reporting additional details of experiments, relevant chemicals, purities, and vendors.

### 2.1 Catalyst Preparation

The Pd-SiO_2_ catalysts were synthesized by strong electrostatic adsorption of cationic Pd precursors (Pd­(NO_3_)_2_ or (NH_4_)_2_PdCl_4_) onto a porous SiO_2_ support. Briefly, the SiO_2_ was soaked in a solution of DI H_2_O and NH_4_OH and then mixed with a dissolved solution of the Pd precursor. The resulting mixture was stirred intermittently for 1h and left overnight. The solids were washed with DI water and vacuum-filtered for 24 h. The dried material was heated within a quartz tube furnace to 673 K for 4 h within a mixture of 2:1 He/Air. Then, the sample was purged with He and heated at 573 K for 4 h within a mixture of 4:1 He/H_2_. The sample reached room temperature and was passivated in a mixture of 199:1 He/Air for 1 h before removal from the furnace.

The resulting Pd-SiO_2_ catalyst was treated with organic species (e.g., benzoquinone derivatives), in which 230 mM of the organic were typically dissolved in dioxane, a solvent that negligibly affects rates (*vide infra*; Figure S17). These concentrations saturate the surface of Pd nanoparticles with the organic (*vide infra;* Figure 5). These solutions were sparged with a mixture of 4:1 He/H_2_ for at least 10 min before adding the Pd-SiO_2_, which equilibrated for 1 h under continuously flowing H_2_. The samples were then sealed and soaked for an additional 3 h. The resulting solids were then vacuum-filtered overnight. Some excess organic material was mixed within the catalyst, which was removed by flowing water (35 cm^3^ min^–1^) during catalysis.

### 2.2 Characterization of Catalytic Materials

#### 2.2.1 Pd Nanoparticle Size and Composition

The numerical average of Pd nanoparticle diameters (*<d*_TEM,N_ *>*) were calculated from the mean diameter of particle size distributions obtained by transmission electron microscopy (TEM; Hitachi, H-9500, and ThermoFisher Themis Z) of at least 100 nanoparticles. Each sample was prepared by grinding the catalyst into a fine powder, dispersed in ethanol, and dripped onto a Cu holey-carbon TEM grid. The surface area normalized average diameter (*<d*_TEM,S_ *>*) for each catalyst was calculated using eq 1.

1 $$<d_{\mathrm{TEM},S}>=\frac{\sum_{i}n_{i}d_{i}^{3}}{\sum_{i}n_{i}d_{i}^{2}}$$

where *n*_*i*_ is the number of nanoparticles with the diameter *d*_*i*_. Figure 1 shows a representative image of a 4 nm Pd nanoparticle with a particle-size distribution histogram.

**Figure 1:**
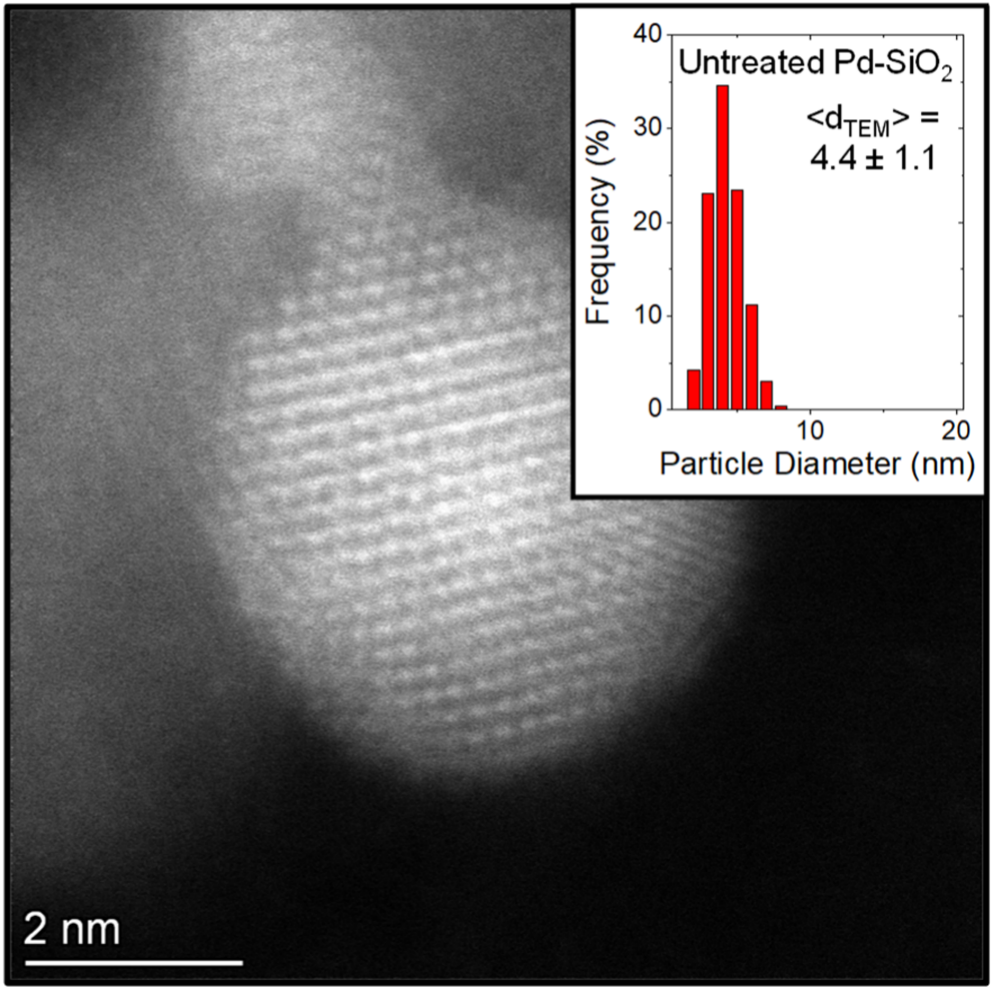
Representative TEM image of 0.04 wt % Pd nanoparticles supported on SiO_2_, in which an inset histogram shows the particle size distribution. Figure S1 shows corresponding images and histograms of particle diameters for the other Pd-based catalysts treated with organic species.

#### 2.2.2 Examination of Pd Surface by Infrared Spectroscopy

*Ex situ* infrared spectra were obtained from Pd samples treated with and without organics, using adsorbed CO as a probe molecule. First, the catalyst was pelletized into a self-supporting disk and loaded into a transmission cell with CaF_2_ windows.^53,61,62^ Background spectra of samples were collected under flowing He, regulated by mass flow controllers. Samples were reduced by a mixture of 4:1 He/H_2_ while heated at 373 K for 1 h. The sample was then cooled in pure He to 303 K, and new background spectra were collected. Then, the gas composition was changed to 50:1 He/CO, and steady-state spectra were collected. Next, the sample was oxidized at 573 K for 1 h in 4:1 He/O_2_. The sample was again cooled in pure He to 303 K, and new background spectra were collected. Then, the gas composition was again changed to 50:1 He/CO, and steady-state spectra were collected. Data were normalized by Si–O–Si overtones as an internal standard from the SiO_2_ support.

*In situ* infrared spectra were obtained using attenuated total reflectance. Here, 10 wt % Pd-SiO_2_ was deposited on a ZnSe crystal and loaded into the instrument. The sample was pretreated at 573 K for 1 h in 4:1 He/O_2_. The sample was then purged with He and treated with 4:1 He/H_2_ at 373 K for 1 h before cooling to room temperature. Separately, two reservoirs of DI H_2_O were sparged with H_2_ and O_2_, which were pumped over the catalyst with liquid flow rates of 3:1, respectively (10 mL min^–1^, 75 kPa H_2_, 25 kPa O_2_, 298 K). Spectra were collected until the sample reached a steady state. Afterward, the pump connected to the H_2_-sparged DI H_2_O was switched to a 5 mM hexaketocyclohexane solution sparged with H_2_ until a new steady state was reached.

#### 2.2.3 Temperature-Programmed Oxidation and Desorption

Temperature-programmed oxidation (TPO) and desorption (TPD) profiles quantified the moles of organic species adsorbed to the catalyst surface. Samples were loaded onto a quartz frit within a fused quartz tube with an embedded thermocouple. A split tube furnace heated the material under 5% O_2_/N_2_ or Ar gas for TPO or TPD, respectively, regulated by a mass flow controller. A mass spectrometer analyzed the effluent gas to quantify CO_2_ evolution (44 *m*/*z*). The sample was purged overnight with the gas at room temperature before measurements. Samples were heated to 973 K at 5 K min^–1^ while monitoring the CO_2_ evolution, which was calibrated using known quantities of NaHCO_3_ (Figure S2) and integrated for the total moles evolved.

#### 2.2.4 Determination of Elemental Composition by EDXRF

The metal loadings were determined by energy-dispersive X-ray fluorescence spectroscopy of Pd-SiO_2_ samples (Table S1). Samples were prepared by loading catalytic materials into sample cups sealed with mylar film. The sample chamber was purged with a He environment and given at least 2 min to purge before conducting the measurement. The molar compositions of Si and Pd were quantified and converted into elemental weight loadings of Pd by assuming all elemental contributions come from Si and Pd signals.

### 2.3 Rate Measurements Using a Fixed-Bed Reactor

Rate and selectivities of H_2_O_2_ formation were measured in a continuous-flow fixed-bed reactor with a cooling jacket (Figure S3).^53,63,64^ The reactor was loaded with catalyst, SiO_2_, glass wool, and glass rods, which were secured and sealed by fritted gaskets. The temperature was controlled by flowing aqueous ethylene glycol through a cooling jacket from a recirculating bath and monitored by a thermocouple. H_2_ and O_2_ compositions in the reactor were controlled by flowing 25% H_2_/N_2_, 99.9% D_2_, or 5% O_2_/N_2_, regulated by mass-flow controllers. An HPLC pump delivered the solvent, which was mixed with gas before contacting the catalyst. The pressure was maintained by a back-pressure regulator controlled by an electronic pressure regulator.

The reactor effluent entered a gas–liquid separator, and the gas was analyzed by a gas chromatograph with a capillary column and thermal conductivity detector using an Ar reference gas. Reactant conversions were compared to calibrated compositions of gas. The liquid fraction was periodically drained by an electronic valve and pushed into a 10-port valve, which injected the liquid and a colorimetric titrant (CuSO_4_, neocuproine) into test tubes. The mixtures were analyzed by UV–vis to quantify H_2_O_2_ concentration using appropriate calibrations. Reported rates were normalized by the total metal content of the materials, and H_2_O_2_ selectivities were calculated by dividing the rate of H_2_O_2_ formation by the rate of H_2_ consumption (*r*_H_2_O_2__/–*r*_H_2__).

Control measurements were conducted using blank SiO_2_, which does not form H_2_O_2_ (Figure S4). Measurements were also performed with SiO_2_ prepared with 1,4-benzoquinone and hexaketocyclohexane equivalent to Pd-SiO_2_ samples (Section 2.1). The redox-active mediators react with the indicator but completely wash away after ∼1 h on stream. All experiments were conducted at a liquid flow rate of 35 cm^3^ min^–1^ to avoid external mass transfer limitations.^53^ All samples satisfy the Madon-Boudart Criterion, enabling meaningful comparisons of catalytic rates and stability without mass transfer constraints.^64–66^ Barrier measurements were corrected by accounting for the deactivation rates.^67^

### 2.4 Rate Measurements Using Semibatch Reactors

H_2_O_2_ formation was measured in round-bottom three-neck flasks in a semibatch configuration (Figure S5).^53^ The flasks were connected to a condenser chilled to 273 K by a recirculating bath. The reactant gases of H_2_ and 5% O_2_/N_2_ were fed through gas dispersion tubes submerged in the solvent and regulated by mass-flow controllers. Measurements were conducted at ambient temperatures and pressures, in which the catalyst slurry was agitated at 1500 rpm by magnetic stir bars to avoid mass transfer limitations.^53^ The solvent was sparged for 10 min before adding the catalyst. Aliquots were extracted periodically to quantify H_2_O_2_ concentrations using the CuSO_4_ indicator (*vide supra*). Concentration profiles were fit to determine rate constants.^53^

### 2.5 Computational Methods

Concise details of the computational methods used are discussed below. More thorough descriptions are provided in the Supporting Computational Methods section.

Homogeneous aqueous phase DFT calculations were carried out using the Gaussian 16 software package to determine the electronic properties of various organic mediators.^68^ The M06–2X exchange-correlation functional,^69^ was used along with the 6–311++G­(d,p) basis set,^70^ and an implicit SMD solvation model for water.^71^

Periodic plane-wave DFT calculations were carried out using the Vienna Ab initio Simulation Package (VASP) to model the mediated reactions of H_2_ and O_2_ on Pd.^72,73^ A slab comprised of a 4 × 4 unit cell with four layers of the Pd(111) surface was used as a model of the exposed Pd facets of the supported Pd nanoparticles. The bottom two layers of the surface were held fixed to the bulk Pd positions while the top two layers were allowed to relax. Previous experimental and computational results suggest steady state coverages of ∼5/16 monolayer (ML) of oxygen (O*) and ∼5/16 ML of subsurface hydrogen (H_s_*), which are similarly considered.^53,74^ As shown in our prior work, the dissociative adsorption of H_2_ has a small barrier and is highly reversible; hence, one surface hydrogen with other surface adsorbates (O*, mediators, O_2_*) has been chosen as a resting state for calculations.^53^

*Ab initio* molecular dynamics (AIMD) simulations were carried out to incorporate explicit solvent molecules in the model and provide more faithful models for the solution phase structures and energies. NVT ensemble dynamics at 300 K were performed with the Nosé-Hover thermostat. A box size of 10 × 10 × 10 Å was first filled with solvent and the individual mediator(s) as an initial guess for the bulk solvation phase of the mediator. AIMD simulations were then carried out for five picoseconds to obtain the bulk solvation phase of the mediators. This equilibrated bulk solvated phase was then used as an initial guess for the solvent and mediator interacting with the Pd(111) surface with the surface O* adsorbates. The condensed phase was simulated by filling the 15 Å vacuum region between the slabs with water molecules with a density of 1 g cm^–3^. This initial interfacial structure was equilibrated for another five picoseconds to allow the explicit solvent molecules to relax around the surface species.

Periodic DFT calculations were carried out using a plane-wave energy cutoff of 400 eV. The Perdew–Burke–Ernzerhof (PBE) functional form of the generalized gradient approximation (GGA) was employed to determine the corrections to the energy due to exchange and correlation effects.^75^ PAW pseudopotentials were used to describe the interactions between the valence and core electrons.^76^ Noncovalent long-range interactions in the system were modeled with the D3 dispersion corrections by Grimme.^77^ The wave functions were self-consistently optimized to within 10^–6^ eV using a 2 × 2 x 1 γ-centered k-point mesh.^78^ The atomic positions were iteratively optimized until the maximum force was less than 0.05 eV/Å. The nudged elastic band (NEB) method was initially used to locate transition states.^79^ Transition states were further refined using the dimer method.^80^

## 3 Results and Discussion

### 3.1 Effect of Organic Species on Rates of H_2_O_2_ Formation

Figure 2 shows rates and selectivities of H_2_O_2_ formation as functions of time-on-stream for Pd-SiO_2_ and two materials formed by treating Pd-SiO_2_ either with 1,4-benzoquinone (BQ) or hexaketocyclohexane (HKH) (200 kPa H_2_, 60 kPa O_2_, 278 K). These materials exhibit an induction period during which rates and selectivities approach nearly constant values over a period of 20 h, suggesting structural or compositional changes in the form of the catalyst. Such changes reflect the aggregation of Pd nanoparticles (Figure S1) or the evolution of organic adsorbates bound to Pd surfaces, similar to our prior findings.^53^ Untreated Pd nanoparticles present greater steady-state turnover rates of H_2_ consumption (−*r*_H_2__) than samples treated with BQ (*r*_H_2__^BQ^/*r*_H_2__^Pd^ = 0.51) or HKH (*r*_H_2__^HKH^/_H_2__^Pd^ = 0.75), which suggests BQ- and HKH-derived species inhibit steps for H_2_ and O_2_ activation. Figure 2a shows H_2_O_2_ formation rates, however, are greater on HKH-treated (*r*_H_2_O_2__^HKH^/*r*_H_2_O_2__^Pd^ = 1.40) and lower on BQ-treated samples (*r*_H_2_O_2__^BQ^/*r*_H_2_O_2__^Pd^ = 0.75) in comparison to untreated Pd nanoparticles. Moreover, Figure 2b shows selectivities of H_2_O_2_ formation over 20 h on stream (200 kPa H_2_, 60 kPa O_2_, 278 K), which reach mean values of 44% for untreated, 64% for BQ-treated, and 83% for HKH-treated Pd nanoparticles in the final 4 h of the measurement. Consequently, adsorbed BQ and HKH-derived species block sites and inhibit O–O bond dissociation paths, consistent with prior observations with organic adsorbates.^32,49,53,81^ Still, the diminished rates of H_2_ activation and increased rates of H_2_O_2_ formation on HKH-treated samples suggest that these organics block surface active sites, but the remaining sites present between the adsorbed organics are much more reactive for forming H_2_O_2_ relative to those on untreated Pd. These observations agree qualitatively with the measured effects of quinone compounds on H_2_O_2_ formation rates and yields upon Pd nanoparticles supported on zeolite β in batch reactors.^82,83^

**Figure 2:**
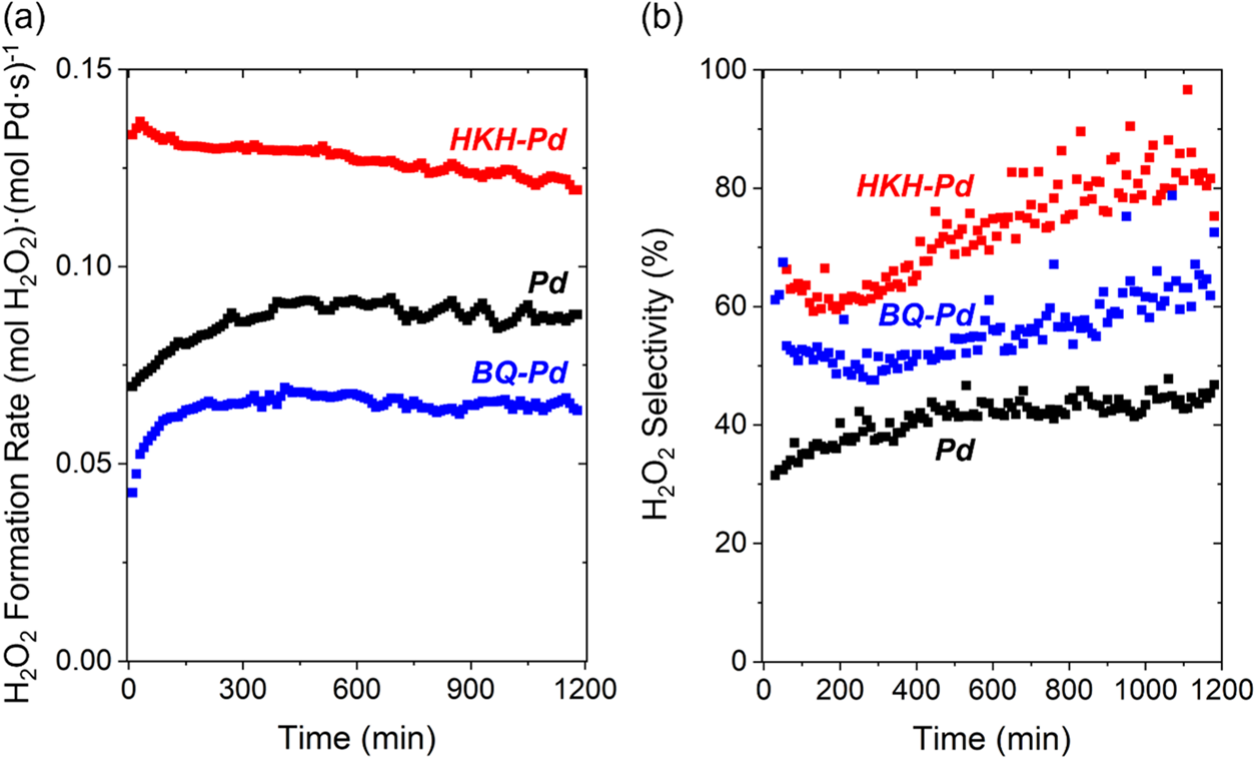
(a) Rates and (b) selectivities of H_2_O_2_ formation as a function of time over SiO_2_-supported Pd nanoparticles without treatment (black) and following treatment with 1,4-benzoquinone (blue) or hexaketocyclohexane (red). Measurements used DI H_2_O as the solvent within a fixed-bed reactor (200 kPa H_2_, 60 kPa O_2_, 278 K).

Figure 3 shows the rates and selectivities of H_2_O_2_ formation over 130 h on stream. The mean H_2_O_2_ selectivity increases to ∼90% in the final 8 h of the measurement on HKH-treated samples. However, the total rate of H_2_O_2_ formation decreased by 32% over this interval, consistent with a loss of Pd content (0.025 to 0.012%, Table S1). In comparison, rates, selectivities, and Pd contents of untreated and BQ-treated Pd catalysts appear stable during reactions in water over similar periods (20 h, Table S1). These findings suggest that HKH leads to the formation of soluble molecular complexes that leach Pd from the catalyst, similarly observed during the dissolution of noble metals in the presence of chelating organic compounds (e.g., acetylacetone) and strong oxidants (e.g., H_2_O_2_, O_2_).^84,85^

**Figure 3:**
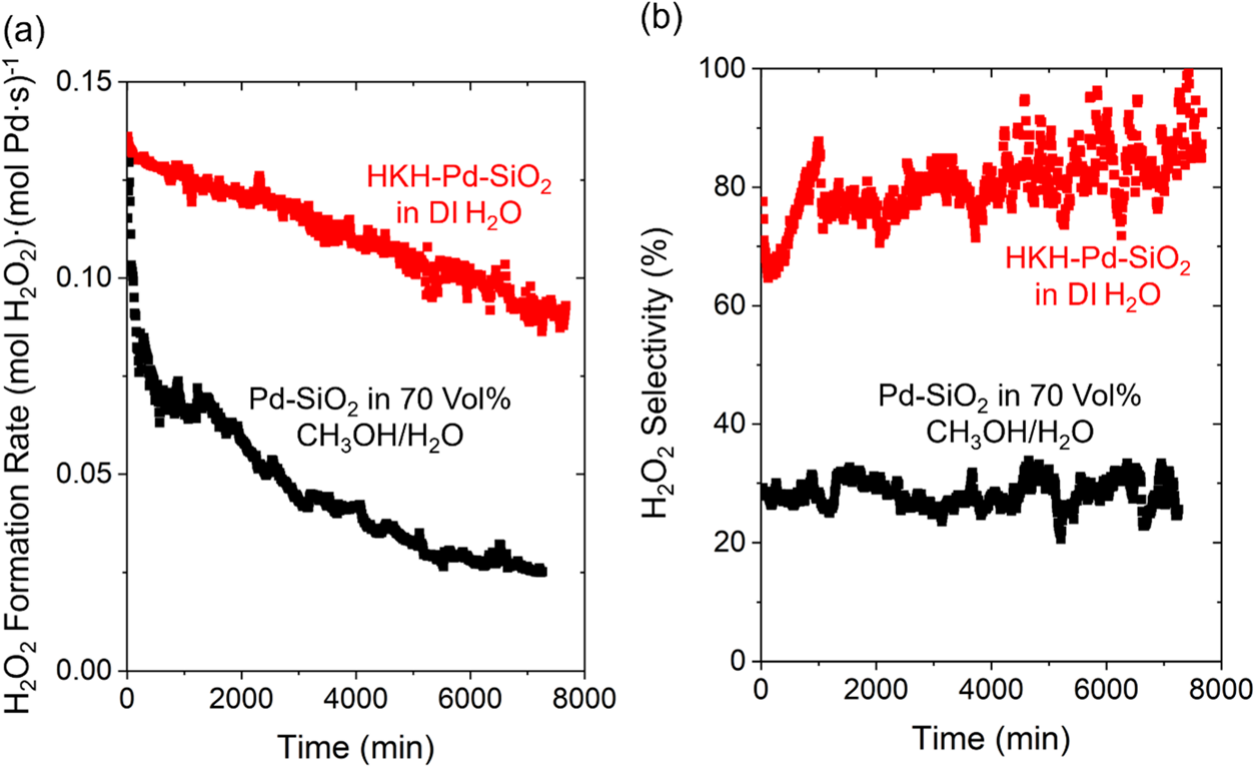
(a) Rates and (b) selectivities of H_2_O_2_ formation as functions of time over SiO_2_-supported Pd nanoparticles without treatment using a 70% (v/v) mixture of methanol and water as the solvent (black) and treated with hexaketocyclohexane using DI H_2_O as the solvent (red). Measurements were within a fixed-bed reactor (200 kPa H_2_, 60 kPa O_2_, 278 K).

Prior studies identified a mixture of 70% by volume methanol in water as an optimal solvent for H_2_O_2_ synthesis in batch reactors over short periods (∼0.5 h).^86–89^ Although alcohol and aqueous alcohol solvent yield higher initial rates of H_2_ consumption in comparison to water, alcohol-derived residues accumulate on Pd nanoparticles and inhibit reactions of H_2_ and O_2_.^53,81,86,90^ For example, rates of H_2_O_2_ formation on Pd-SiO_2_ decrease by 78% over 130 h in 70% vol. methanol in water, while giving low mean H_2_O_2_ selectivities (28%) in the final 8 h of the experiment. In comparison, HKH-modified Pd-SiO_2_ provides greater rates, selectivities, and stability than Pd-SiO_2_ catalysts operating within methanol solvent or aqueous methanol solutions. Thus, modifying Pd nanoparticles with organic species provides a strategy to decouple the advantages of organic solutions (higher initial rates and selectivities of H_2_O_2_ formation) from their drawbacks (rapid deactivation, costly and environmentally impactful organic solvent) over much longer reaction intervals.

In contrast to reactions conducted in excess hydrogen, O_2_-rich conditions (60 kPa H_2_, 100 kPa O_2_, 278 K; Figures S6 and S7) result in lower selectivities to H_2_O_2_ on untreated Pd (18%), BQ-treated (24%), and HKH-treated (27%) Pd nanoparticles in the final 4 h of the 20 h time-on-stream measurement. These observations correlate with *in situ* X-ray absorption spectra showing excess O_2_ stabilizes the metallic Pd while excess H_2_ favors PdH_*x*_ formation,^91–93^ which in our prior studies was found to stabilize O–O bonds and improve H_2_O_2_ selectivity.^53^ The presence of BQ- and HKH-derived species still leads to greater H_2_O_2_ selectivities; however, the absolute selectivities and the fractional improvements are more significant in H_2_-rich conditions (200 kPa H_2_, 60 kPa O_2_, 278 K; Figure 2b), which suggests the higher coverages of reactive oxygen found upon metallic Pd surfaces decompose or destabilize the BQ- and HKH-derived organic species. Cyclic changes between H_2_- (200 kPa H_2_, 60 kPa O_2_) and O_2_-rich (60 kPa H_2_, 100 kPa O_2_) conditions at 278 K leads to initially lower H_2_O_2_ selectivities on organic-treated samples (e.g., 83 to 53% for HKH-Pd-SiO_2_) upon returning to H_2_-rich conditions for 2 h (Figure S8). However, the H_2_O_2_ selectivities return to the previous steady-state values after a 20 h period (Figure S9), which indicates the regeneration of the selective form of the catalyst. In contrast, untreated Pd samples recover their steady-state selectivities within less than 1 h after returning to H_2_-rich conditions. These comparisons suggest that there may be a pool of quinone- and hydroquinone-derived intermediates on the Pd surface that undergoes reversible changes upon exposure to high coverages of oxygen and hydrogen-derived surface species.

Still, the species that show the greatest H_2_O_2_ formation rates and selectivities only form at high H_2_ pressure and require many hours to recover the composition and catalytic structures responsible for the selective reduction of O_2_ to H_2_O_2_ on Pd.

In contrast to expectations, H_2_O_2_ formation rates and selectivities differ only weakly between catalysts that use distinct 1,4-benzoquinone analogs, with the exceptions of chlorine and additional carbonyl groups (*vide infra*). Furthermore, polyaromatic species (e.g., 1,4-naphthoquinone, 1,4-anthraquinone; Figures S11–S13) do not introduce significant differences relative to 1,4-benzoquinone. These comparisons suggest that inductive effects that impact the extent of electron withdrawal from carbonyl groups do not significantly impact catalysis or that these functional groups speciate *in situ* (e.g., hydrogenolysis of C–X bonds results in loss of heteroatoms). By comparison, catalysts formed by adsorption of benzene analogs (C_6_X_4_H_2_; X = H, CH_3_, F, Cl) to Pd nanoparticles also improve H_2_O_2_ selectivities (Figures S14 and S15), despite the absence of carbonyl groups needed to provide redox activity from the initial polyconjugated structure. Similarly, the adsorption of C_1_–C_3_ aldehydes and ketones (e.g., formalin, acetone) increases H_2_O_2_ selectivities relative to unmodified Pd (Figure S16), albeit to a lesser extent than observed on the cyclic organic compounds.^53^ In contrast, Pd nanoparticles treated with aliphatic or ether species (e.g., *n*-hexane, tetrahydrofuran, or dioxane) show negligible differences in reactivity compared to untreated samples (Figure S17).

Taken together, these observations give evidence that carbonyl groups, aromatics, and polyconjugated rings facilitate strong adsorption of organic species to Pd surfaces where redox active complexes form *in situ* by pathways that cleave C–X bonds and form C–O bonds to produce surface complexes with a chemical function analogous to quinones. This interpretation agrees with prior reports that Pd nanoparticles catalyze the oxidation of benzene to phenol (423–473 K)^94–97^ and the reduction of quinones to hydroquinones (313–343 K).^60,98,99^ These findings suggest that Pd introduces oxygen to aromatic rings and subsequently forms hydroxyl groups. Indeed, these conclusions agree with our theoretical calculations showing that aromatics bind strongly to Pd surfaces and transform quickly to hydroxylated species (*vide infra*). Consequently, these results are consistent with quinones, aromatics, and carbonyl compounds reacting with Pd nanoparticles to form persistent organic surface species that mediate reactions of H_2_ and O_2_ to produce H_2_O_2_.

While most analogs of benzene and 1,4-BQ provide similar selectivities for H_2_O_2_ formation, adsorption of tetrachloro-1,4-benzoquinone, 1,2,4,5-tetrachlorobenzene, or HKH leads to statistically significant increases in H_2_O_2_ selectivities (Figures 4 and S10). These differences implicate the *in situ* formation of adsorbate structures distinct from other polyconjugated and aromatic compounds. For example, adsorption of chloride and bromide to Pd nanoparticles increases H_2_O_2_ selectivities,^86,100–104^ potentially due to the disruption of contiguous ensembles of Pd atoms needed to dissociate O–O bonds. Here, the modest increases in H_2_O_2_ selectivities are consistent with the *in situ* speciation of chlorine (i.e., breakage of C–Cl bonds) from the chlorinated BQ analogs and their subsequent adsorption to Pd nanoparticle surfaces (Figure 4). Note, however, that fluorine atoms speciated from fluorinated analogs have much smaller ionic radii and weaker effects on selectivity, a trend similar to those reported in studies comparing halides during H_2_O_2_ formation on Pd.^104^ Regardless, this speciation hypothesis does not account for greater rates and selectivities for H_2_O_2_ formation with HKH (i.e., in the presence of additional carbonyl moieties). Rather, these observations suggest that the quinones react with hydrogen to form partially hydrogenated quinones (HQ) with −OH functional groups. These species likely stabilize or react with O_2_-derived intermediates (e.g., O_2_*, OOH*) and promote the formation of H_2_O_2_, as was described for the *in situ* formation of hydroxymethyl species on Pd derived from methanol.^55^

**Figure 4:**
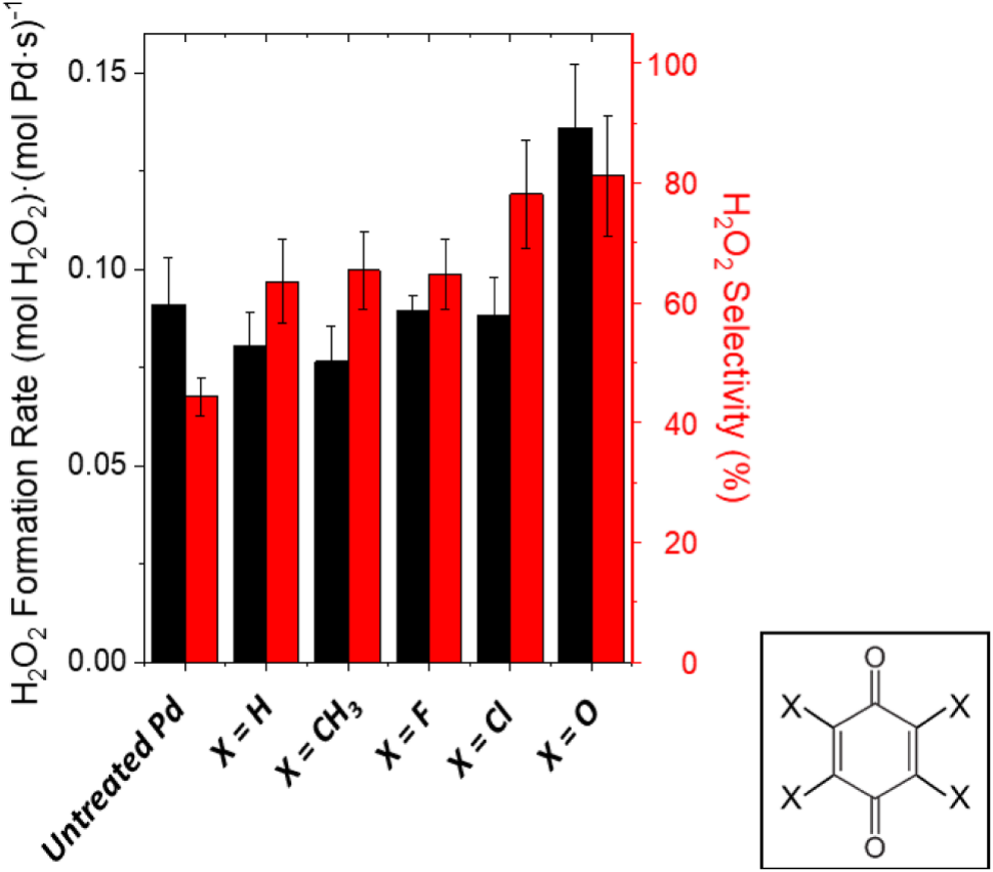
Average rates (black) and selectivities (red) of H_2_O_2_ formation over SiO_2_-supported Pd nanoparticles without treatment and following treatment with analogs of 1,4-benzoquinone (C_6_X_4_O_2_) with different functional groups (X = H, CH_3_, F, Cl, O). Measurements used DI H_2_O as the solvent within a fixed-bed reactor (200 kPa H_2_, 60 kPa O_2_, 278 K), in which rates were averaged over 1200 min on stream. Figure S10 shows the corresponding time-on-stream measurements.

### 3.2 Quinone Species Adsorb Strongly to Pd Surfaces

Figure 5 shows rates and selectivities of H_2_O_2_ formation averaged over 1200 min on stream, which increase with increasing concentrations of quinone species (1,4-benzoquinone, 1,4-naphthoquinone, 1,4-anthraquinone). These rates and selectivities approach constant values with sufficiently large concentrations of quinones, suggesting these species form complete monolayer coverages on the Pd surfaces. Such observations agree with temperature-programmed oxidation (TPO) conducted on materials following reactions for 1200 min on stream (200 kPa H_2_, 60 kPa O_2_, 278 K), showing that BQ-treated and HKH-treated Pd nanoparticles (Figure S18a) evolve ∼10-times the quantity of CO_2_ (303–423 K) as observed for untreated Pd-SiO_2_ materials, indicating the oxidation of persistent organic species from Pd nanoparticles. Control experiments (Figure S19) show that the TPO of untreated SiO_2_ samples evolves CO_2_ only at temperatures above 423 K (Figure S19a), consistent with the oxidation of adventitious organics on the silica surface. Moreover, temperature-programmed desorption (TPD) shows that similar quantities of CO_2_ evolve (303–423 K) from organic-treated and untreated samples (Figure S18b) when heated in flowing helium, which reflects desorption of atmospheric CO_2_ or oxidation of organic species by strongly bound oxygen on Pd surfaces but not SiO_2_ (Figure S19b). Still, TPD profiles show the evolution of significantly fewer moles of CO_2_ than in TPO profiles, indicating that most of the CO_2_ evolution on organic-treated samples comes from the oxidation of BQ- or HKH-derived species. Thus, organic species saturate the surface of Pd nanoparticles and persist over extended periods of catalysis.

**Figure 5:**
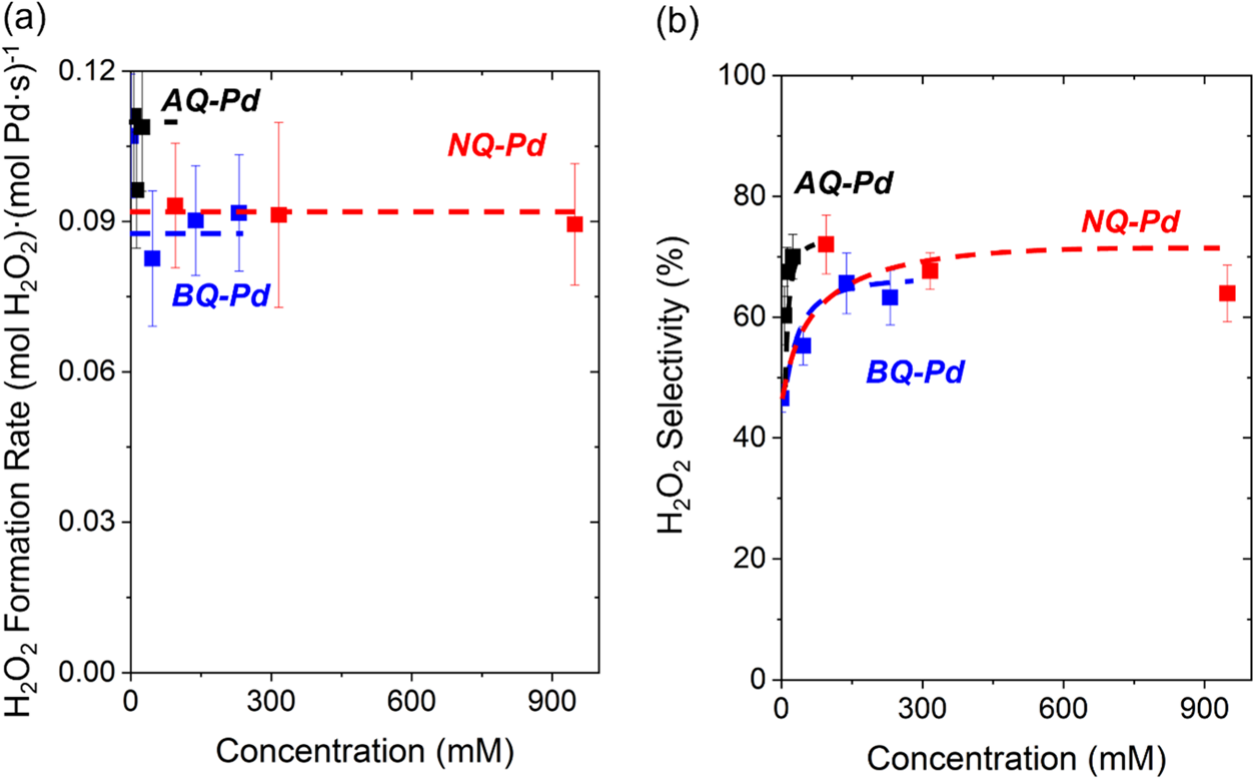
Average (a) rates and (b) selectivities of H_2_O_2_ formation over SiO_2_-supported Pd nanoparticles functionalized with increasing concentrations of 1,4-benzoquinone (blue), 1,4-naphthoquinone (red), 1,4-anthraquinone (black) before catalysis. Measurements used DI H_2_O as the solvent within a fixed-bed reactor (200 kPa H_2_, 60 kPa O_2_, 278 K). Figures S11–S13 show the corresponding time-on-stream measurements.

Figure 6 shows *ex situ* infrared spectra of CO adsorbed on Pd-SiO_2_ samples after catalysis. Before the adsorption of CO, each material was treated in flowing H_2_ (21 kPa H_2_, 373 K, 1 h) to reduce oxidized Pd and remove H_2_O. Multiple vibrational features appear between 2015–2175 cm^–1^ that correspond to CO adsorbed at atop sites on Pd and features below 2000 cm^–1^ that reflect CO bound to bridge sites between Pd atoms.^61,62,105^ Spectra of adsorbed CO on each material following catalysis (200 kPa H_2_, 60 kPa O_2_, 278 K, 20 h) appear similar, but the relative intensity of atop and bridging CO features differ compared to untreated Pd samples. Specifically, the line shape and peak centers of atop CO show minor changes between fresh Pd (2088 cm^–1^) and spent samples of Pd (2090 cm^–1^), BQ-Pd (2092 cm^–1^), and HKH-Pd (2093 cm^–1^). Similarly, the bridging CO feature (∼1910 cm^–1^) appears broad and unimodal on all catalysts. Thus, organic residues may form upon the Pd surfaces either from the intentional addition of organic species or by adventitious carbon compounds that bind to untreated surfaces.

**Figure 6:**
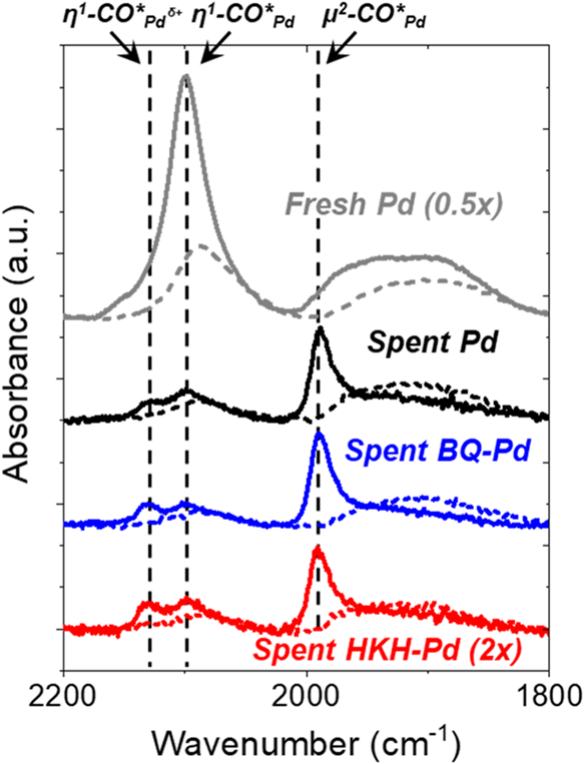
*Ex situ* infrared spectra of CO-saturated surfaces of SiO_2_-supported Pd nanoparticles (0.04 wt % Pd, 0.02 kPa CO, 303 K) freshly prepared (gray) and after catalysis within a fixed-bed reactor (200 kPa H_2_, 60 kPa O_2_, 278 K) for 20 h. Spent samples include those loaded in the reactor without treatment (black) and with treatment of 1,4-benzoquinone (blue) or hexaketocyclohexane (red). Spectra were collected after initial reduction (20 kPa H_2_, 373 K) for 1 h (dotted lines) and subsequent oxidation (20 kPa O_2_, 573 K) and reduction (20 kPa H_2_, 573 K) for 1 h each (solid lines).

Next, samples were oxidized (21 kPa O_2_) and reduced (21 kPa H_2_) at 573 K to remove organic species remaining on the sample. Figure S20 shows the difference in background spectra before and after this subsequent thermal treatment, showing the removal of adventitious carbon and H_2_O remaining on the material. However, the thermal pretreatments used to clean the Pd metal before FTIR measurements may irreversibly change the organic species on Pd. Upon CO exposure, all catalysts show changes in line shape and an increase in total peak area compared to the initial reductive treatment, which is consistent with the emergence of a greater quantity of sites that bind CO after high-temperature oxidation. Notably, new features emerge that agree with CO adsorbed atop oxidized atoms of Pd (∼2133 cm^–1^) and CO bridging undercoordinated ensembles of Pd (∼1990 cm^–1^).^105–109^ Since these sites only appear after high-temperature oxidation, they likely bind adsorbates strongly and may be blocked by organic species during catalysis, whether added intentionally or not. Nevertheless, the line shape and ratio of the integrated peak area of bridging and atop CO are nearly identical for each spent sample (Figure 6 and Table S2), consistent with the similar particle size distribution of these catalysts (Figure S1). Still, this ratio increases between fresh and spent materials, corroborating the agglomeration of Pd nanoparticles from a diameter of ∼4 to ∼9 nm after catalysis (Figure S1). As such, the Pd nanoparticles of the final samples are morphologically similar irrespective of their modification with HKH or BQ, suggesting they do not influence the core structure of nanoparticles. Thus, the electronic state of Pd is likely similar between each sample, indicating quinones affect catalysis by altering reactions on the nanoparticle surface.

Furthermore, we sought to better understand the *in situ* transformation of these species upon adding mediators. Figure S21 shows that the addition of HKH leads to growth of broad features consistent with alkoxy (υ­(C–O) at 1060 cm^–1^), aromatic (υ­(C=C) at 1540, 1640, and 1690 cm^–1^), carbonyl (υ­(C=O) at 1770 cm^–1^), and hydroxyl functions (υ­(O–H) at 3200 cm^–1^), distinct from molecular HKH.^96^ Taken together, these spectra suggest that HKH adsorbs to the surface of Pd and reacts with hydrogen to generate hydroxyl functions. However, HKH intermediates may also decompose a variety of carbonylic and other organic species on the Pd surface, which react with H_2_ and O_2_, similarly observed for methanol speciation in our prior work.^53^

### 3.3 Dependence of Rates on Isotopic Compositions

These results show that organic species bind to Pd nanoparticles and modify oxygen reduction paths over extended periods of catalysis. Still, these data do not distinguish whether these changes result from site blocking or the introduction of new reaction pathways. Thus, we performed isotopic measurements to understand if the adsorption of organic species influences the mechanism of proton transfer reactions, as reported in our prior work.^55^ Table 1 shows the relative rates of hydrogen activation and oxygen reduction paths over HKH-treated and untreated Pd nanoparticles using H_2_ and D_2_ reactants and H_2_O and D_2_O solvents. Rates of oxygen reduction decrease when using D_2_ versus H_2_ as the reductant (200 kPa H_2_ or 200 kPa D_2_, 60 kPa O_2_, 278 K), which implicates kinetically relevant reactions of H_2_ (Figure S22). Here, the kinetic isotope effects of H_2_O_2_ formation on HKH-treated (*k*_H_2__/*k*_D_2__ = 1.17 ± 0.03) and untreated (*k*_H_2__/*k*_D_2__ = 1.18 ± 0.01) surfaces of Pd appear similar. Yet, D_2_ lowers rates of H_2_O formation by a greater extent for HKH-treated (*k*_H_2__/*k*_D_2__ = 2.5 ± 0.5) than untreated (*k*_H_2__/*k*_D_2__ = 1.5 ± 0.2) samples. Additionally, rates of H_2_O_2_ formation (*k*_H_2_O_/*k*_D_2_O_ = 1.0 ± 0.1) and decomposition (*k*_H_2_O_/*k*_D_2_O_ = 1.0 ± 0.2) on untreated Pd do not respond to the isotope substitution of the solvent water. Together, these observations on untreated Pd nanoparticles indicate that heterolytic hydrogen activation and oxidation occur in kinetically relevant processes; however, proton transfer steps from water do not affect rates.^53,110^ In contrast, rates on HKH-treated Pd samples respond to changes from perhydrogenated to perdeuterated water solvent and give clear kinetic isotope effects for H_2_O_2_ formation (*k*_H_2_O_/*k*_D_2_O_= 1.3 ± 0.4) and decomposition (*k*_H_2_O_/*k*_D_2_O_ = 1.8 ± 0.7). These differences imply that the presence of HKH-derived residues introduces new reaction pathways that involve kinetically relevant proton transfer steps that do not occur in water.

**Table 1:** Effect of Isotopic Substitution on Rate Constants for the Formation of H_2_O_2_ and H_2_O on Silica-Supported Pd Nanoparticles before and after Treatment with Hexaketocyclohexane Using H_2_ or D_2_ Reactants within a Fixed-Bed Reactor (200 kPa H_2_ or D_2_, 60 kPa O_2_, 278 K; Figure S22)­^a^

	H_2_/D_2_		H_2_O/D_2_O	
*k*_H_/*k*_D_	Pd	HKH-Pd	Pd	HKH-Pd
H_2_ + O_2_ → H_2_O_2_	1.18 ± 0.01	1.17 ± 0.03	1.0 ± 0.1	1.3 ± 0.4
H_2_ +1/2O_2_ → H_2_O	1.5 ± 0.2	2.5 ± 0.5		
H_2_O_2_ + H_2_ → 2H_2_O			1.0 ± 0.2	1.8 ± 0.7

a Complementary H_2_O_2_ formation and decomposition measurements on the same material using H_2_O or D_2_O solvents (80 mL) within a semibatch reactor (4.8 kPa H_2_, 4.8 kPa O_2_, 298 K). Figure S23 shows the transient concentration profiles of H_2_O_2_ formation used to fit these rate constants.

These observations align with our theoretical calculations (*vide infra*) and observed differences in catalytic performance and kinetic isotope effects observed between reactions among H_2_ and O_2_ on Pd nanoparticles in pure water and in aqueous organic solutions.^53^ Those earlier results demonstrated product formation rates depend sensitively on isotope substitution (i.e., H_2_O/D_2_O, and H_2_/D_2_), which implicated hydroxymethyl intermediates (CH_2_OH*) in reactions that mediated PCET steps that reduced O_2_*, OOH*, and O* surface intermediates. Here, the carbonyl groups of HKH (and similar functions of other organic species examined) seem likely to convert to −OH/D functions by reaction with H*/D*, and these functions can rapidly exchange protons and deuterons with the solvent (H_2_O, D_2_O). Consequently, the reduction of O_2_-derived surface species to hydrogen peroxide or water occurs at lower rates in D_2_O due to the kinetically relevant transfer of deuterons from HKH-derived species. Without organic residues, rates in D_2_O and H_2_O are indistinguishable. Indeed, recent findings show measurable kinetic isotope effects for oxygen reduction with related quinone compounds over M–N–C catalysts within solutions of H_2_O or D_2_O,^57^ which corroborate this interpretation. These observations support the hypothesis that HKH and other polyconjugated carbonyl and aromatic compounds adsorb to Pd nanoparticles and form surface redox mediators (e.g., (CO)_6_H*) that provide new reaction pathways for the catalytic reduction of O_2_ with H_2_ that favor the retention of O–O bonds and formation of H_2_O_2_.

### 3.4 Effect of Organic Adsorbates on Reaction Barriers

Quinone and carbonyl species block sites and introduce new proton transfer paths that favor H_2_O_2_ formation, consistent with these species stabilizing transition states that convert OOH* to H_2_O_2_. Rates of H_2_O_2_ and H_2_O formation were measured as a function of temperature to calculate apparent activation enthalpy barriers for H_2_O_2_ (Δ*H*_H_2_O_2__^‡^) and H_2_O (Δ*H*_H_2_O_^‡^) formation, which were then compared to DFT-calculated values using model Pd surfaces, representative of experiments.

Quantitative descriptions of reaction rates help elucidate the mechanistic interpretation of these barrier measurements (Figure S24), as shown by

2 $$-r_{H_{2}}=r_{H_{2}O}+r_{H_{2}O_{2}}$$

where the rates of H_2_O_2_ (*r*_H_2_O_2__) and H_2_O (*r*_H_2_O_) formation equal that of H_2_ consumption (−*r*_H_2__). Under the conditions used here, prior work suggests that these rates can be described by

3 $$r_{H_{2}O_{2}}=k_{H_{2}O_{2}}\left[O_{2}\right]$$

4 $$r_{H_{2}O}=k_{H_{2}O}\left[O_{2}\right]$$

where *r*_H_2_O_2__ and *r*_H_2_O_ depend on the apparent rate constants of H_2_O_2_ (*k*_H_2_O_2__) and H_2_O (*k*_H_2_O_) formation, increase in proportion to the pressure of O_2_ (i.e., [O_2_]), and remain independent of the pressure of H_2_. Complete derivations of eqs 2–4 are presented in detail elsewhere.^53,63^ Briefly, the values of *k*_H_2_O_2__ and *k*_*H*_2_ *O*_ reflect the relative preference of hydroperoxyl (OOH*) to reduce to H_2_O_2_ or dissociate to H_2_O, respectively. Here, all paths that form H_2_O occur irreversibly, as evidenced by studies showing that mixtures of H_2_, ^16^O_2_, and ^18^O_2_ form H^16^O^16^OH and H^18^O^18^OH but not H^16^O^18^OH.^111^ Thus, the formation of H_2_O_2_ requires the preservation of O–O bonds.

The tenets of transition state theory describe the relationship between the ratio of H_2_O_2_ and H_2_O formation rates and the free energy differences between the apparent activation free energies as

5 $$\frac{r_{H_{2}O_{2}}}{r_{H_{2}O}}=\frac{k_{H_{2}O_{2}}}{k_{H_{2}O}}=\frac{e^{-\Delta G_{H_{2}O_{2}}^{‡}/\mathrm{RT}}}{e^{-\Delta G_{H_{2}O}^{‡}/\mathrm{RT}}}=e^{\Delta S_{H_{2}O_{2}}^{‡}-\Delta S_{H_{2}O}^{‡}/R}•e^{\Delta H_{H_{2}O}^{‡}-\Delta H_{H_{2}O_{2}}^{‡}/\mathrm{RT}}$$

where the difference in transition state Gibbs free energies of H_2_O_2_ (*G*_H_2_O_2__^‡^) and H_2_O (Δ*G*_H_2_O_^‡^) formation reflects the difference in the apparent values of Δ*H*_H_2_O_2__^‡^ and Δ*H*_H_2_O_^‡^ (i.e., ΔΔ*H* ^‡^ = Δ*H*_H_2_O_^‡^ – Δ*H*_H_2_O_2__^‡^) and their entropic contributions (Δ*S*_H_2_O_2__^‡^,Δ*S*_H_2_O_^‡^).

Similar forms of expressions describe the rate of H_2_ consumption at high coverages of H*, which appears as

6 $$-r_{H_{2}}=k_{H_{2}}\left[O_{2}\right]\propto e^{-\Delta G_{H_{2}}^{‡}/\mathrm{RT}}\left[O_{2}\right]\propto e^{-\Delta H_{H_{2}}^{‡}/\mathrm{RT}}\left[O_{2}\right]$$

where the H_2_O_2_ and H_2_O formation rates sum to the apparent rate constant of H_2_ reactivity (*k*_H_2__), reflecting the apparent Gibbs free energy (Δ*G*_H_2__^‡^) and enthalpy (Δ*H*_H_2__^‡^) of activation for H_2_ consumption.

Table 2 shows values for apparent activation enthalpies for H_2_O_2_ and H_2_O formation and H_2_ consumption on untreated Pd nanoparticles and those treated with BQ or HKH. Measurements were conducted at reaction conditions where rates do not depend on the pressure of H_2_ (200 kPa H_2_, 60 kPa O_2_, 278–308 K), which align with the forms of eqs 3–6.^53^ Values of Δ*H*_H_2__^‡^ are greater in the presence of either BQ- or HKH-derived surface species, which suggests these complexes inhibit the activation of H_2_. Comparisons among values of Δ*H*_H_2_O_2__^‡^ and Δ*H*_H_2_O_^‡^ demonstrate that barriers for H_2_O_2_ formation remain nearly constant within the uncertainty of the measurements. However, barriers for H_2_O formation via steps that cleave O–O bonds increase significantly. These differences agree with slight differences in H_2_O_2_ formation rates and notable increases in H_2_O_2_ selectivities caused by the adsorption of HKH and other organic intermediates (Figures 2–4). These observations may reflect the strong adsorption of organic species that interrupt contiguous ensembles of Pd atoms and likely destabilize transition states and product states for reactions that cleave O–O bonds. However, differences in measured kinetic isotope effects suggest that organic species also modify the mechanisms for reactions of H_2_ and O_2_ by introducing steps that transfer protons and electrons through these complexes.

**Table 2:** Apparent Activation Enthalpies of H_2_O_2_ (Δ*H*_*H*_2_ *O*_2__^‡^) and H_2_O (Δ*H*_*H*_2_ *O*_^‡^) Formation and H_2_ (Δ*H*_*H*_2__^‡^) Activation over Untreated Pd Nanoparticles and Pd Nanoparticles Treated with Either 1,4-Benzoquinone (BQ) or Hexaketocyclohexane (HKH)^a^

material	Δ*H*_H_2_O_2__^‡^ (kJ mol^–1^)	Δ*H*_H_2_O_^‡^ (kJ mol^–1^)	Δ*H*_H_2__^‡^ (kJ mol^–1^)
untreated Pd-SiO_2_	8 ± 3	19 ± 3	15 ± 2
BQ-treated Pd-SiO_2_	9 ± 1	36 ± 5	20 ± 2
HKH-treated Pd-SiO_2_	12 ± 5	46 ± 10	27 ± 9

a Measurements used DI H_2_O as the solvent within a fixed-bed reactor (200 kPa H_2_, 60 kPa O_2_, 278–308 K). Figure S24 shows the corresponding rate measurements as a function of temperature.

Plausible reaction mechanisms and the effects of quinone adsorption on surface reactions were evaluated with DFT calculations carried out on a model Pd(111) surface (the dominant facet of Pd nanoparticles). The calculations examined surfaces that possessed adsorbed surface O* and subsurface H_s_* (similar to adlayer structures determined in our prior work (Figure S25)^53^ together with the reactive quinone species (e.g., C_6_O_6_*)). The adsorption of these mediators on the surface was initially examined using implicit water solvation, after which the surfaces were solvated with explicit water molecules, and AIMD simulations were conducted to determine the lowest-energy structures for reactive surface species and hydrogen bonding networks at the solid–liquid interface. The models constructed in this manner were used to determine reaction energies (Δ*E*_rxn_) and intrinsic barriers (Δ*E* ^⧧^) for relevant elementary steps (Figures 7 and 8).

**Figure 7:**
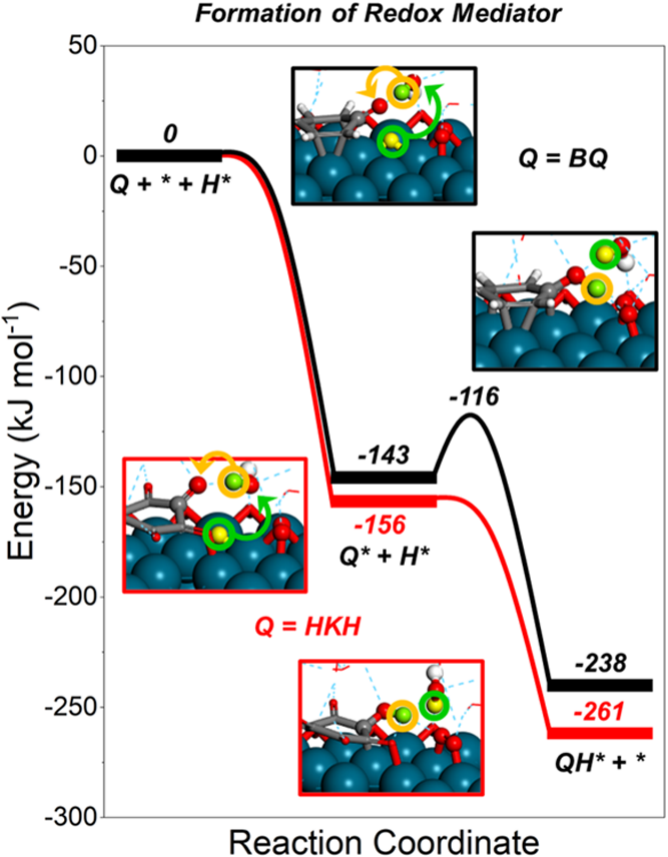
Reaction coordinate diagrams showing DFT-calculated energies for forming partially hydrogenated quinone-based redox mediator by adsorption and reaction with surface hydrogen by water-mediated steps on Pd(111)-based surfaces. The simulated Pd(111) surfaces contain adsorbed O* (5/16 ML) and subsurface H_s_* (5/16 ML) to represent the state of Pd nanoparticles at reaction conditions determined previously by *operando* EXAFS.^53^ The green circles show hydrogen atoms heterolytically oxidized to form protons (and electrons), and the yellow circles show the proton (and electron) transfer.

**Figure 8:**
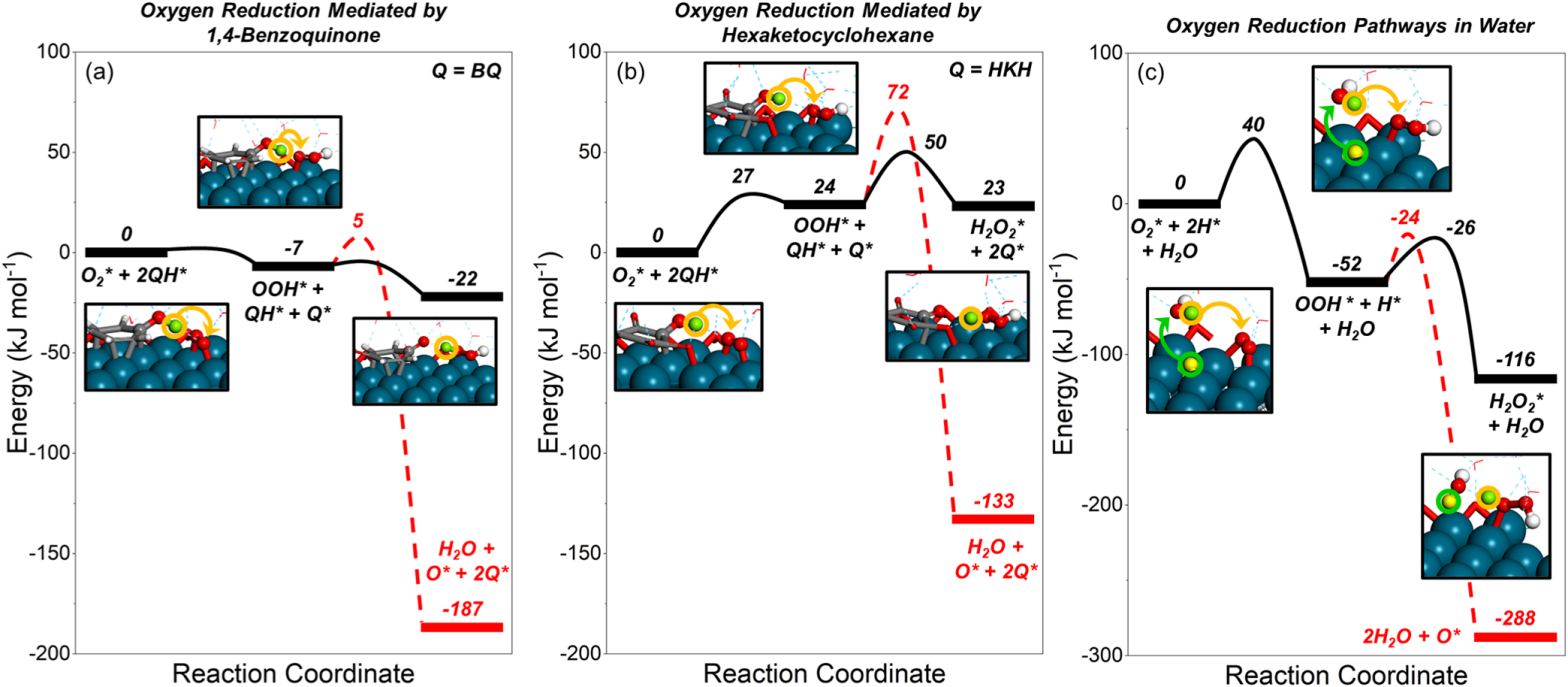
Reaction coordinate diagrams showing DFT-calculated energies for the catalytic formation of H_2_O_2_ (black solid lines) and H_2_O (red dashed lines) mediated by (a) partially hydrogenated 1,4-benzoquinone, (b) partially hydrogenated hexaketocyclohexane, and (c) solution-phase water molecules on Pd(111)-based surfaces. The simulated Pd(111) surfaces contain adsorbed O* (5/16 ML) and subsurface H_s_* (5/16 ML) to represent the state of Pd nanoparticles at reaction conditions determined previously by *operando* EXAFS.^53^ The green circles show hydrogen atoms heterolytically oxidized to form protons (and electrons), and the yellow circles show the proton (and electron) transfer.

Quinones and other polyconjugated carbonyl and aromatic species bind to the surface by Pd–C (hydroxy) or Pd–O (alkoxy) bonds. Here, the hydroxy species were more reactive and formed more readily, a conclusion similar to that drawn for reactions of methanol-derived adsorbates.^53^ Figures S26–S28 show the binding modes and adsorption energies of aliphatic and polyconjugated compounds (e.g., quinones, aromatics) to the Pd surface (i.e., Q + * → Q*), showing highly exothermic adsorption. HKH presents the most exothermic adsorption energy (Δ*E*_ads_ = −156 kJ mol^–1^) of all quinone compounds, while BQ binds slightly weaker (Δ*E*_ads_ = −143 kJ mol^–1^). Aromatics (e.g., benzene) adsorb with comparable but slightly weaker energies (Δ*E*_ads_ = −125 kJ mol^–1^). Moreover, these aromatics readily form hydroxylated species (e.g., phenols, quinones) by direct OH-transfer paths (Δ*E* ^⧧^ = 74 kJ mol^–1^; Figure S29b), in which the net reaction is highly exothermic (Δ*E*_rxn_ = −202 kJ mol^–1^; Figure S29a). Thus, DFT calculations support the strong binding of polyconjugated carbonyl and aromatic species to Pd surfaces, which agree with their persistence during extended periods of reaction (Sections 3.1 and 3.2).

Hydrogenation of one or more of the carbonyl bonds of the surface-bound quinones (Q*) can form the partially hydrogenated (HQ*) or multihydrogenated (e.g., H_2_Q*, H_n_Q*) quinone species. Hydrogenation steps can occur by direct reactions of the quinone and surface-bound hydrogen (i.e., H* + Q* → HQ* + *) or by PCET steps mediated by water molecules, i.e., H* + H_2_O + Q* → HQ* + H_2_O + *; Figures S30a,b, S31a,b, and S7. Mechanisms involving direct surface hydrogenation of the quinone occur more readily for HKH (Δ*E* ^⧧^ = 28 kJ mol^–1^) than for BQ (Δ*E* ^⧧^ = 39 kJ mol^–1^). In comparison, barriers for hydrogenation by water-mediated PCET steps show similar trends (i.e., HKH < BQ) but present significantly lower values for HKH (Δ*E* ^⧧^ = 0 kJ mol^–1^) and BQ (Δ*E* ^⧧^ = 27 kJ mol^–1^) than for direct hydrogenation mechanisms (Figure 7). These findings suggest that rates of quinone hydrogenation (reduction) by paths mediated by water molecules exceed those of direct hydrogenation by H atoms, an implication consistent with measured kinetic isotope effects (Table 1). Notably, H_2_O_2_ and H_2_O formation rates in perhydrogenated water exceed those in perdeuterated water, suggesting that PCET processes with kinetically relevant proton transfer prevail over other paths in the presence of HKH. This conclusion agrees with computational studies (Figures S33–S36), which predict weak kinetic isotope effects between H_2_O/D_2_O (*k*_H_/*k*_D_ = 1.02) and H_2_/D_2_ (*k*_H_/*k*_D_ = 1.18) without a mediator. Similarly, computation suggests that HKH can significantly increase the maximum possible kinetic isotope effect (*k*_H_/*k*_D_ = 5.74). However, the equilibration of protons and deuterons with the mediators likely attenuates this effect, but it still agrees with the emergence of kinetic isotope effects when adding mediators.

The Pd surface binds H* and O_2_* species that can react to form OOH* *via* direct surface hydrogenation (i.e., H* + O_2_* → OOH* + *), PCET mediated via a partially hydrogenated surface-bound quinone (i.e., QH* + O_2_* → Q* + OOH*), or by PCET mediated by water molecules (i.e., H* + H_2_O + O_2_* → OOH* + H_2_O + *). Again, the direct hydrogenation of O_2_* from surface H* presents the highest barrier for OOH* formation (Δ*E* ^⧧^ = 51 kJ mol^–1^; Figure S32a). The reduction of O_2_ by the partially hydrogenated quinone-mediated steps shows barriers more than 20 kJ mol^–1^ lower (Figures 8a,8b, S30c, and S31c). Here, the barrier for reacting O_2_* with partially hydrogenated BQ (Δ*E* ^⧧^ = 0 kJ mol^–1^) remains lower than that for reactions with partially hydrogenated HKH (Δ*E* ^⧧^ = 27 kJ mol^–1^). The reduction of O_2_* by water-mediated PCET steps shows higher barriers (Δ*E* ^⧧^ = 40 kJ mol^–1^; Figures 8c and S32b). Thus, PCET mechanisms that involve quinone-derived surface redox mediators show the most accessible pathways to reduce O_2_* on the surface of Pd. Moreover, it is likely that partially hydrogenated quinones are rapidly consumed by these fast O_2_ reduction steps (*vide infra*); hence, multihydrogenated species were not considered further.

Subsequent steps reduce OOH* species to H_2_O_2_ or H_2_O by similar PCET mechanisms (Figure 8b). However, the quinone-mediated oxygen reduction routes are less exothermic than the water-mediated routes since most of the energy loss occurs during the initial hydrogenation of the mediator (Figure 7). Regarding selectivity, the water-mediated PCET steps show similar barriers to forming both H_2_O_2_ (Δ*E*_H_2_O_2__^⧧^ = 26 kJ mol^–1^) and H_2_O (Δ*E*_H_2_O_^⧧^ = 28 kJ mol^–1^) (Figures 8c and S32c,d) without quinone-derived intermediates. By comparison, the partially hydrogenated HKH-derived mediator offers PCET pathways with a barrier for H_2_O_2_ formation (Δ*E*_H_2_O_2__^⧧^ = 26 kJ mol^–1^) equal to that of water; however, the barrier for HKH to mediate H_2_O formation (Δ*E*_H_2_O_^⧧^ = 48 kJ mol^–1^) significantly exceeds that for water-mediated paths and all other partially hydrogenated quinone species (Figures 8a,8b, S30d,e, and S31d,e). Furthermore, the differences between the barriers to form H_2_O versus H_2_O_2_ with the partially hydrogenated HKH mediator (ΔΔ*E* ^⧧^ = Δ*E*_H_2_O_^⧧^ – Δ*E*_H_2_O_2__^⧧^ = 22 kJ mol^–1^) yield the greatest kinetic preference for selective H_2_O_2_ formation. These energetics represent a considerable increase over the change in barriers to form H_2_O_2_ and H_2_O *via* water-mediated PCET steps (ΔΔ*E* ^⧧^ = 2 kJ mol^–1^).

These calculated values for the differences in the barriers of H_2_O_2_ and H_2_O formation (ΔΔ*E* ^⧧^) agree with the trends observed for experimentally measured barriers. Table 2 compares the differences between apparent activation enthalpies to form H_2_O_2_ and H_2_O for HKH- (ΔΔ*H* ^⧧^ = Δ*H*_H_2_O_^⧧^ – Δ*H*_H_2_O_2__^⧧^ = 34 kJ mol^–1^), BQ- (ΔΔ*H* ^⧧^ = 27 kJ mol^–1^), and water-mediated paths (ΔΔ*H* ^⧧^ = 11 kJ mol^–1^). However, mathematical treatments of the apparent barriers of reactions reflect an interdependence of H_2_ adsorption, quinone reduction, and oxygen reduction steps, which complicate direct quantitative comparisons between the absolute values of experimental (Δ*H*_H_2_O_2__^⧧^) and computed activation barriers (e.g., Δ*E*_H_2_O_2__^⧧^). Regardless, experimental barriers, kinetic isotope measurements, and the interpretation of these values informed by *ab initio* calculations provide strong evidence that quinones bind to catalyst surfaces and form mediators, which present favorable barriers that enable the greatest rates and selectivities of H_2_O_2_ formation compared to untreated Pd nanoparticles (Figures 2 and 4).

### 3.5 Influence of Mediator Chemical Structure

First-principles homogeneous-phase DFT calculations were subsequently used to provide insight into how the chemical structure of the mediators relates to their catalytic performance (Figures 9 and S37–S38 and Table S3). In general, electron-donating substituents on BQ decrease the reduction potential of BQ derivatives, thus lowering the thermodynamic favorability of adding an electron to the mediator. Comparisons of one- and two-electron reduction potentials show that HKH possesses the most positive reduction potential of all mediators investigated and indicate that HKH most readily accepts electrons. HKH also shows the smallest energy gap between the lowest unoccupied molecular orbital (LUMO) and the highest occupied molecular orbital (HOMO), which further suggests that HKH is the most electrophilic among these mediators.

**Figure 9:**
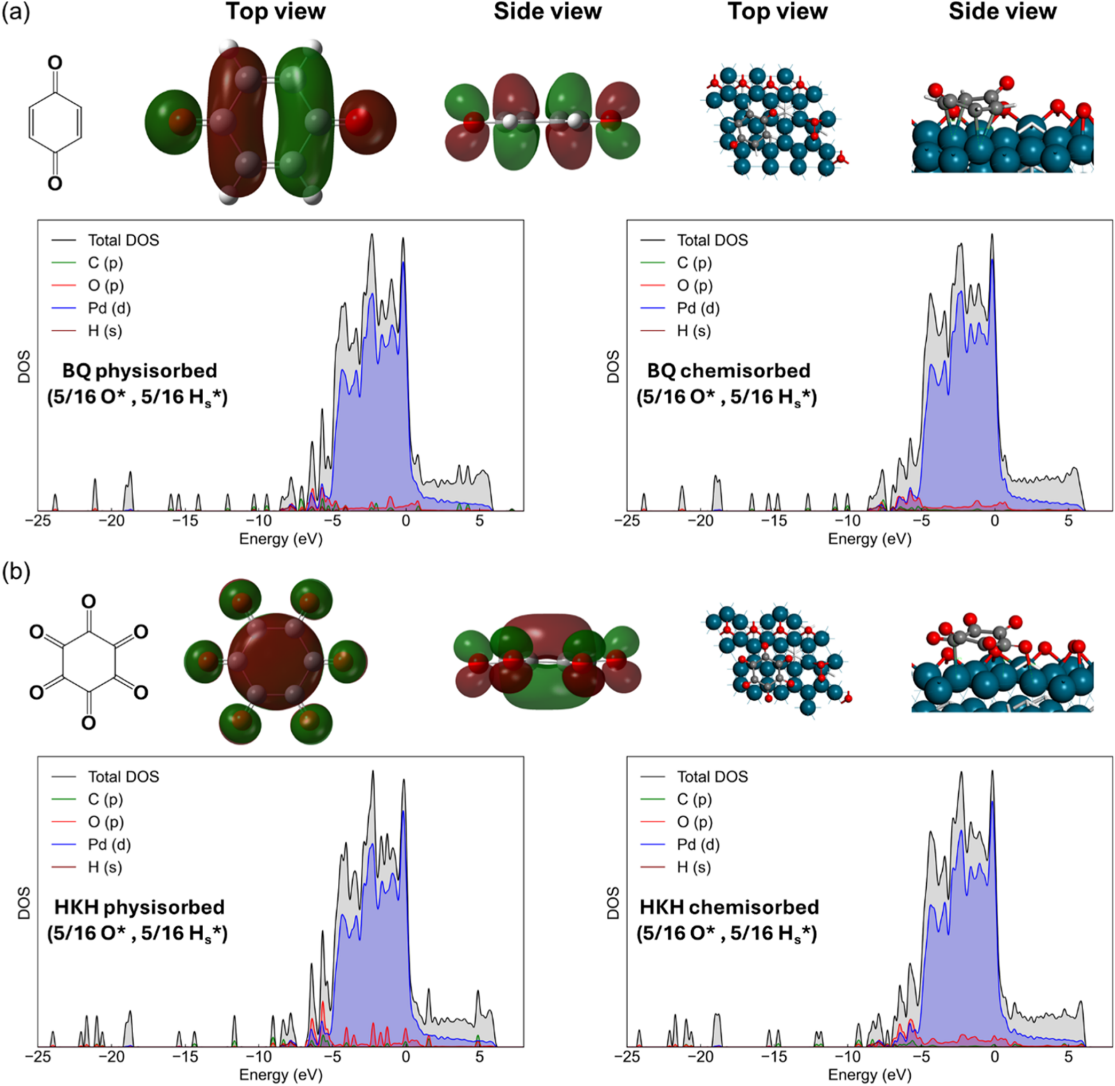
Top and side views of the lowest unoccupied molecular orbitals and the resulting distinct surface chemisorption modes for (a) BQ and (b) HKH, in which palladium, oxygen, carbon, and hydrogen are represented by the teal, red, gray, and white spheres, respectively. The orbital colors, green and maroon, show the opposite phases of the wave function in each region of the mediators. The plots below show the partial density of states for physisorbed and chemisorbed BQ and HKH for (5/16) ML O* and (5/16) ML H_s_* coverage in an implicit solvent. The total DOS are black, the PDOS for d states of Pd are blue, the p states of O are red, the p states of C are green, and the s states of H are maroon. The shading under each region is proportional to the total number of electrons that can occupy each type of orbital.

The trends among these characteristics align with comparisons of the partial densities of states (PDOS) for physisorbed and chemisorbed mediators bound to Pd in the presence of coadsorbed O* and subsurface H_s_* (Figures 9 and S39–S43). Specifically, chemisorption of HKH results in a significant intensification, shift, and delocalization of the molecular contributions of the p-states from C- and O-atoms that overlap with d-band states of Pd near the Fermi level. These changes exceed those that occur when BQ chemisorbs to an equivalent surface (Figures S39–S43). These comparisons show that HKH gives the greatest extent of orbital intermixing between Pd and the organic adsorbate of the mediators examined, in line with its most electrophilic nature established previously, which explains the highly exothermic adsorption of HKH to Pd surfaces (Figure S26).

The electronic properties of the mediators correlate to intrinsic barriers for PCET reactions with coadsorbed surface species. The most electrophilic mediators (i.e., HKH) present the lowest barriers for the heterolytic oxidation of surface H* species and concurrently give the highest barriers for PCET to reduce O_2_* and OOH* species compared to less electrophilic species (Figure 8). These trends reflect the greater extent of electron withdrawal from the Pd surface, which decreases the length of C–Pd and O–Pd bonds between Pd and mediators, consistent with stronger adsorption energies. These differences in charge transfer between electrophilic mediators and surface Pd atoms lower the extent of electron back-donation from Pd to the 2π* orbitals of O_2_*, which shorten O–O bond lengths, increase O–O bond dissociation barriers, and increase H_2_O_2_ selectivities.

*Ab initio* calculations also explain the need to operate at H_2_-rich conditions to maintain the surface redox mediators on Pd. Increases in the surface coverage of O* from 2/16 ML to 8/16 ML weaken the adsorption energies of all mediator species (Figure S26) by differences that span from 43 to 116 kJ mol^–1^. Higher coverages of O* block more surface sites and reduce the number of bonds between Pd and the mediator, diminishing the covalent interaction between the mediator and the surface. The PDOS calculations support this interpretation and show that lower coverages of O* atoms delocalize (i.e., broaden) the molecular contributions of p-states from C- and O-atoms of HKH and BQ that overlap with the d-band states of Pd near the Fermi level (Figures S39 and S42–S43). Thus, low coverages of O* result in greater hybridization among the p orbitals of the mediator with the d orbitals of Pd surface atoms. By comparison, subsurface H_s_* has a much weaker effect on the adsorption energies of mediators (Figure S26) and PDOS features of these species (Figures S39–S41). Although O* destabilizes the redox mediators, O* does not affect the density of surface electrons or reduce the extent of electron back-donation to π* 2p orbitals of O_2_* as significantly as the mediators. Such findings seem consistent with the lower H_2_O_2_ selectivities under O_2_-rich conditions, even in the presence of surface-bound mediators (Figure S8). Thus, H_2_-rich conditions maintain selective PdH_*x*_ phases^53^ and also preserve the strong binding and electronic structure of bound redox mediators that favor the reduction of O_2_ without cleaving O–O bonds.

These electronic structure calculations agree with prior findings, kinetic isotope measurements (Table 1), and the requirement of high hydrogen pressures to promote H_2_O_2_ formation (Figures 2, S6, and S8).^53^ Together, these findings appear most consistent with a mechanism in which mediators adsorb strongly to Pd surfaces, react readily with surface H* species, form O–H moieties, and transfer protons and electrons to O_2_* and OOH* species during the formation of H_2_O_2_. These calculations demonstrate several key points. First, PCET paths universally show lower barriers for transferring hydrogen atoms to the bound oxygenates (i.e., quinones, O_2_-derived species) than direct hydrogenation routes. Second, more electrophilic mediators (e.g., HKH) bind most strongly to Pd, react most readily with H* species, and best preserve O–O bonds for the selective formation of H_2_O_2_. Third, high coverages of O* species block sites and disrupt the selective electronic state of quinone mediators, necessitating H_2_-rich conditions to maintain the promoting effects of organic adsorbates.

## 4 Conclusions

Rate measurements, kinetic isotope effects, and calculated reaction pathways show that quinone and carbonyl species modify Pd surfaces and undergo cocatalytic redox reactions with H_2_ and O_2_, leading to greater selectivities of H_2_O_2_ formation (∼65–85%) than on unmodified surfaces (∼45%). Specifically, FTIR spectra, TPO profiles, and DFT calculations show the adsorption of organic molecules onto Pd nanoparticles that persist over extended periods of catalysis (>130 h). These species stabilize O–O bonds under H_2_-rich conditions (200 kPa H_2_, 60 kPa O_2_) but not O_2_-rich conditions (60 kPa H_2_, 100 kPa O_2_), consistent with oxygen disrupting the binding of organic mediators and surface electron density needed for selective H_2_O_2_ formation under H_2_-rich conditions. Moreover, these species lead to the emergence of kinetic isotope effects distinct from those on untreated Pd catalysts, consistent with organic moieties facilitating kinetically relevant proton transfer reactions. Such findings agree with DFT calculations showing that the carbonyl functions of quinones heterolytically oxidize hydrogen atoms that subsequently transfer to oxygen species by PCET mechanisms. Furthermore, transition state calculations and temperature dependence measurements show that these paths present favorable barriers of forming H_2_O_2_ while obstructing O–O dissociation reactions that generate H_2_O. Thus, this approach enables stable and selective H_2_O_2_ formation using H_2_O as the solvent, allowing sustainable H_2_O_2_ synthesis without organic solvents.

These findings provide a strategy to introduce persistent cocatalytic moieties onto metal nanoparticles that activate H_2_ and O_2_, which guide the design of catalysts for H_2_O_2_ synthesis and other redox reactions. This work, combined with prior studies, shows how quinone, carbonyl, and alcohol molecules adsorb to Pd nanoparticles and act as redox mediators. Generally, these species must (1) adsorb to catalytic surfaces more strongly than they dissolve into solution, (2) remain kinetically stable under both reductive and oxidative conditions, (3) withdraw electron density from metal surfaces to stabilize O–O bonds, and (4) introduce low-barrier PCET paths at solid–liquid interfaces. Such species may enable similar redox reactions on transition metal surfaces or alloys. Moreover, these species may facilitate other heterolytic hydrogenation and hydrogenolysis steps, which may reduce other polar heteroatoms, such as C–N, C–O, and N–O bonds in thermal and electrochemical systems.^112^ Thus, this work presents opportunities to tailor the active sites of metallic nanoparticles by introducing surface mediators that cocatalyze a broad range of chemical transformations.

## Supplementary Material





## Notes

Additional characterization of materials by transmission electron microscopy, energy dispersive X-ray fluorescence, infrared spectroscopy, and temperature-programmed desorption and oxidation measurements. Details of experimental rate studies, including process flow diagrams, additional controls, time-on-stream measurements, kinetic isotope studies, and Arrhenius plots. Details of theoretical calculations, including modeled surface structures, molecular electronic structures, redox potentials, partial density of states, kinetic isotope effects, and calculations of energies of adsorption, reaction, and transition states (PDF)
Computational structure files for this work (ZIP)

## ∥

Department of Chemical and Biomolecular Engineering, Rice University, 6100 Main St., Houston, Texas 77030, United States

## ⊥

Amogy, 19 Morris Ave, Brooklyn, New York 11250, United States.
