# Supplementary material for: Intentional Formation of Persistent Surface Redox Mediators by Adsorption of Polyconjugated Carbonyl Complexes to Pd Nanoparticles
Source: J Am Chem Soc. 2025 Apr 4;147(20):16885–900. doi: 10.1021/jacs.4c15874 (PMC12100725; doi:10.1021/jacs.4c15874)
Supplement: Supplementary file 1 [file ja4c15874_si_001.pdf]

Intentional Formation of Persistent Surface Redox Mediators by Adsorption of Polyconjugated Carbonyl Complexes to Pd Nanoparticles

Jason S. Adams,[1] Mayank Tanwar,[2] Haoyu Chen,[1,3] Sucharita Vijayaraghavan,[1,3] Tomas Ricciardulli,[1] Matthew Neurock,[2*] David W. Flaherty[1,3*]

[1]Department of Chemical and Biomolecular Engineering, University of Illinois at Urbana-Champaign, IL, 61801, USA

[2] Department of Chemical Engineering and Materials Science, *University of Minnesota, Minneapolis, MN, 55455, USA.*

[3] School of Chemical and Biomolecular Engineering, Georgia Institute of Technology, GA, 30332, USA

*To whom correspondence should be addressed. Email: dflaherty3@gatech.edu (DWF); mneurock@umn.edu (MN)

Table S1. Characterization of Pd nanoparticle diameters from TEM histograms ($d_{TEM,S}$ and $d_{TEM,N}$) shown in Figure S1 and Pd metal content from elemental analysis calculated by EDXRF. Pd samples were prepared using strong electrostatic adsorption of $(NH_4)_2PdCl_4$ on SiO_2 as the catalyst precursor.

Sample	$d_{TEM,S}$ (nm)	$d_{TEM,N}$ (nm)	EDXRF Pd metal content (%)
Fresh Untreated Pd-SiO_2	4.4 ± 1.1	3.8 ± 1.1	0.044 ± 0.009
Fresh BQ-treated Pd-SiO_2	-	-	0.043 ± 0.006
Fresh HKH-treated Pd-SiO_2	-	-	0.025 ± 0.006
Spent Untreated Pd-SiO_2	9.1 ± 2.0	8.1 ± 2.0	0.044 ± 0.006
Spent BQ-treated Pd-SiO_2	9.3 ± 2.1	8.2 ± 2.1	0.043 ± 0.004
Spent HKH-treated Pd-SiO_2	9.5 ± 2.1	8.5 ± 2.1	0.012 ± 0.005

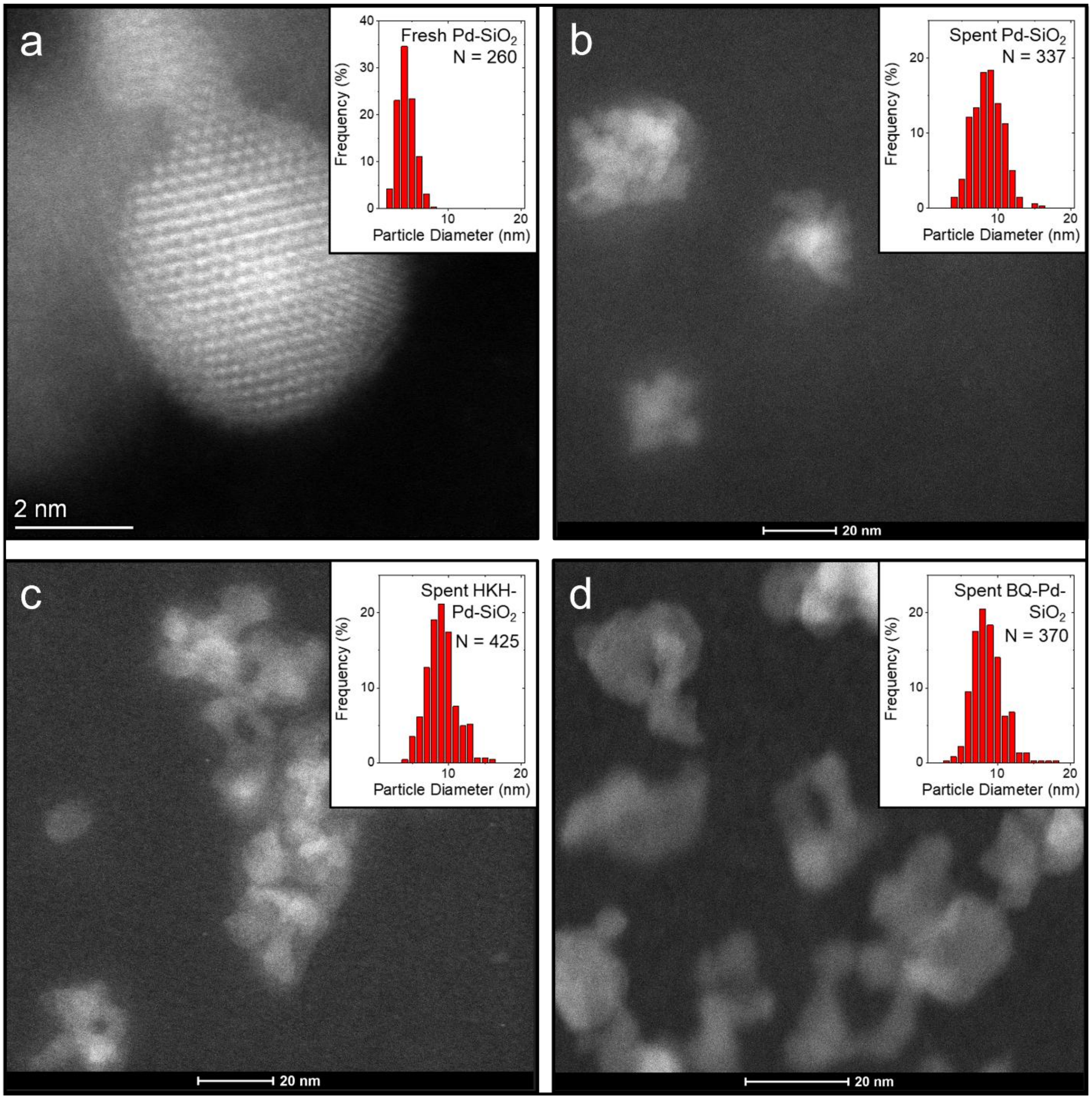

Figure S1. Representative TEM images of a Pd nanoparticles supported on SiO_2 for untreated (a, b), BQ-treated (c), and HKH-treated (d) samples before (a) and after (b, c, d) reactions within a fixed bed-reactor (200 kPa H_2, 60 kPa O_2, 278-308 K) following 20 hours on stream. More than 100 particles were measured to calculate the value of $d_{TEM,S}$ and $d_{TEM,N}$, which are the surface area-averaged and averaged diameters reported in Table S1, respectively.

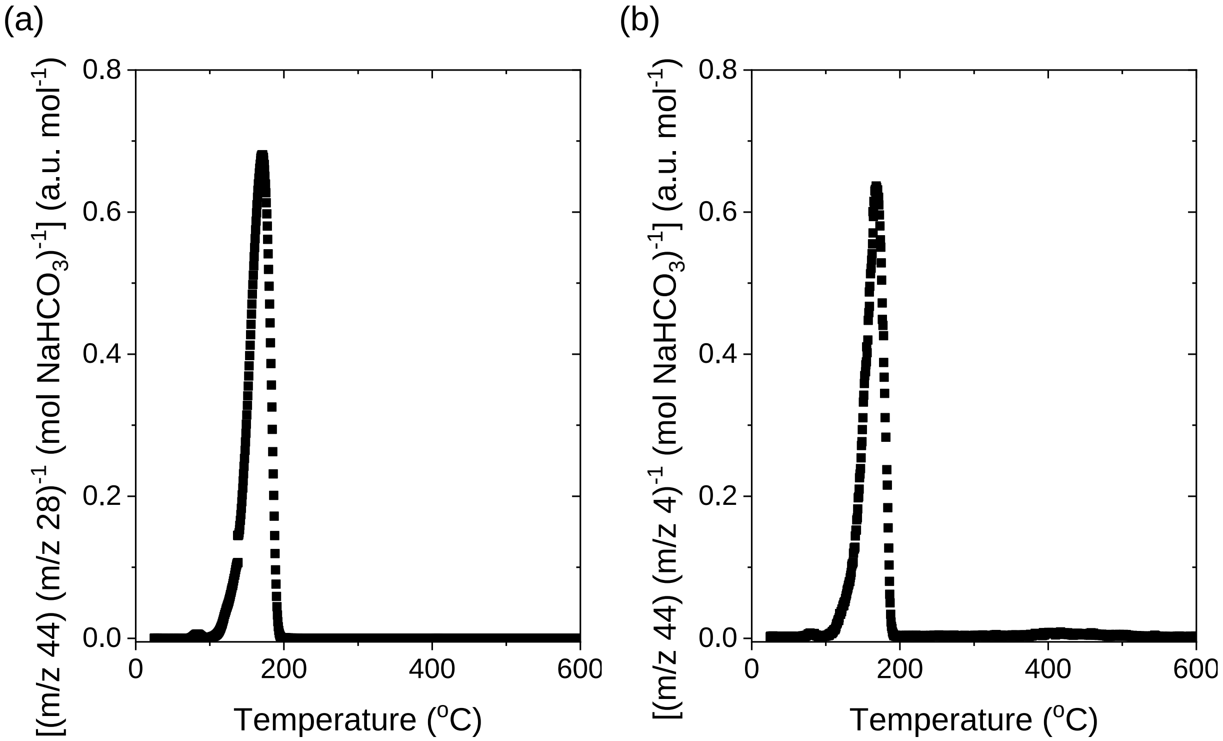

Figure. S2. (a) Temperature programmed oxidation (5 kPa O_2, 96 kPa N_2), and (b) temperature programmed desorption (101 kPa He) profiles for the decomposition of $NaHCO_3$ as a function of temperature within a quartz tube furnace (5 K min^{-1}, 100 cm^3 min^{-1}, 298-973 K). The reported signal intensity reflects the ratios of counts for CO_2 and N_2 or CO_2 and He.

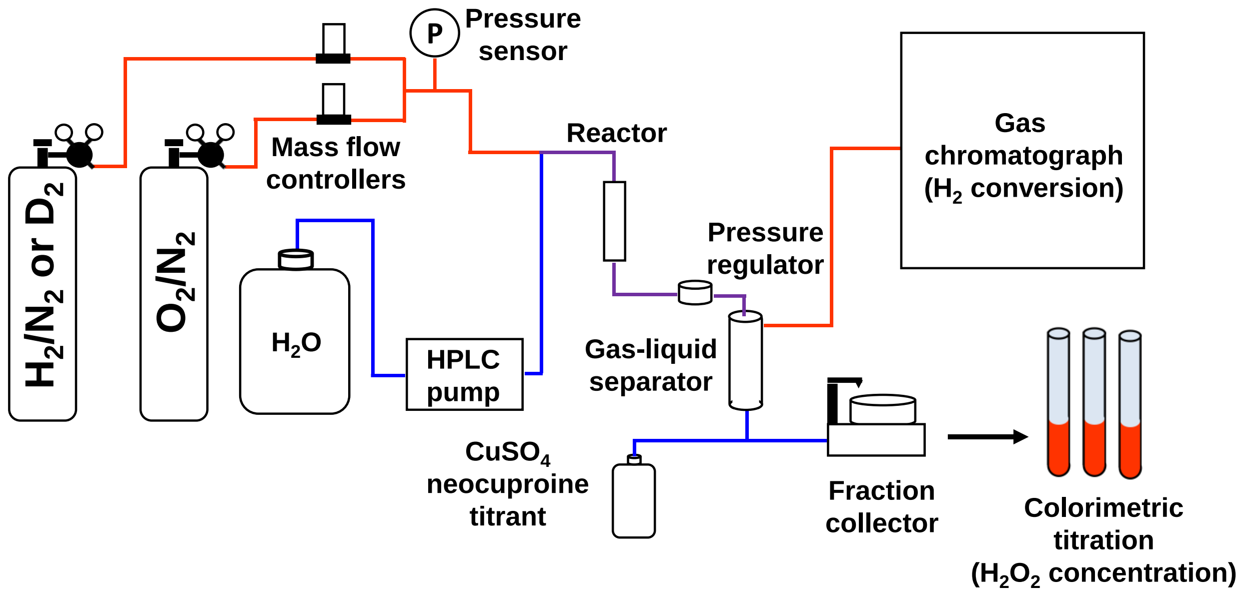

Figure. S3. A schematic of the trickle bed reactor used for the continuous measurements in this study. Here, a mixture of H_2, D_2, O_2, and N_2 flow into the system from mass flow controllers, and this gas mixes with solvent pumped from the liquid carboy. This mixture was passed over the catalyst bed at the desired reactant pressure, maintained by a back-pressure regulator. The mixture was separated in a gas-liquid separator, after which the composition of each phase was analyzed to determine product compositions.

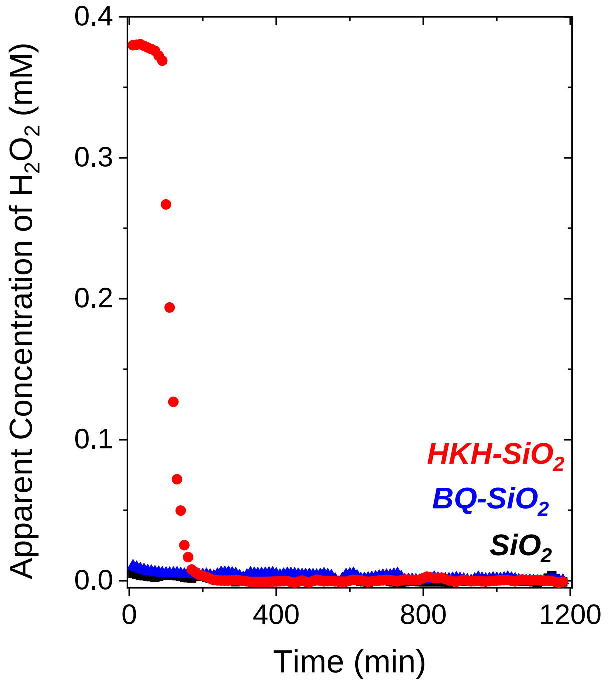

Figure S4. Apparent H_2O_2 concentration as a function of time over SiO_2 without treatment (black ■) and following treatment with 1,4-benzoquinone (blue ▲) or hexaketocyclohexane (red ●), equivalent to Pd catalysts. Measurements used DI H_2O as the solvent within a fixed-bed reactor (200 kPa H_2, 60 kPa O_2, 278 K) flowing at 35 mL min^{-1}, as used in catalytic measurements.

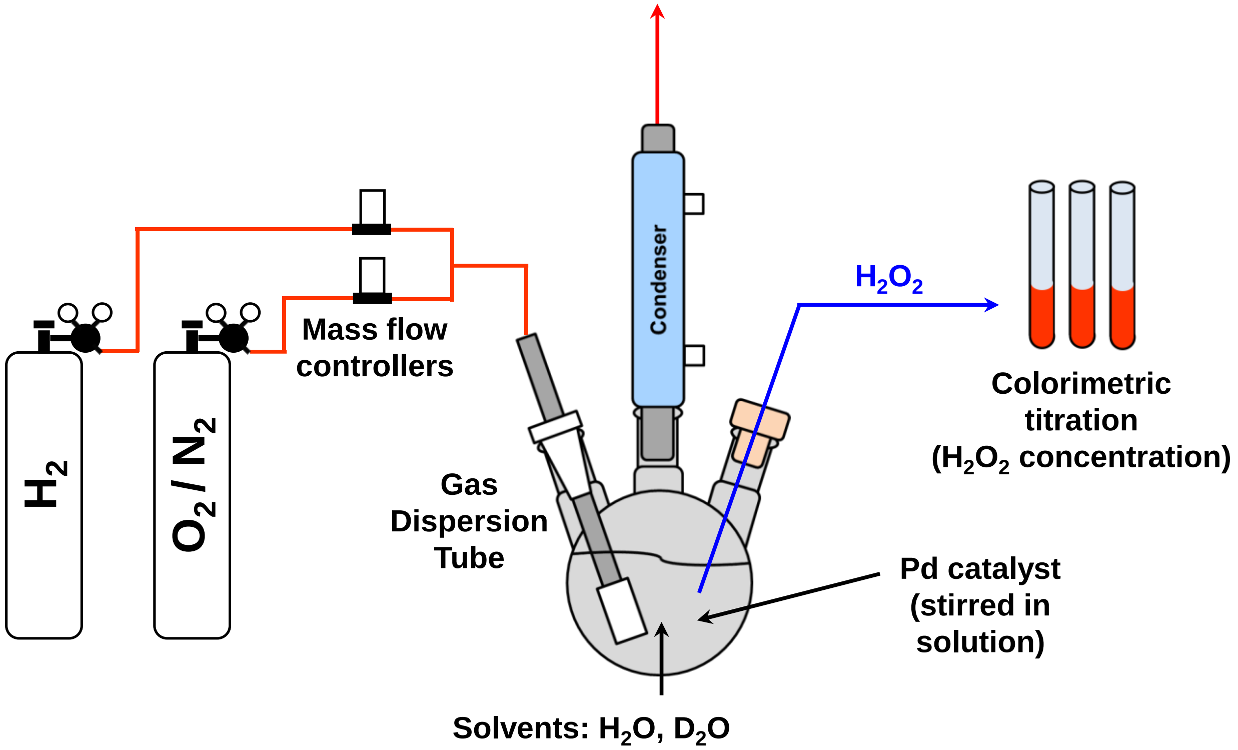

Figure. S5. Process flow diagram of the semi-batch reactor set up used for the transient kinetic isotope and solvent identity measurements in this study. Here, a mixture of H_2, O_2, and N_2 flow into the system from mass flow controllers and mix within three-neck flasks. The gas reacts with the slurry of catalyst and solvent to produce H_2O_2, which was detected by colorimetric titration.

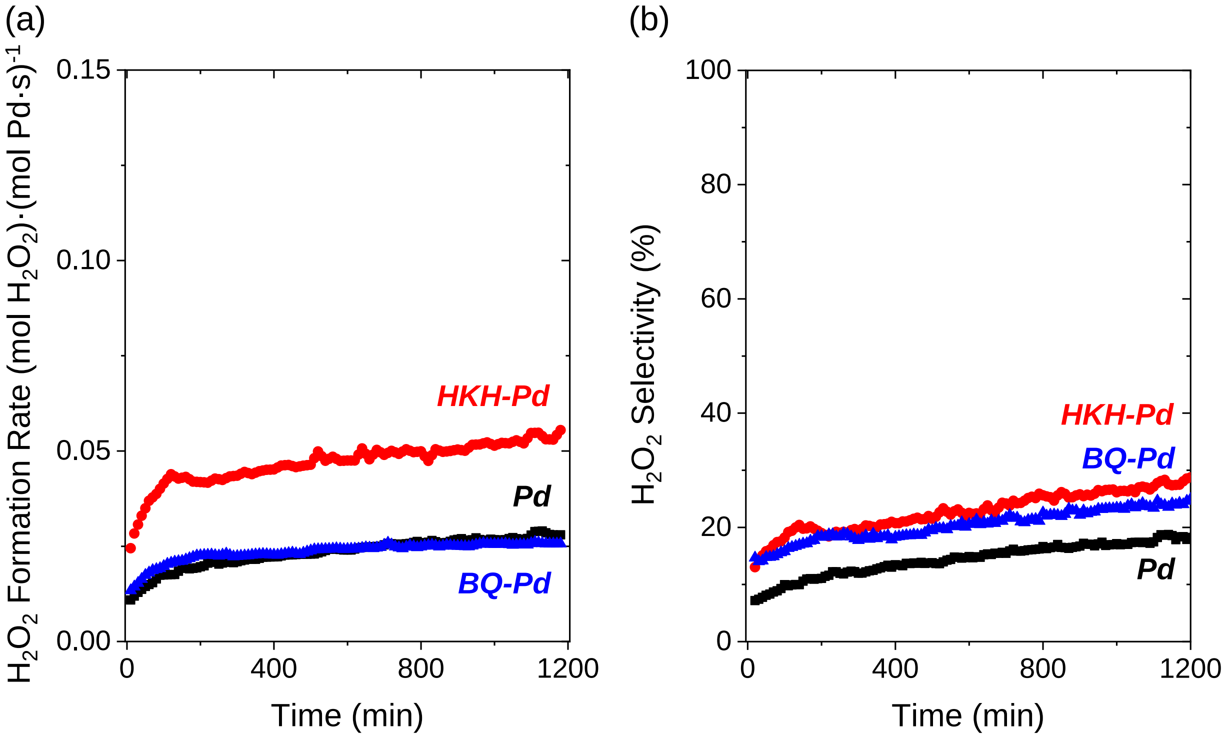

Figure. S6. (a) Rates and (b) selectivities of H_2O_2 formation as a function of time over SiO_2-supported Pd nanoparticles without treatment (black ■) and following treatment with 1,4-benzoquinone (blue ▲) or hexaketocyclohexane (red ●). Measurements used DI H_2O as the solvent within a fixed-bed reactor (60 kPa H_2, 100 kPa O_2, 278 K). Pd samples were prepared using strong electrostatic adsorption of $(NH_4)_2PdCl_4$ on SiO_2 as the catalyst precursor.

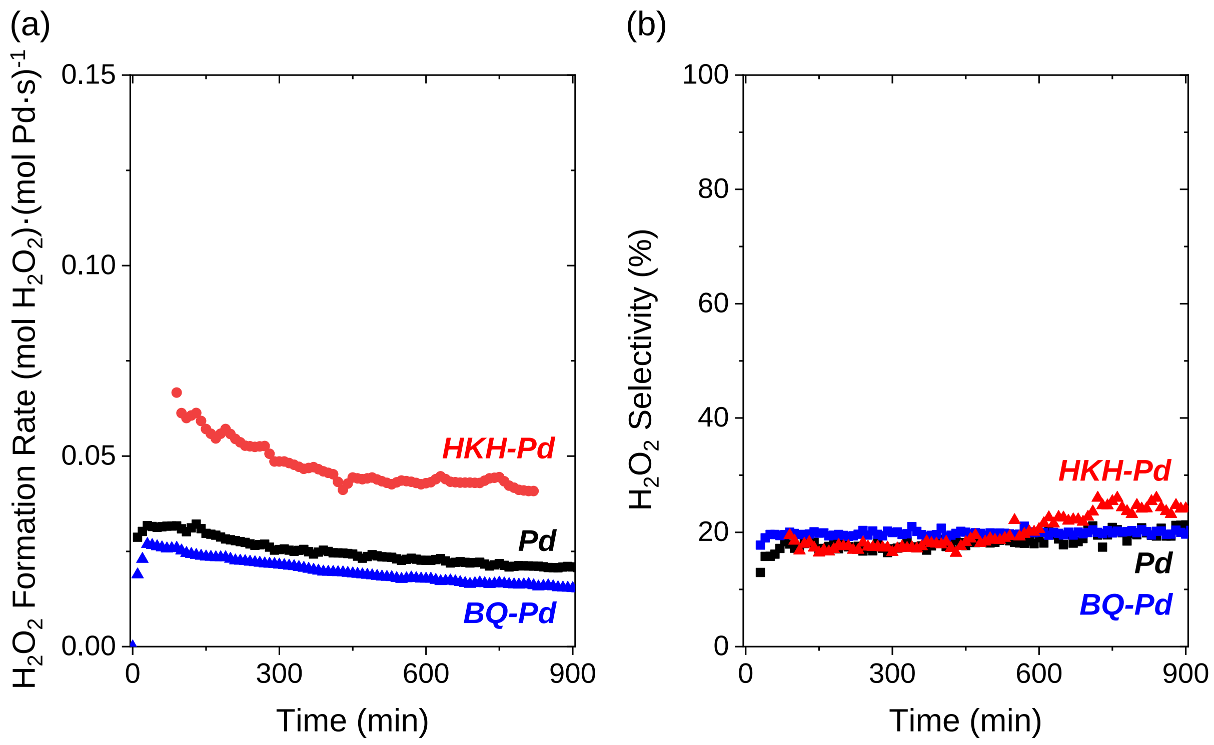

Figure. S7. (a) Rates and (b) selectivities of H_2O_2 formation as a function of time over SiO_2-supported Pd nanoparticles without treatment (black ■) and following treatment with 1,4-benzoquinone (blue ▲) or hexaketocyclohexane (red ●). Measurements used DI H_2O as the solvent within a fixed-bed reactor (60 kPa H_2, 100 kPa O_2, 278 K). Pd samples were prepared using strong electrostatic adsorption of $Pd(NO_3)_2$ on SiO_2 as the catalyst precursor.

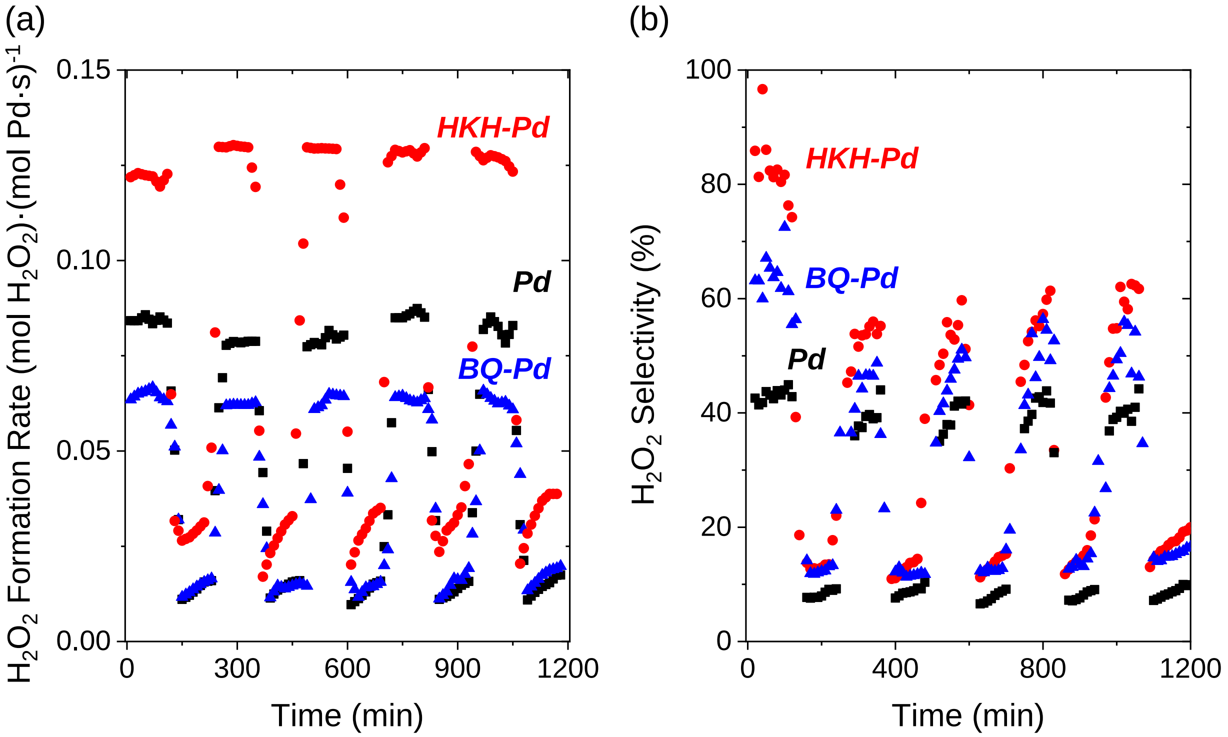

Figure. S8. (a) Rates and (b) selectivities of H_2O_2 formation as a function of time over SiO_2-supported Pd nanoparticles without treatment (black ■) and following treatment with 1,4-benzoquinone (blue ▲) or hexaketocyclohexane (red ●). Measurements used DI H_2O as the solvent within a fixed-bed reactor cycling between high pressures of H_2 (200 kPa H_2, 60 kPa O_2, 278 K) and O_2 (60 kPa H_2, 100 kPa O_2, 278 K) for 2 h periods. Pd samples were prepared using strong electrostatic adsorption of $(NH_4)_2PdCl_4$ on SiO_2 as the catalyst precursor.

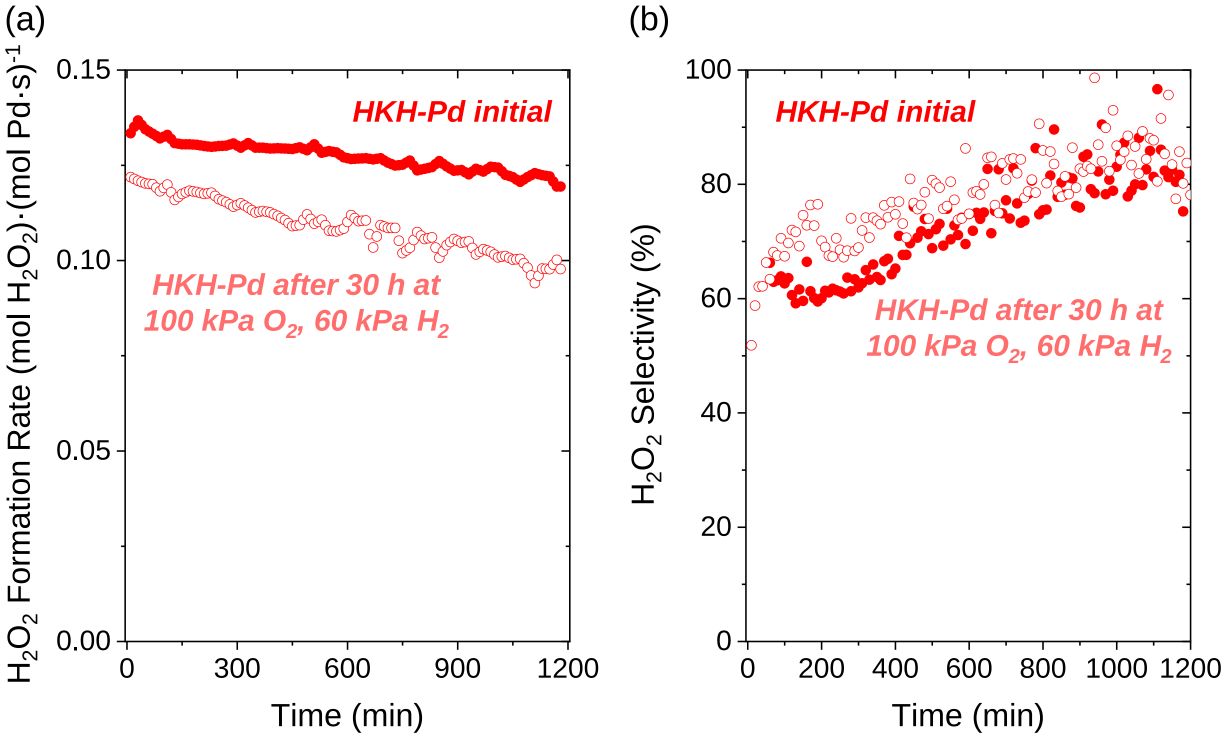

Figure. S9. (a) Rates and (b) selectivities of H_2O_2 formation as a function of time over SiO_2-supported Pd nanoparticles following treatment with hexaketocyclohexane (red ●) and on the same material after operating at high pressures of O_2 for 30 h (60 kPa H_2, 100 kPa O_2, 278 K; red ◌). Measurements used DI H_2O as the solvent within a fixed-bed reactor (200 kPa H_2, 60 kPa O_2, 278 K). Pd samples were prepared using strong electrostatic adsorption of $(NH_4)_2PdCl_4$ on SiO_2 as the catalyst precursor.

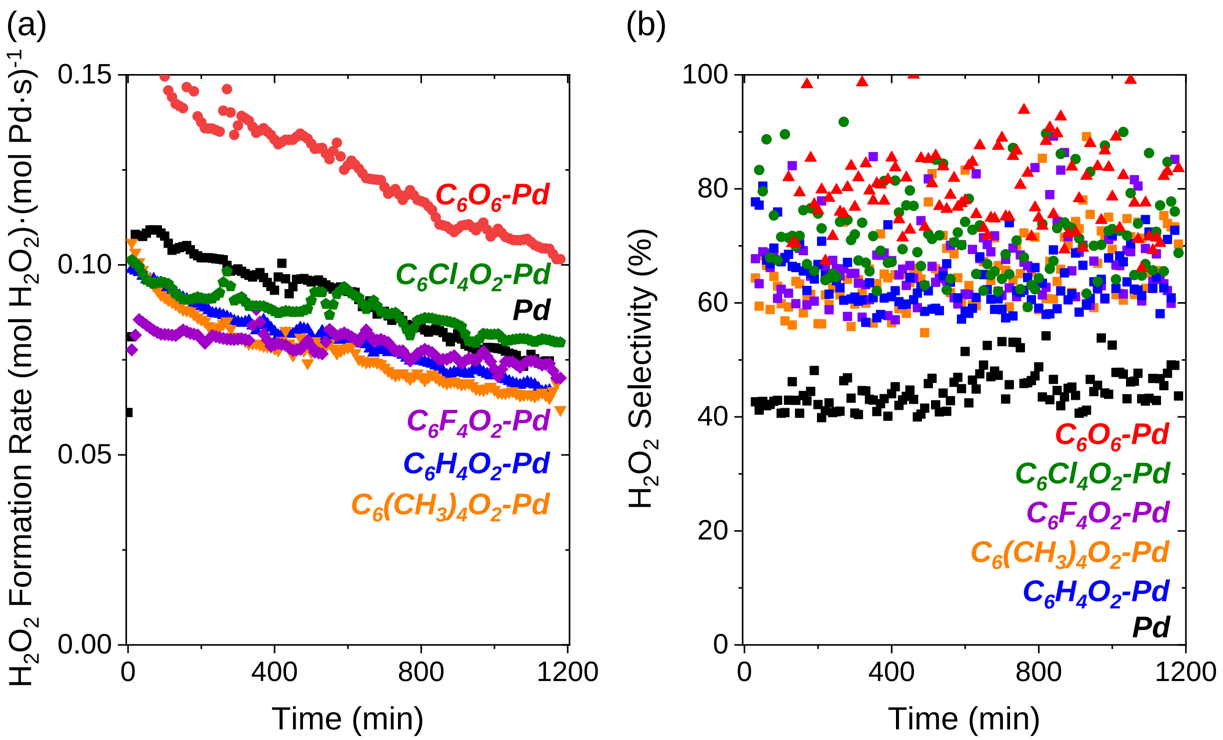

Figure S10. (a) Rates and (b) selectivities of H_2O_2 formation as a function of time over SiO_2-supported Pd nanoparticles without treatment (black ■) and following treatment with 1,4-benzoquinone (blue ▲), Duroquinone (orange ▼), tetrafluoro-1,4-benzoquinone (purple ♦), tetrachloro-1,4-benzoquinone (green ⬟), or hexaketocyclohexane (red ●). Measurements used DI H_2O as the solvent within a fixed-bed reactor (200 kPa H_2, 60 kPa O_2, 278 K). Pd samples were prepared using strong electrostatic adsorption of $Pd(NO_3)_2$ on SiO_2 as the catalyst precursor.

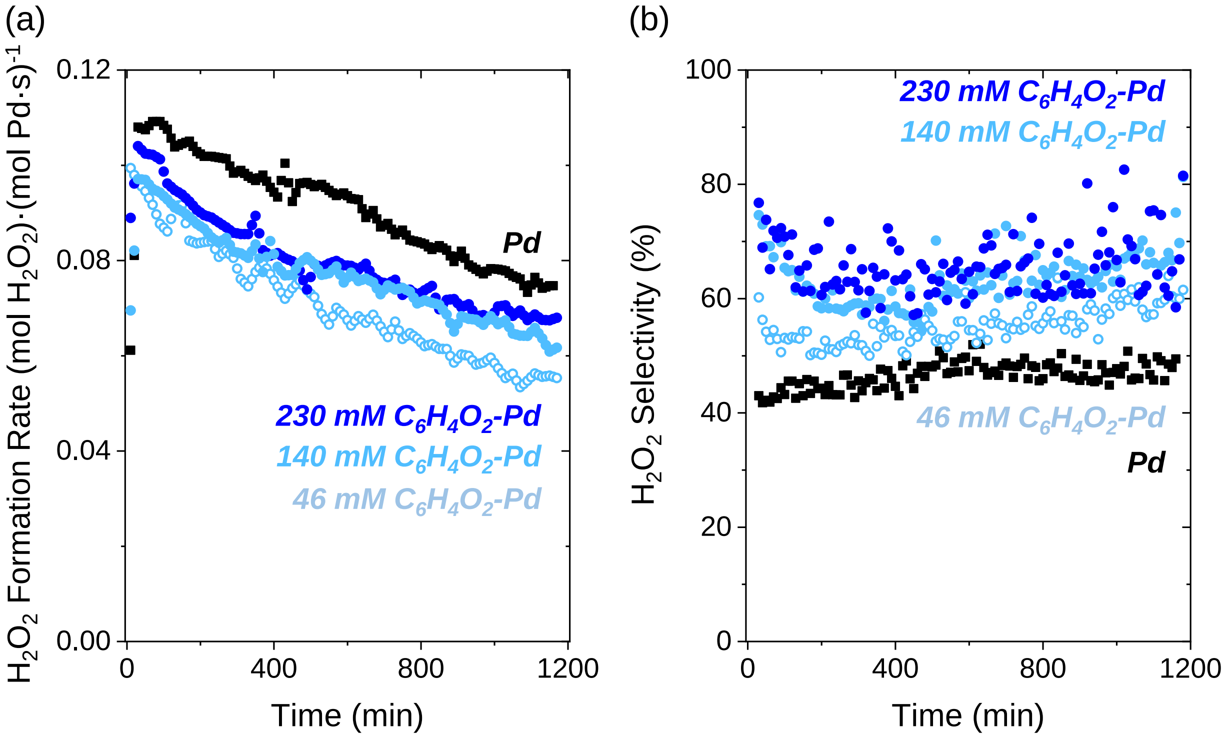

Figure S11. (a) Rates and (b) selectivities of H_2O_2 formation as a function of time over SiO_2-supported Pd nanoparticles without treatment (black ■) and following treatment with 46 mM (light blue ◌), 140 mM (light blue ●),), and 230 mM (dark blue ●) concentrations of 1,4-benzoquinone. Pd samples were prepared using strong electrostatic adsorption of $Pd(NO_3)_2$ on SiO_2 as the catalyst precursor.

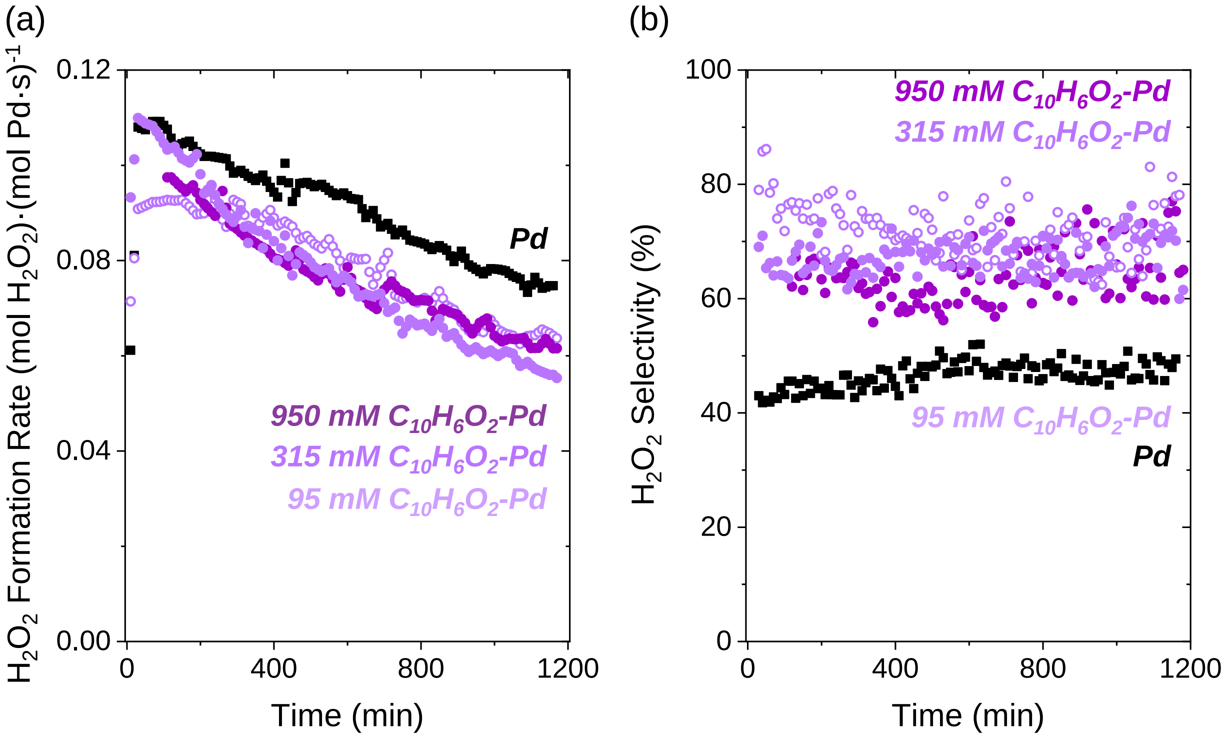

Figure S12. (a) Rates and (b) selectivities of H_2O_2 formation as a function of time over SiO_2-supported Pd nanoparticles without treatment (black ■) and following treatment with 95 mM (light purple ○), 315 mM (light purple ●),), and 950 mM (dark purple ●) concentrations of 1,4-naphthoquinone. Pd samples were prepared using strong electrostatic adsorption of $Pd(NO_3)_2$ on SiO_2 as the catalyst precursor.

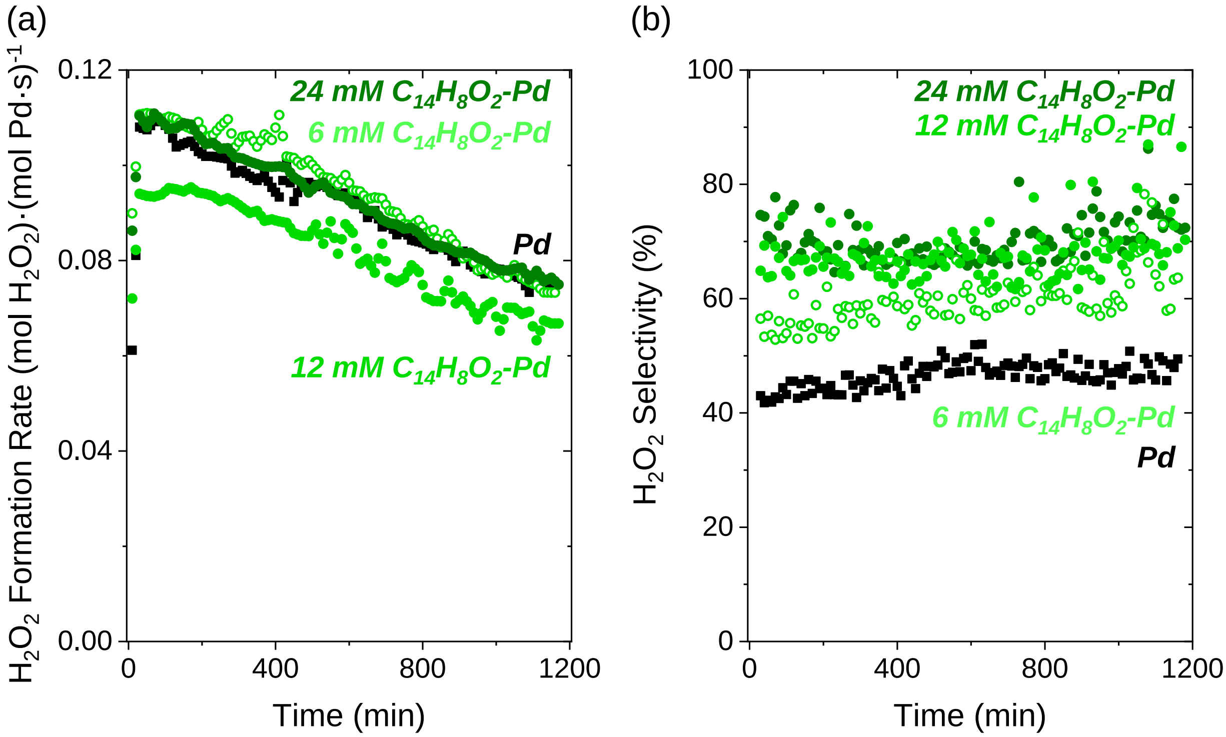

Figure S13. (a) Rates and (b) selectivities of H_2O_2 formation as a function of time over SiO_2-supported Pd nanoparticles without treatment (black ■) and following treatment with 6 mM (light green ○), 12 mM (light green ●),), and 24 mM (dark green ●) concentrations of 1,4-anthraquinone. Pd samples were prepared using strong electrostatic adsorption of $Pd(NO_3)_2$ on SiO_2 as the catalyst precursor.

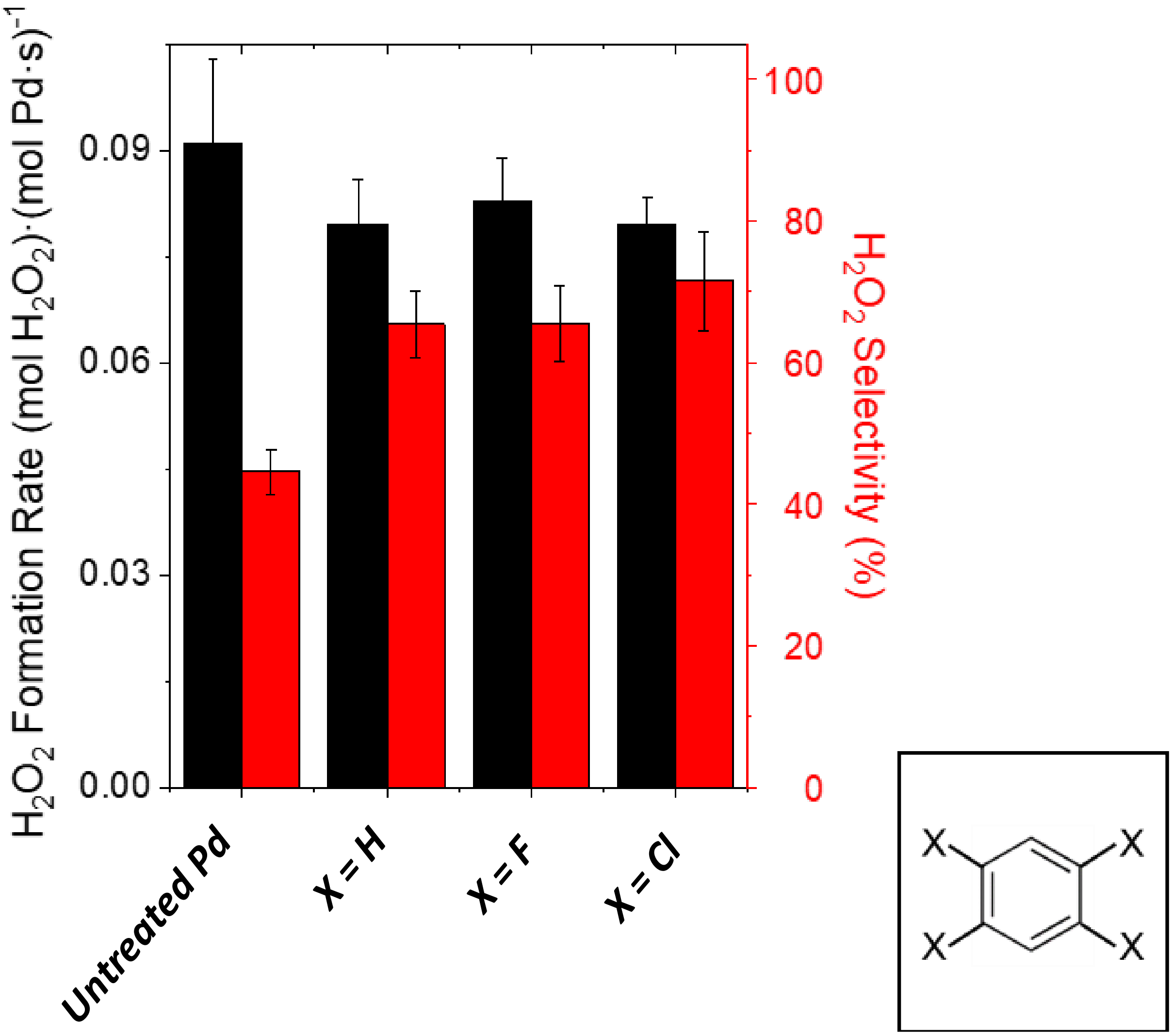

Figure S14. Average rates (black) and selectivities (red) of H_2O_2 formation over SiO_2-supported Pd nanoparticles without treatment and following treatment with analogs of benzene ($C_6X_4H_2$) with different functional groups (X = H, F, Cl). Measurements used DI H_2O as the solvent within a fixed-bed reactor (200 kPa H_2, 60 kPa O_2, 278 K). Figure S8 shows the corresponding time-on-stream measurements. Pd samples were prepared using strong electrostatic adsorption of $Pd(NO_3)_2$ on SiO_2 as the catalyst precursor.

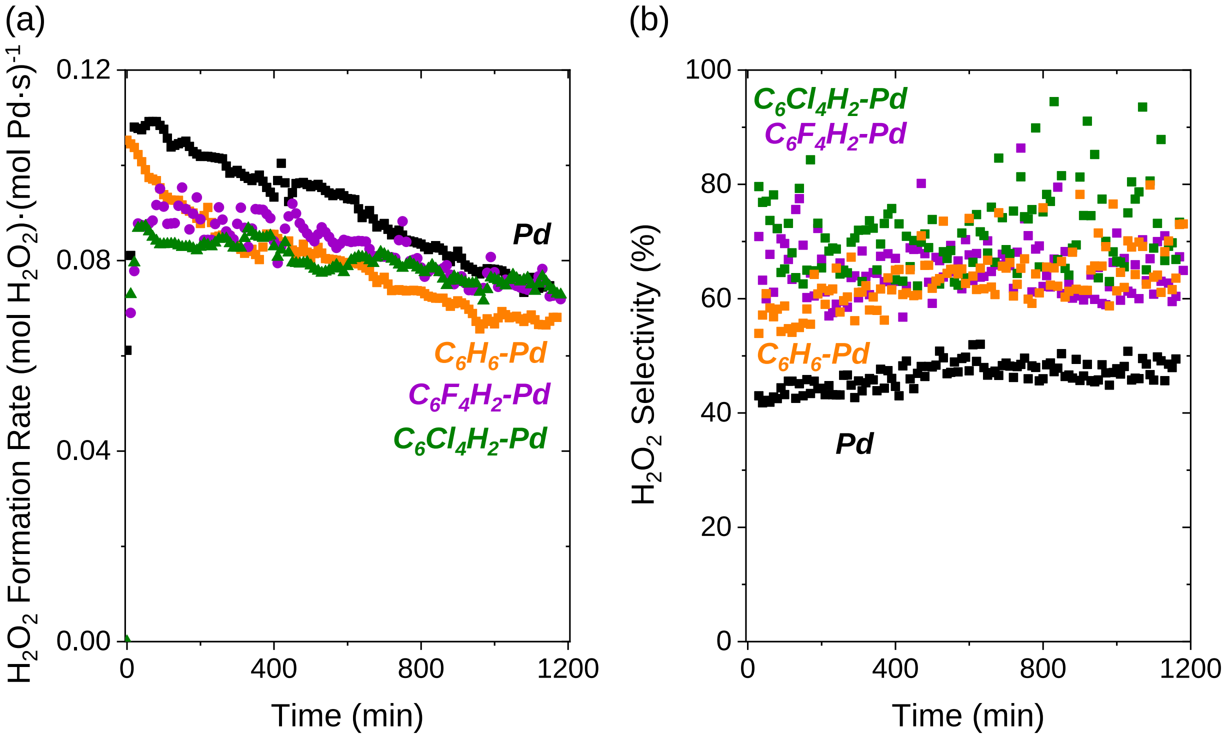

Figure S15. (a) Rates and (b) selectivities of H_2O_2 formation as a function of time over SiO_2-supported Pd nanoparticles without treatment (black ■) and following treatment with benzene (orange ▼), 1,2,4,5-tetrafluorobenzene (purple ♦), or 1,2,4,5-tetrachlorobenzene (green ⬟). Measurements used DI H_2O as the solvent within a fixed-bed reactor (200 kPa H_2, 60 kPa O_2, 278 K). Pd samples were prepared using strong electrostatic adsorption of $Pd(NO_3)_2$ on SiO_2 as the catalyst precursor.

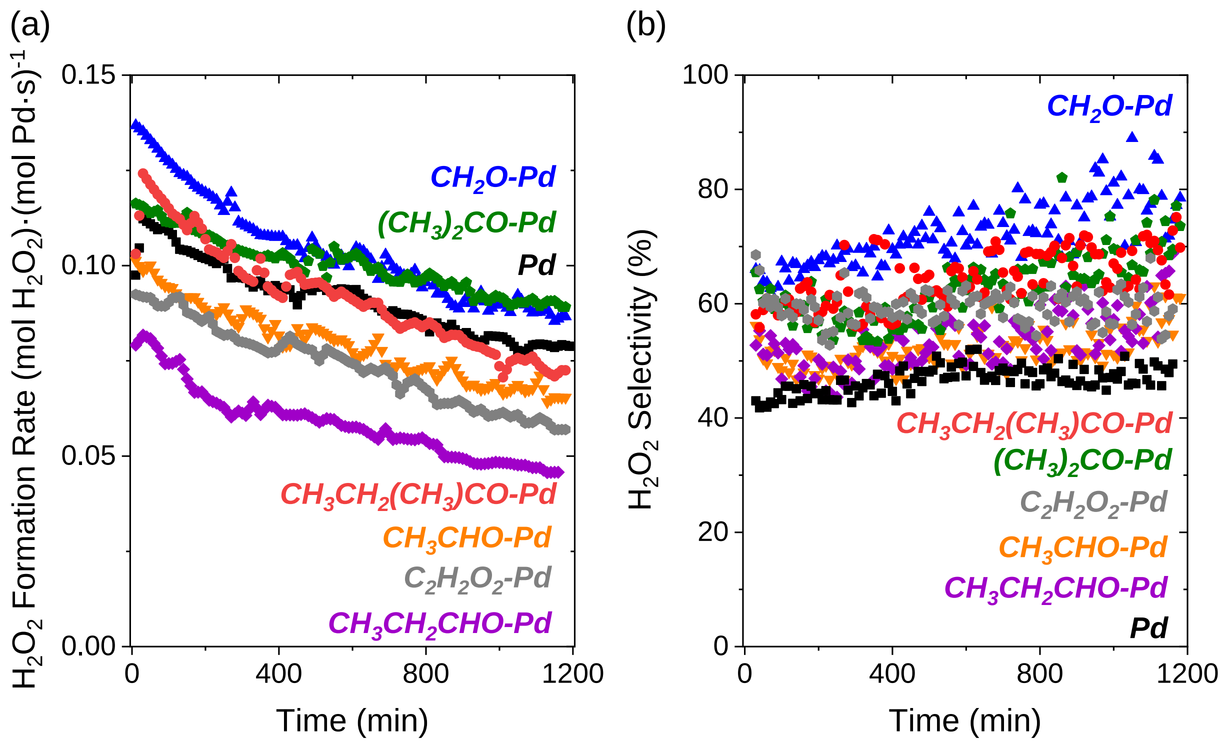

Figure S16. (a) Rates and (b) selectivities of H_2O_2 formation as a function of time over SiO_2-supported Pd nanoparticles without treatment (black ■) and following treatment with formalin (blue ▲), acetaldehyde (orange ▼), propionaldehyde (purple ♦), acetone (green ⬟), 2-butanone (red ●), or glyoxal (gray ⬢). Measurements used DI H_2O as the solvent within a fixed-bed reactor (200 kPa H_2, 60 kPa O_2, 278 K). Pd samples were prepared using strong electrostatic adsorption of $Pd(NO_3)_2$ on SiO_2 as the catalyst precursor.

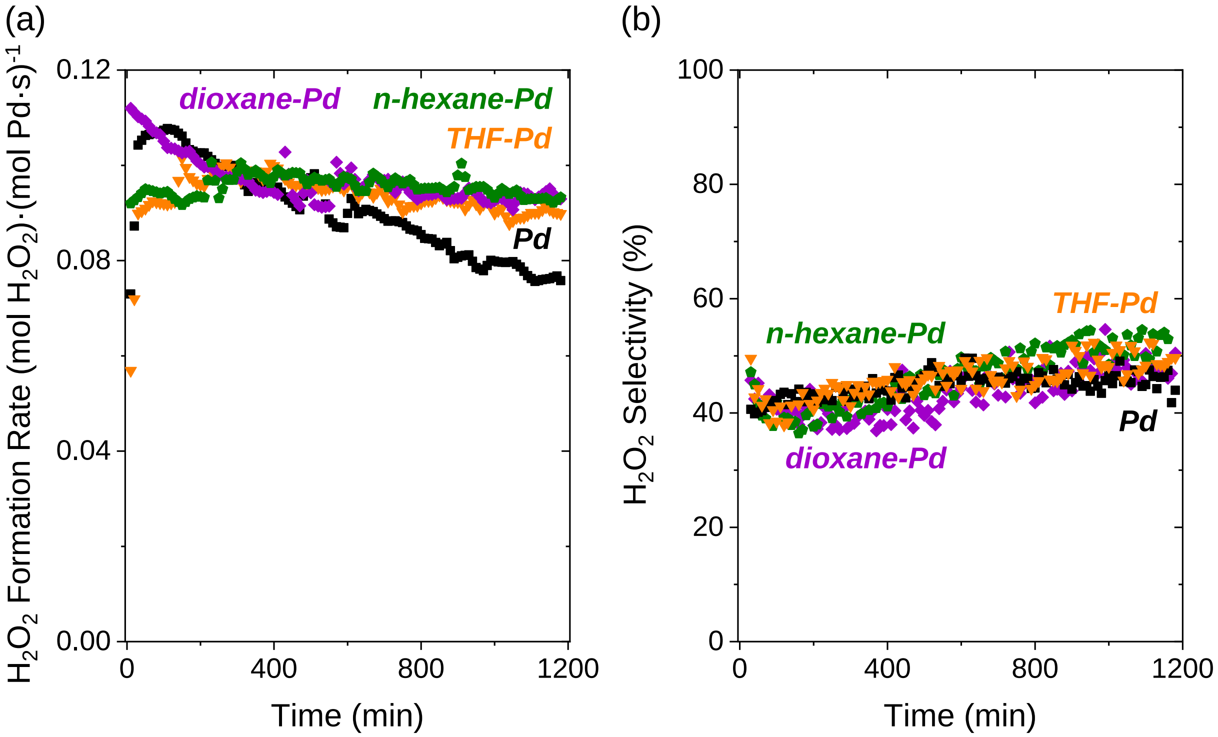

Figure S17. (a) Rates and (b) selectivities of H_2O_2 formation as a function of time over SiO_2-supported Pd nanoparticles without treatment (black ■) and following treatment with tetrahydrofuran (orange ▼), dioxane (purple ♦), or n-hexane (green ⬟). Measurements used DI H_2O as the solvent within a fixed-bed reactor (200 kPa H_2, 60 kPa O_2, 278 K). Pd samples were prepared using strong electrostatic adsorption of $Pd(NO_3)_2$ on SiO_2 as the catalyst precursor.

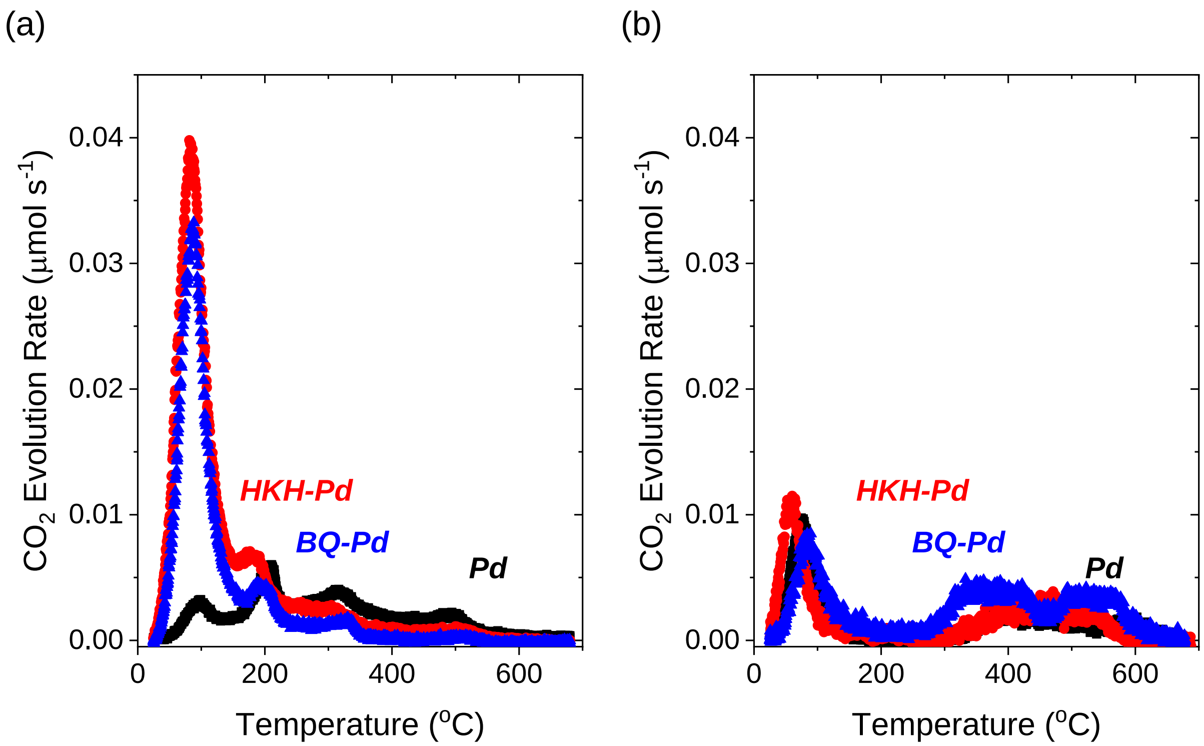

Figure S18. (a) Temperature programmed oxidation (5 kPa O_2, 96 kPa N_2) and (b) temperature programmed desorption (101 kPa He) profiles of SiO_2-supported Pd nanoparticles without treatment (black ■) and following treatment with 1,4-benzoquinone (blue ▲) or hexaketocyclohexane (red ●) after catalysis within a fixed-bed reactor (200 kPa H_2, 60 kPa O_2, 278 K) for 20 h. CO_2 evolution was measured as a function of temperature within a quartz tube furnace (5 K min^{-1}, 100 cm^3 min^{-1}, 298-973 K). Pd samples were prepared using strong electrostatic adsorption of $(NH_4)_2PdCl_4$ on SiO_2 as the catalyst precursor.

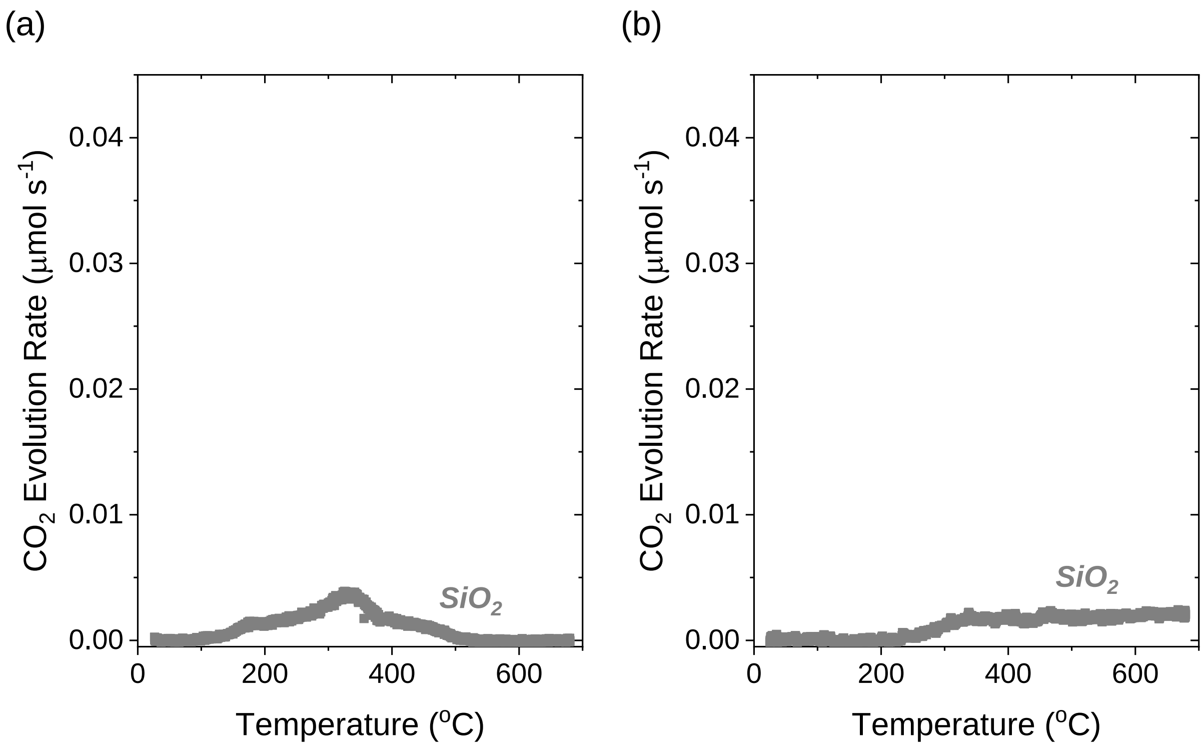

Figure S19. (a) Temperature programmed oxidation (5 kPa O_2, 96 kPa N_2) and (b) temperature programmed desorption (101 kPa He) profiles of the SiO_2 support. CO_2 evolution was measured as a function of temperature within a quartz tube furnace (5 K min^{-1}, 5 kPa O_2, 96 kPa N_2, 100 cm^3 min^{-1}, 298-973 K).

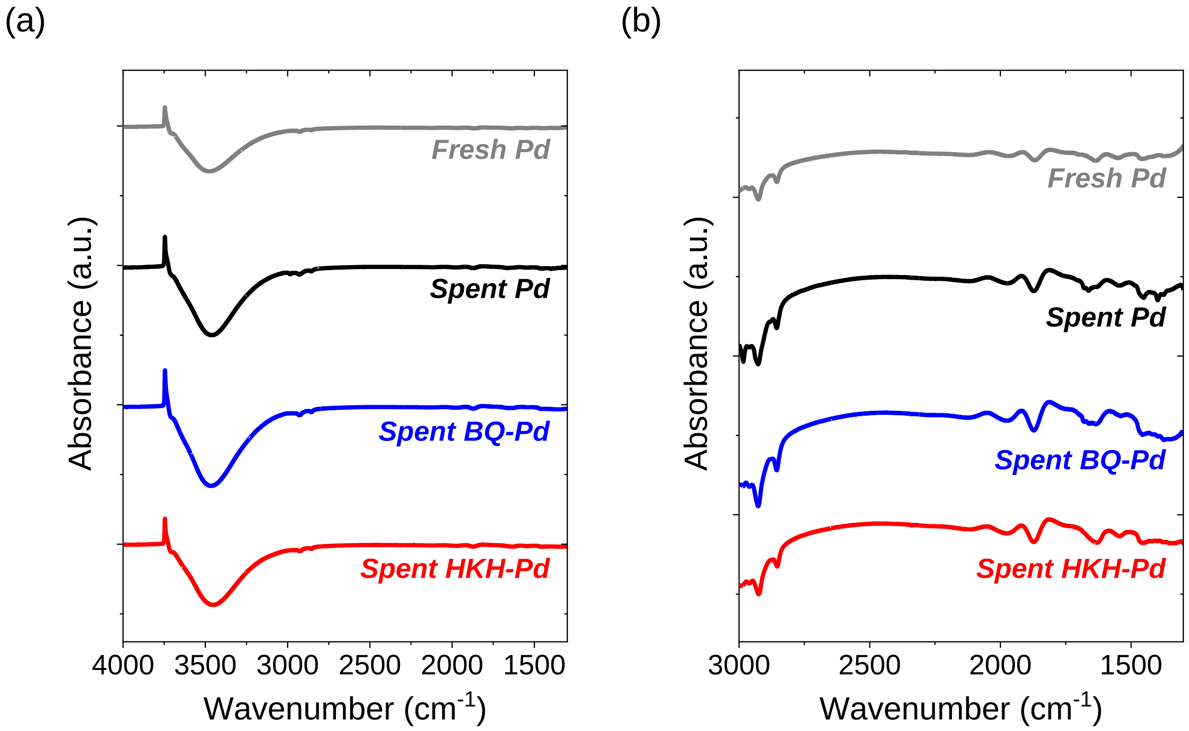

Figure S20. Difference of infrared spectra of SiO_2-supported Pd nanoparticles **(a)** following initial reduction (20 kPa H_2, 373 K) for 1 h and **(b)** after subsequent oxidation (20 kPa O_2, 573 K) and reduction (20 kPa H_2, 573 K) for 1 h each. Samples were freshly prepared (gray) or extracted after catalysis within a fixed-bed reactor (200 kPa H_2, 60 kPa O_2, 278 K) for 20 h without treatment (black) and following treatment with 1,4-benzoquinone (blue) or hexaketocyclohexane (red). Pd samples were prepared using strong electrostatic adsorption of $(NH_4)_2PdCl_4$ on SiO_2 as the catalyst precursor.

Table S2. Ratio of integrated peak area of bridging CO (A_{bridge}; 1800-2015 cm^{-1}) versus atop CO (A_{atop}; 2015-2200 cm^{-1}) calculated from infrared spectra of CO on Pd, as shown in Figure 6b. Spectra were collected on SiO_2-supported Pd nanoparticles (0.02 kPa CO, 303 K) freshly prepared and after catalysis within a fixed-bed reactor (200 kPa H_2, 60 kPa O_2, 278 K) for 20 h without treatment and following treatment with 1,4-benzoquinone or hexaketocyclohexane. Spectra were collected following initial reduction (20 kPa H_2, 373 K) for 1 h (dotted lines) and subsequent oxidation (20 kPa O_2, 573 K) and reduction (20 kPa H_2, 573 K) for 1 h each (solid lines). Pd samples were prepared using strong electrostatic adsorption of $(NH_4)_2PdCl_4$ on SiO_2 as the catalyst precursor.

Sample	A_{bridge}/A_{atop}	
	After Initial Reduction at 373 K	After Subsequent Oxidation and Reduction at 573 K
Fresh Untreated Pd-SiO_2	1.00	0.71
Spent Untreated Pd-SiO_2	3.22	2.52
Spent BQ-treated Pd-SiO_2	4.16	2.66
Spent HKH-treated Pd-SiO_2	3.08	2.36

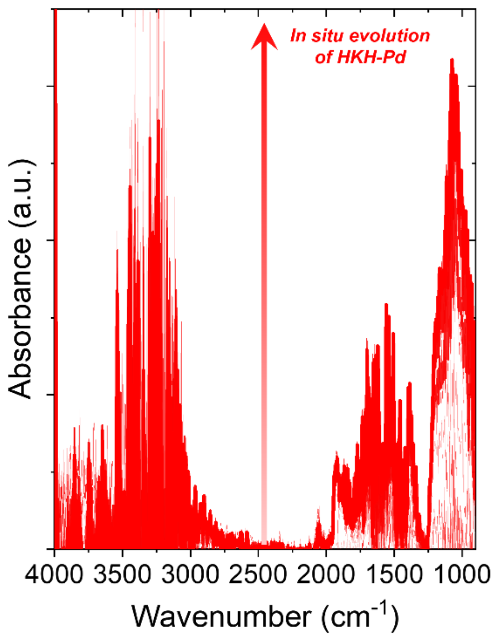

Figure S21. *In situ* infrared spectra of 5 mM hexaketocyclohexane accumulating on SiO_2-supported Pd nanoparticles (10 wt% Pd, 75 kPa H_2, 25 kPa O_2, 298K) over the course of 5 hours using DI H_2O as the solvent with dissolved HKH present.

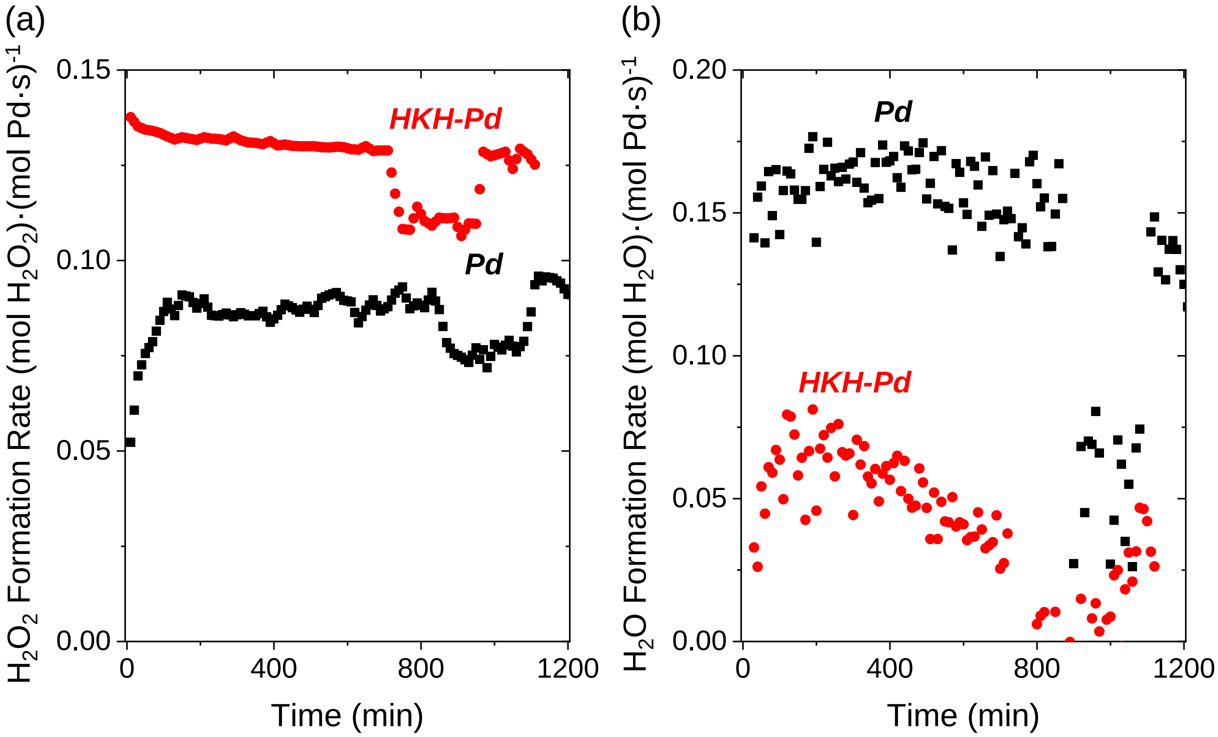

Figure S22. Rates of (a) H_2O_2 and (b) H_2O formation as a function of time over SiO_2-supported Pd nanoparticles without treatment (black ■) and following treatment with hexaketocyclohexane (red ●). Measurements used DI H_2O as the solvent and H_2 or D_2 as the reductant within a fixed-bed reactor (200 kPa H_2 or D_2, 60 kPa O_2, 278 K). The discontinuous decrease in reaction rates after 600 min coincides with switching the reductant from H_2 to D_2. Pd samples were prepared using strong electrostatic adsorption of $(NH_4)_2PdCl_4$ on SiO_2 as the catalyst precursor.

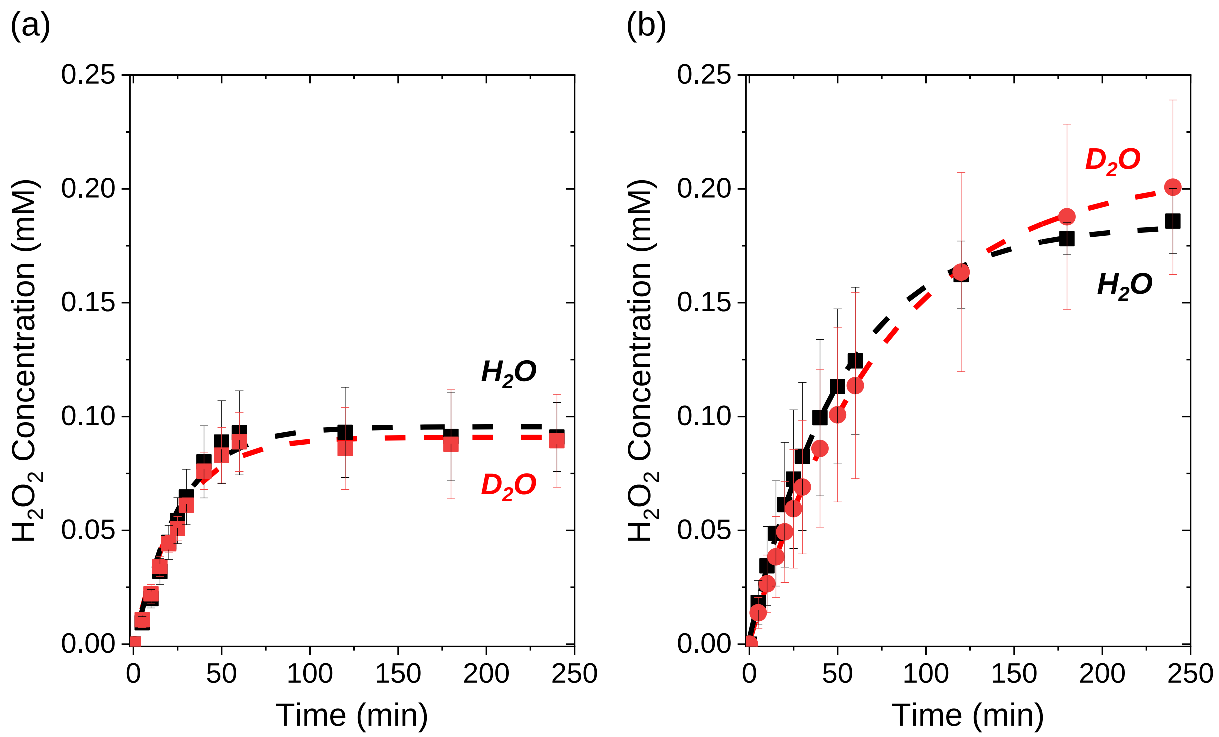

Figure S23. The concentration of H_2O_2 formed as a function of time over SiO_2-supported Pd nanoparticles (a) without treatment and (b) following treatment with hexaketocyclohexane when reacting H_2 within H_2O (black ■) or D_2O (red ●) as solvents (4.8 kPa H_2, 4.8 kPa O_2, 80 cm^3 solvent, 50 mg Pd-SiO_2, 298 K). Dashed lines fitted to equations using methods reported in reference 55 of the main text. Pd samples were prepared using strong electrostatic adsorption of $Pd(NO_3)_2$ on SiO_2 as the catalyst precursor.

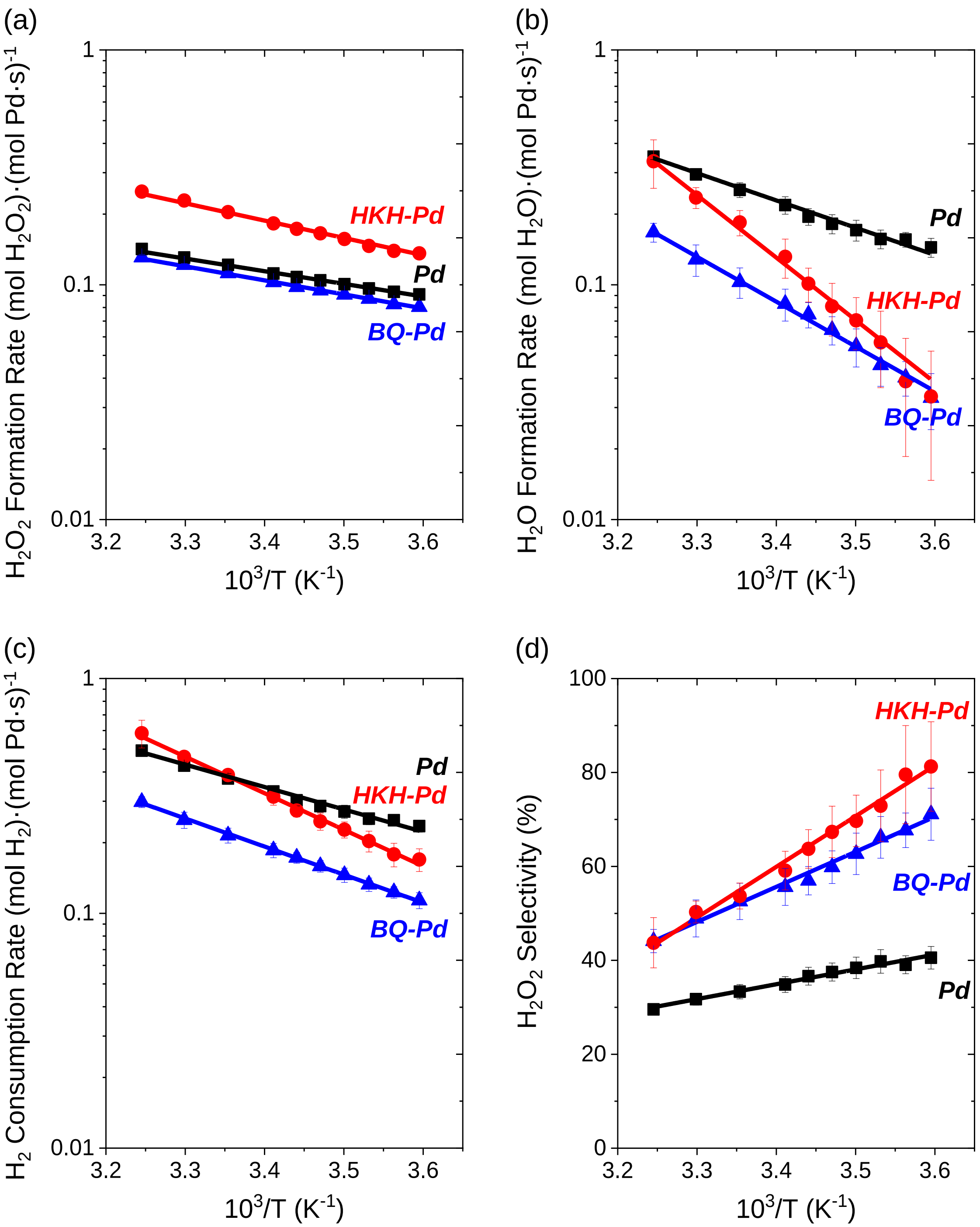

Figure S24. Steady-state rates of (a) H_2O_2 formation, (b) H_2O formation, (c) H_2 consumption, and (d) H_2O_2 selectivity as a function of inverse temperature over SiO_2-supported Pd nanoparticles without treatment (black ■) and following treatment with 1,4-benzoquinone (blue ▲) or hexaketocyclohexane (red ●). Measurements used DI H_2O as the solvent within a fixed-bed reactor (200 kPa H_2, 60 kPa O_2, 278-308 K). Linear fits give apparent activation enthalpies in Table 2. Pd samples were prepared using strong electrostatic adsorption of $Pd(NO_3)_2$ on SiO_2 as the catalyst precursor.

Computational Analysis

1. Modelled Pd (111) Surfaces

Periodic density functional theory calculations were carried out on the Pd (111) surface to model the experimentally observed reactivity and selectivity differences with various quinone mediators. The Pd (111) surface shown in Figure S25 was used to model the experimentally employed silica supported Pd nanoparticles. The Pd (111) surface was covered with atomically absorbed O* oxygen atoms (5/16 monolayer (ML)) and sub-surface H_s* hydrogen atoms (5/16 ML) to mimic the in-operando nature of the surface, as shown in previous experimental and computational studies.[1] Surface hydrogen was taken as a proton and an electron source. The organic mediators form strong covalent surface bonds with the Pd atoms upon adsorption due to an initial electron transfer to the mediators upon chemisorption, thus blocking surface sites.

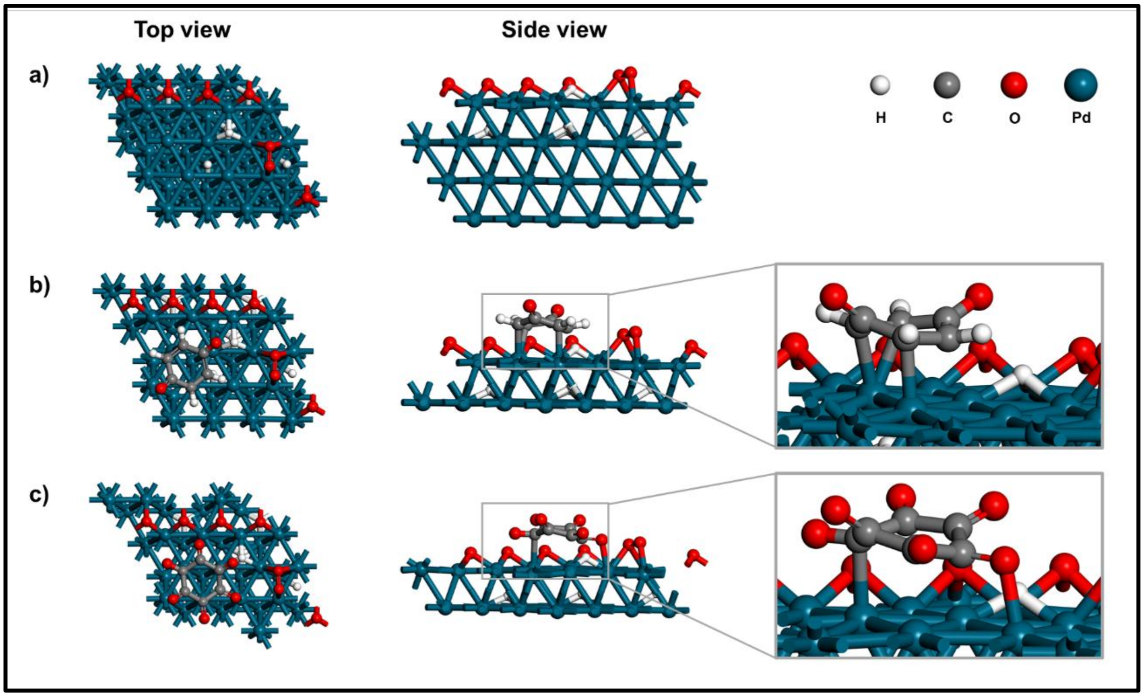

Figure S25. Model Pd (111) surface with atomically absorbed O* oxygen atoms (5/16 ML), O_2* oxygen species (1/16 ML), H* hydrogen atom (1/16 ML), and sub-surface H_s* hydrogen atoms (5/16 ML). Representative surfaces shown for (a) the surface unoccupied by mediator species, (b) BQ-bound surfaces, and (c) HKH-bound surfaces.

2. Binding Energies of Quinones to Pd Surfaces with Varying Coverages of H_s* and O*

The calculated adsorption energy for HKH on the 5/16 ML O* and 5/16 ML H_s* Pd (111) surface using an implicit water solvent shows that HKK strongly chemisorbs with an adsorption energy of -156 kJ mol^{-1}. This strong chemisorption is consistent with the highly electrophilic nature of HKH established in the subsequent discussions. Increasing the surface coverage of atomically adsorbed oxygen atoms, O*, from 2/16 to 8/16 ML decreases the adsorption energy for all the mediators. The increased O* coverage blocks more surface sites, reducing the covalent interaction between the mediator and the surface. Thus, it is speculated that the mediators interact weakly with the surface with fewer covalent bonds under oxygen-rich conditions. Similarly, decreasing the surface coverage of O* atoms increases the strength of adsorption for all the mediators. Changing the H_s* coverage showed no significant change in the binding energies. Increasing the subsurface H_s* coverage from 2/16 ML to 8/16 ML slightly disfavored the adsorption for electron-withdrawing mediators and slightly favored the adsorption for electron-donating mediators. Figure S26 summarizes the implicit solvent adsorption energies for different mediators at different surface coverage of O* and H_s* atoms. These implicit adsorption energies are reported for a mediator at nearly 10 Å away from the Pd surface which then undergoes chemisorption on the Pd surface as shown in Figure S25. During the adsorption, none of the surface or sub-surface bound O*, O_2*, H*, and H_s* species undergo significant displacement.

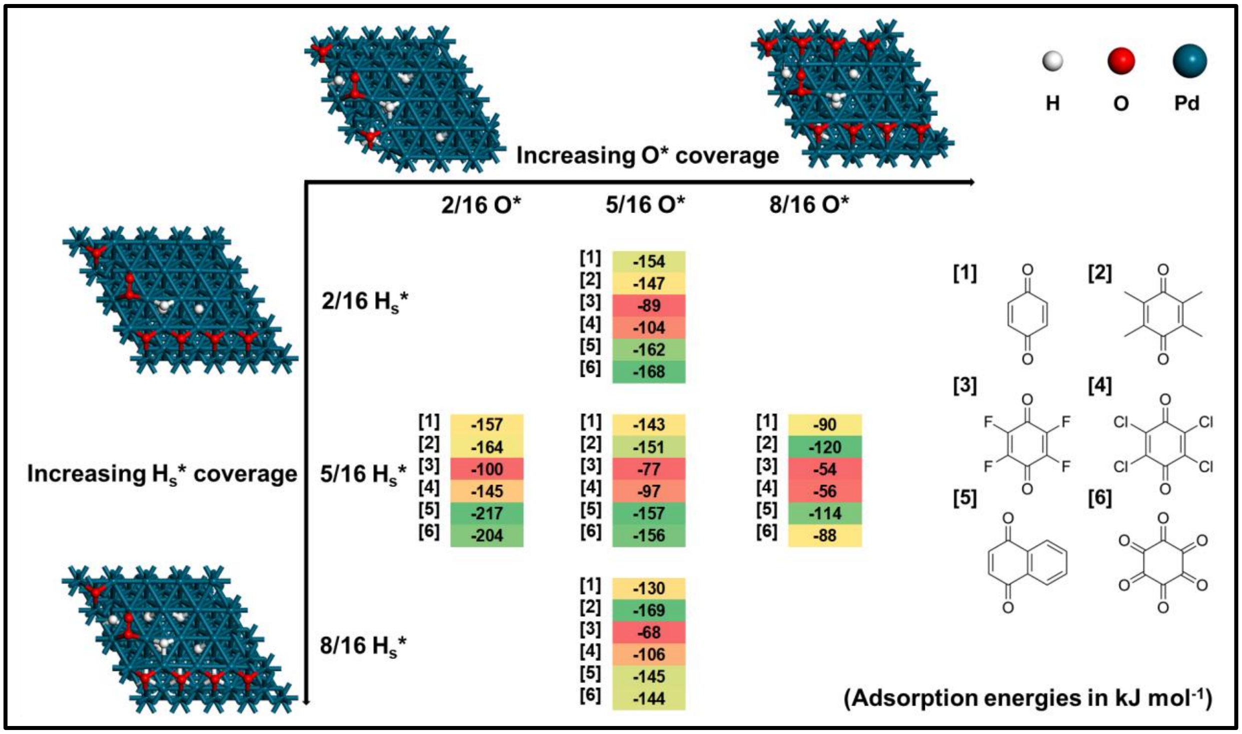

Figure S26. Adsorption energies (in kJ mol^{-1}) of different mediators [1-6] in an implicit solvent as a function of coverages of surface oxygen (O*) (left-to-right) and subsurface hydrogen (H_s*) (top-to-bottom) on Pd (111) surface. Calculations consider the adsorption of [1] 1,4-benzoquinone, [2] tetramethyl-1,4-benzoquinone, [3] tetrafluoro-1,4-benzoquinone, [4] tetrachloro-1,4-benzoquinone, [5] 1,4-napthoquinone, [6] hexaketocyclohexane.

3. Binding of Other Organics to Pd Surfaces with Varying Coverages of H_s* and O*

The adsorption of benzene analogs ($C_6X_4H_2$; X = H, CH_3, F, Cl) to Pd were shown in the main text to enhance the reactivity. As such, we examined the binding energy of these species on Pd (111) surface and their ability to undergo oxidation or hydroxylation to form quinone type intermediates. The surface binding energies of these non-quinone organics were calculated and compared with the quinone species. Figure S27 summarizes the implicit solvent adsorption energies for different non-quinone organics at different surface coverages of O* and H_s* atoms. These calculations show that benzene and its derivatives [1-3] have similar binding energies as the quinone species. The non-aromatic organic additives [4-6], on the other hand, show weaker binding to the Pd surface. The adsorption mode of benzene is similar to that of the quinone derivatives, with the species binding to the Pd (111) surface through the unsaturated C=C or C=O bonds in the rings (Figure S28). The other organic additives such as THF, dioxane, and n-hexane lack these molecular features for surface adsorption, and result in weaker binding to the Pd surface. Increased coverages of surface O* lead to weaker binding of these species, which is similar to the observations of quinone species in Figure S26. The changes in the binding energies with subsurface H_s* coverage are much less pronounced, again consistent with the observations with the quinone species.

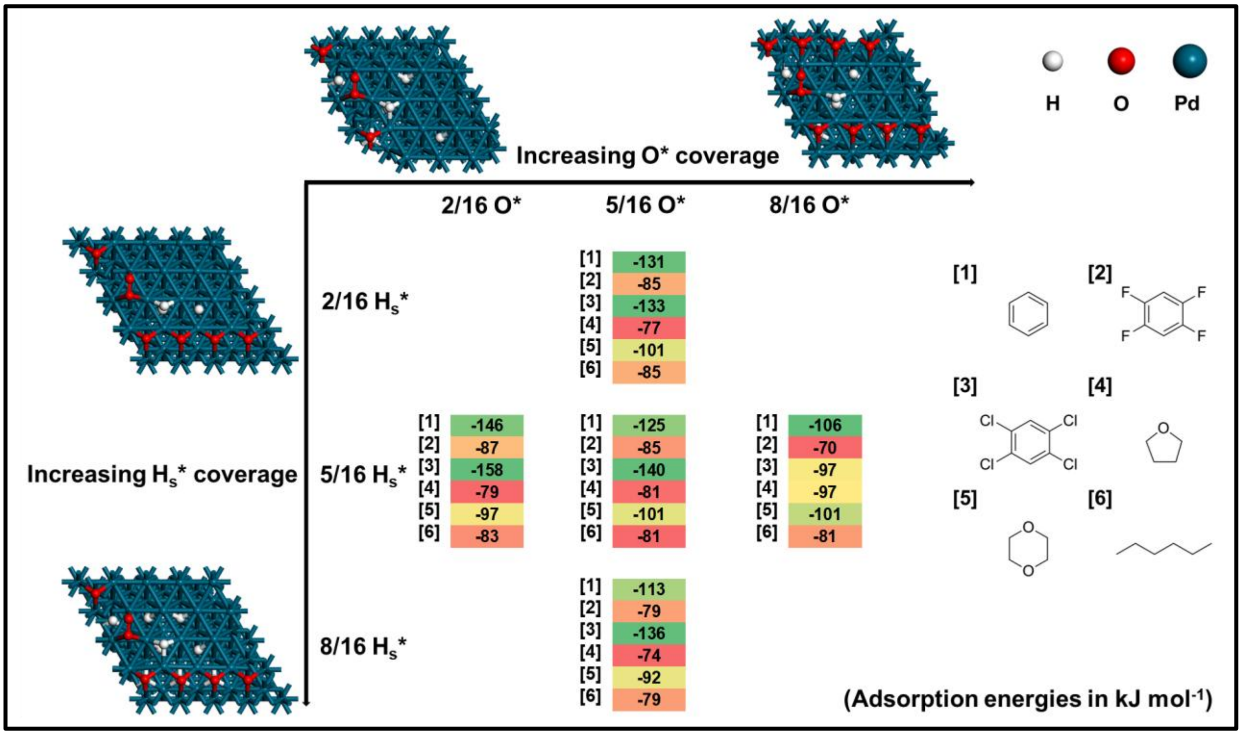

Figure S27. Adsorption energies (in kJ mol^{-1}) of different organics [1-6] in an implicit solvent as a function of coverages of surface oxygen (O*) (left-to-right) and subsurface hydrogen (H_s*) (top-to-bottom) on Pd (111) surface. Calculations consider the adsorption of [1] benzene, [2] 1,2,4,5-tetrafluorobenzene, [3] 1,2,4,5-tetrachlorobenzene, [4] tetrahydrofuran, [5] dioxane, [6] n-hexane.

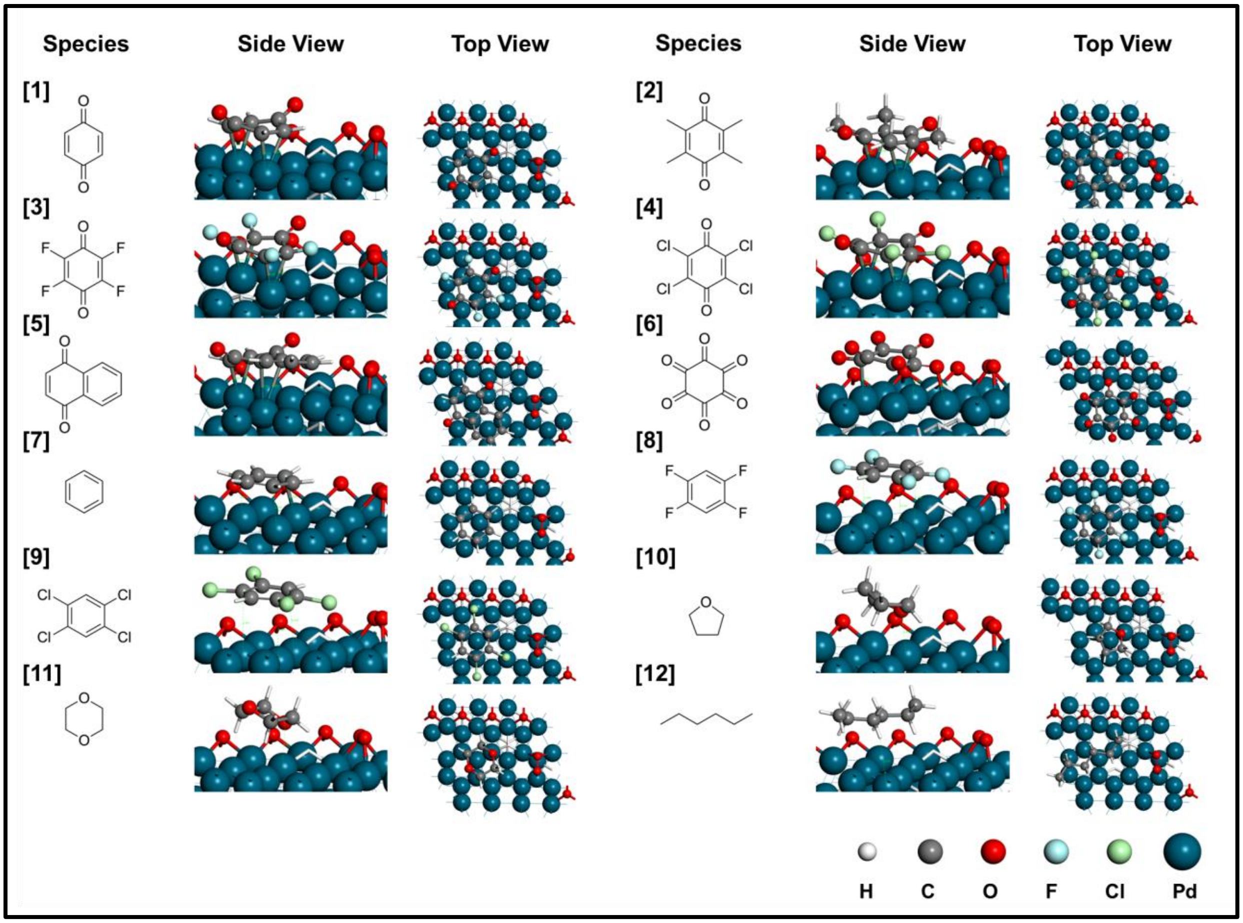

Figure S28. Side and top view of adsorption modes of different organics on the Pd (111) surface. Calculations consider the adsorption of [1] 1,4-benzoquinone, [2] tetramethyl-1,4-benzoquinone, [3] tetrafluoro-1,4-benzoquinone, [4] tetrachloro-1,4-benzoquinone, [5] 1,4-napthoquinone, [6] hexaketocyclohexane, [7] benzene, [8] 1,2,4,5-tetrafluorobenzene, [9] 1,2,4,5-tetrachlorobenzene, [10] tetrahydrofuran, [11] dioxane, [12] n-hexane. The simulated Pd (111) surfaces contain adsorbed O* (5/16 ML) and subsurface H_s* (5/16 ML) to represent the state of Pd NPs at reaction conditions determined by operando EXAFS in our prior work.

4. Surface Oxygenation and Hydroxylation of Benzene

Different possible mechanisms for benzene oxygenation and hydroxylation over the catalytic Pd surfaces were examined to provide insights into the in-situ conversion of aromatics to oxygenated/hydroxylated aromatics. We explicitly examined the direct oxidation via surface oxygen and hydroxylation via surface hydroxyl intermediates for a benzene molecule adsorbed on the model Pd (111) surface with surface O* and subsurface H_s* species. As shown in Figure S29, the oxygenation of benzene via a surface oxygen species to make phenol has a barrier of 120 kJ mol^{-1}. The barrier decreases considerably to 74 kJ mol^{-1} for the direct hydroxylation of benzene via a surface hydroxyl species. We believe that the activation barrier for the water-assisted hydroxylation from surface hydroxyl intermediates or solution phase hydroxyl intermediates formed would further lower barriers and should readily hydroxylate adsorbed aromatics.

The resultant adsorbed hydroxylated aromatic intermediates can act similarly to partially hydrogenated quinone intermediates and facilitate proton-electron transfer pathways that ultimately influence the reaction energetics and selectivity. Similar oxygenated reactive intermediates would be more difficult to obtain with other non-aromatic derivatives such as THF, dioxane, and n-hexane, which helps to explain their ineffectiveness as efficient mediators.

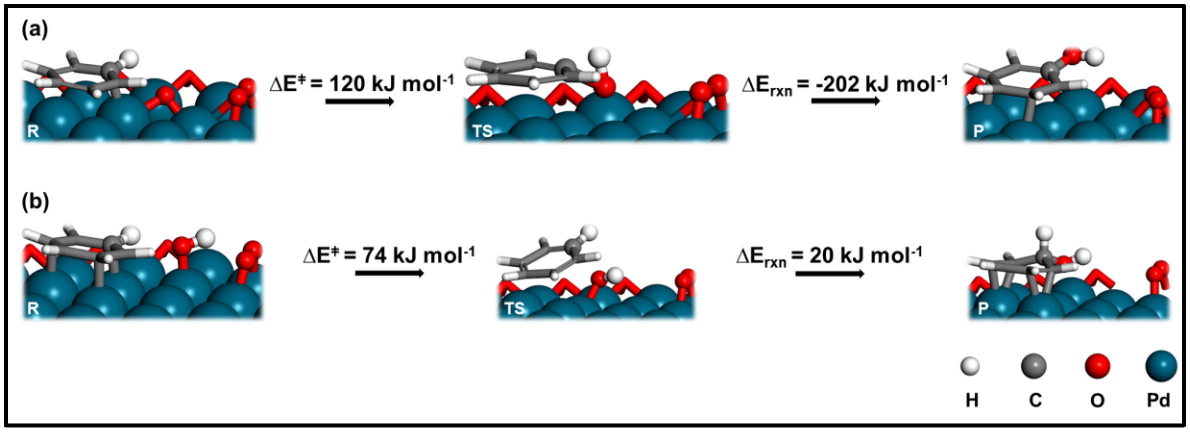

Figure S29. (a) Direct surface oxygenation vs (b) direct surface hydroxylation of benzene in implicit water. Each step shows the corresponding reaction energies (ΔE_{rxn}) and intrinsic barriers ($\Delta E^{\ddagger}$) in kJ mol^{-1}. The simulated Pd (111) surfaces contain adsorbed O* (5/16 ML) and subsurface H_s* (5/16 ML) to represent the state of Pd NPs at reaction conditions determined by operando EXAFS in our prior work.

5. Calculations of Barriers Quinone Hydrogenation and O_2 Reduction on Pd Surfaces

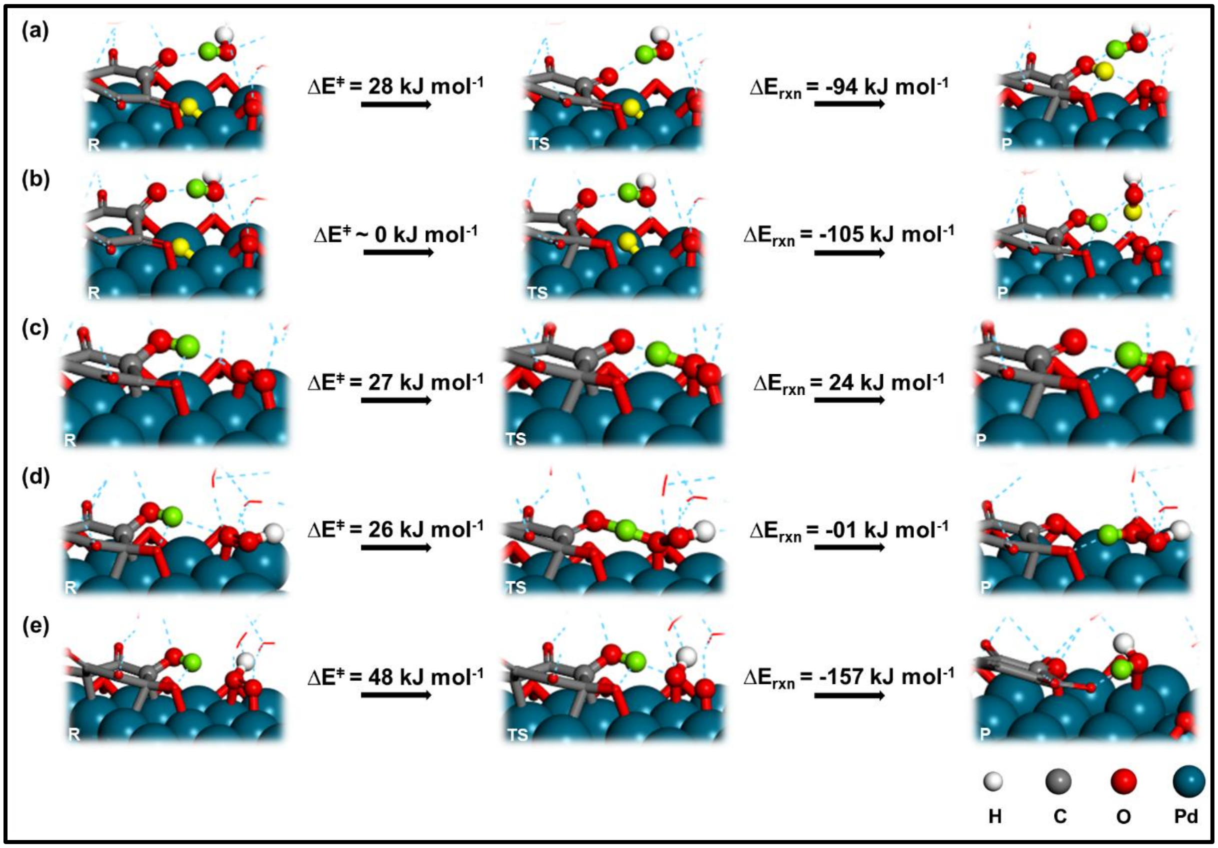

Figure S30. (a) Direct surface hydrogenation vs (b) proton coupled electron transfer step for hydrogenation of the HKH mediator in water. (c) Proton coupled electron transfer step for hydrogenation of O_2* from the hydrogenated HKH mediator. (d) Formation of H_2O_2 vs (e) formation of H_2O from the hydrogenated HKH mediator. Each step shows the corresponding reaction energies (ΔE_{rxn}) and intrinsic barriers ($\Delta E^{\ddagger}$) in kJ mol^{-1}. The simulated Pd (111) surfaces contain adsorbed O* (5/16 ML) and subsurface H_s* (5/16 ML) to represent the state of Pd NPs at reaction conditions determined by operando EXAFS in our prior work. The yellow hydrogen atoms are oxidized into a proton, and the green protons are transferred via a water molecule in the heterolytic mechanism.

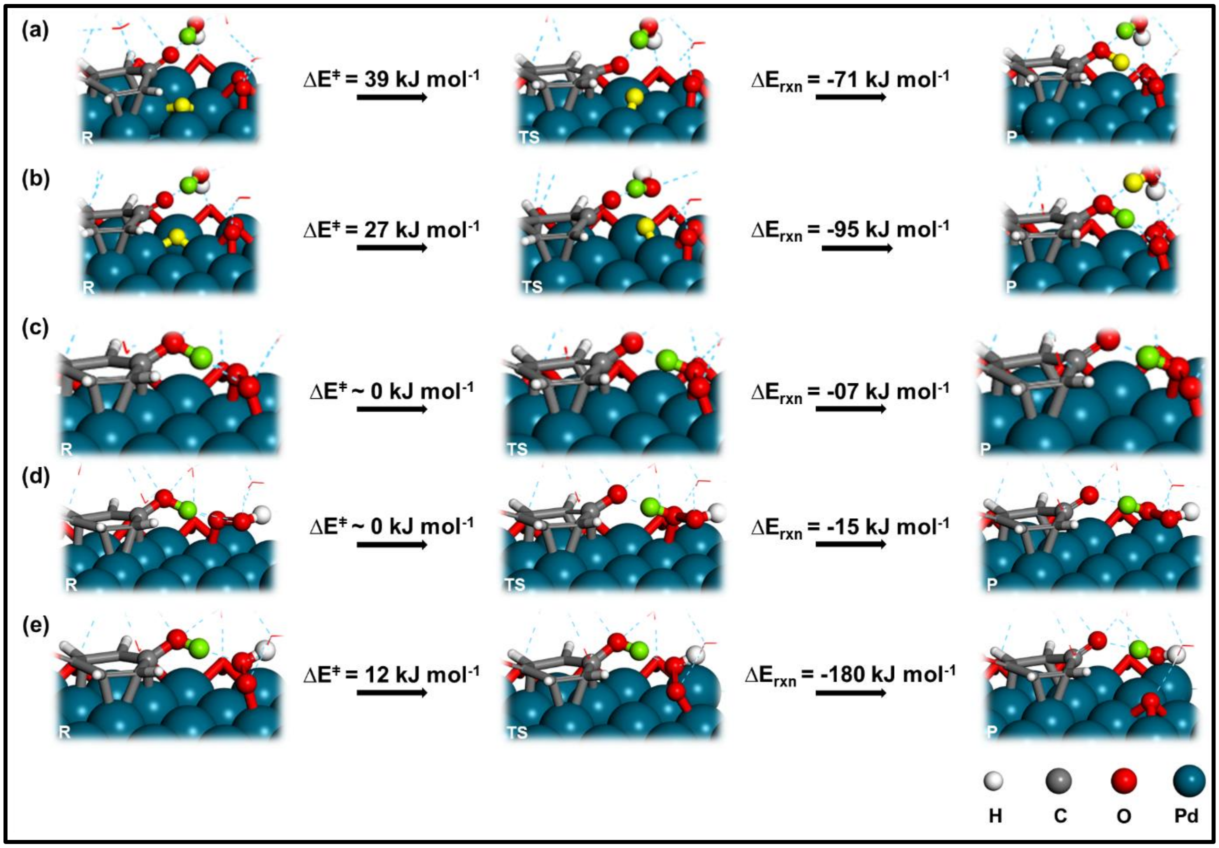

Figure S31. (a) Direct surface hydrogenation vs (b) proton coupled electron transfer step for hydrogenation of the BQ mediator in water. (c) Proton coupled electron transfer step for hydrogenation of O_2* from the hydrogenated BQ mediator. (d) Formation of H_2O_2 vs (e) formation of H_2O from the hydrogenated BQ mediator. Each step shows the corresponding reaction energies (ΔE_{rxn}) and intrinsic barriers ($\Delta E^{\ddagger}$) in kJ mol^{-1}. The simulated Pd (111) surfaces contain adsorbed O* (5/16 ML) and subsurface H_s* (5/16 ML) to represent the state of Pd NPs at reaction conditions determined by operando EXAFS in our prior work. The yellow hydrogen atoms are oxidized into a proton, and the green protons are transferred via a water molecule in the heterolytic mechanism.

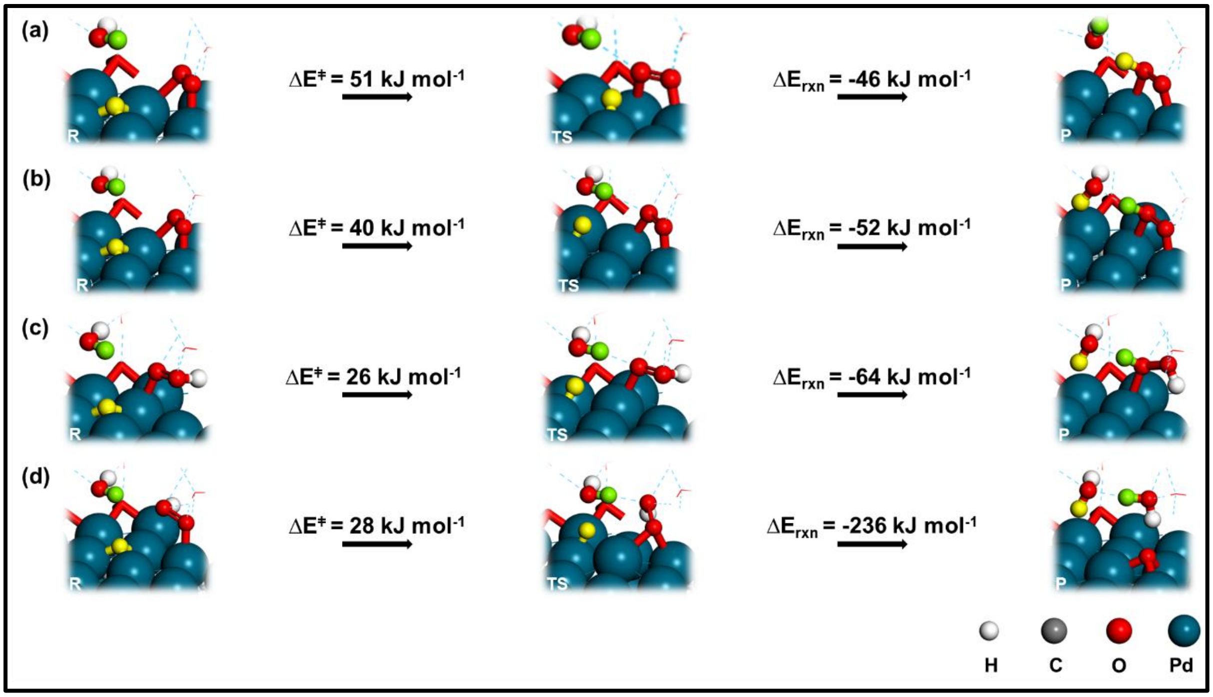

Figure S32. (a) Direct surface hydrogenation vs (b) proton coupled electron transfer step for hydrogenation of O_2* in water. (c) Formation of H_2O_2 vs (d) formation of H_2O via hydrogenation of OOH* in water. Each step shows the corresponding reaction energies (ΔE_{rxn}) and intrinsic barriers ($\Delta E^{\ddagger}$) in kJ mol^{-1}. The simulated Pd (111) surfaces contain adsorbed O* (5/16 ML) and subsurface H_s* (5/16 ML) to represent the state of Pd NPs at reaction conditions determined by operando EXAFS in our prior work. The yellow hydrogen atoms are oxidized into a proton, and the green protons are transferred via a water molecule in the heterolytic mechanism.

6. Kinetic Isotope Effects

Kinetic isotope effects (KIEs) were further examined computationally for the elementary steps associated with forming H_2O_2 and H_2O over the working Pd (111) surface comprised of 5/16 ML surface O* and 5/16 ML subsurface H_s* and the working surface covered with HKH to help rationalize the values obtained from experiments.

The calculations show a strong primary KIE for H vs. D (2.15) as compared to H_2O vs. D_2O (1.02) for the formation of H_2O_2 from adsorbed OOH* (Figure S33). This can be attributed to the more difficult part of this elementary step, which involves hydrogen oxidation on the surface to generate protons and electrons. Thus, replacing a surface H* with a surface D* results in a primary KIE of 2.15. The subsequent shuttling of the protons through the solution phase and coupled electron transfer through the metal is not difficult. Thus, replacing a solvent molecule from H_2O to D_2O shows no real effect on the isotopic substitution resulting in a KIE value of 1.02. This is consistent with experimental results which show a negligible KIE for H_2O/D_2O substitution in the formation of H_2O_2. Experiments, however, do not show a strong primary KIE at 1.18 for H vs. D substitution vs. the calculated value of 2.15. The small experimental KIE value can be attributed to the quasi-equilibrated steps associated with H_2 vs. D_2 adsorption that occur before the reaction, which lower the overall KIE of the combined reaction. Indeed, we calculated an equilibrium isotope effect of 0.55 in our prior studies,[1] which leads to an estimated KIE (k_{H2}/k_{D2} = 0.55x2.15 = 1.18) with near quantitative agreement with those measured experimentally.

Similar observations hold for the formation of H_2O from an OOH* intermediate over the working Pd (111) surface, with substitution of H* to D* showing a strong primary KIE of 2.09 compared to 1.01 for substitution of H_2O with D_2O (Figure S34).

The calculated results for the formation of H_2O_2 from adsorbed OOH* on the HKH-covered Pd (111) surface, where the proton is transferred from the partially hydrogenated HKH via the solution phase to the adsorbed OOH* intermediate, show a strong primary KIE of 5.74 for isotopic substitution of H_2O to D_2O (Figure S35). The transition state for this elementary step involves the transfer of the proton from the partially hydrogenated HKH to the OOH* surface intermediate, which results in a strong primary KIE. On the other hand, the formation of H_2O from the same OOH* intermediate over the HKH-covered surface, has a KIE value of only 1.43 (Figure S36). In this elementary step, the proton transfer is not involved in the transition state, but the transition state instead consists of the cleavage of O-O bond in the OOH* intermediate, resulting in a lower KIE value.

OOH* Reduction to H_2O_2 on Pd

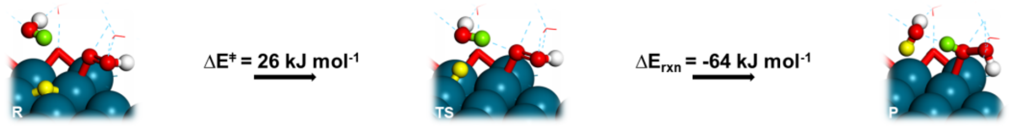

Isotope	Free Energy correction to R (kJ mol-1)	Free Energy correction to TS (kJ mol-1)	Free Energy correction to P (kJ mol-1)	ΔG‡ (kJ mol-1)	ΔΔG‡ (kJ mol-1)	KIE
H	16.66	10.50	30.12	-6.15	1.76	2.15
D	11.55	7.16	21.04	-4.39		
H_2O	29.79	30.77	29.91	0.98	0.05	1.02
D_2O	20.51	21.54	21.02	1.03		

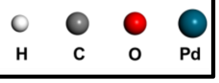

Figure S33. Calculated kinetic isotope effect for H vs. D and H_2O vs. D_2O for the formation of H_2O_2 from an OOH* intermediate on the working Pd (111) surface comprised of 5/16 ML of surface O* and 5/16 ML of subsurface H_s*.

OOH* Reduction to H_2O on Pd

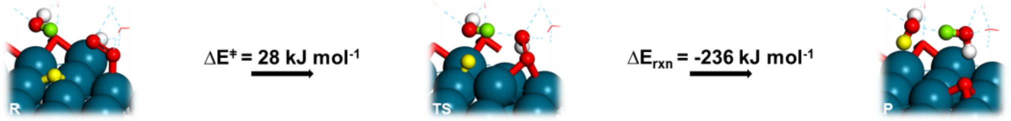

Isotope	Free Energy correction to R (kJ mol-1)	Free Energy correction to TS (kJ mol-1)	Free Energy correction to P (kJ mol-1)	ΔG‡ (kJ mol-1)	ΔΔG‡ (kJ mol-1)	KIE
H	16.67	10.48	29.63	-6.19	1.71	2.09
D	11.56	7.07	20.62	-4.49		
H_2O	29.90	30.93	30.94	1.03	0.03	1.01
D_2O	20.60	21.65	21.73	1.05		

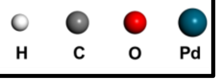

Figure S34. Calculated kinetic isotope effect for H vs. D and H_2O vs. D_2O for the formation of H_2O from an OOH* intermediate on the working Pd (111) surface comprised of 5/16 ML of surface O* and 5/16 ML of subsurface H_s*.

OOH* Reduction to H_2O_2 on HKH-Pd

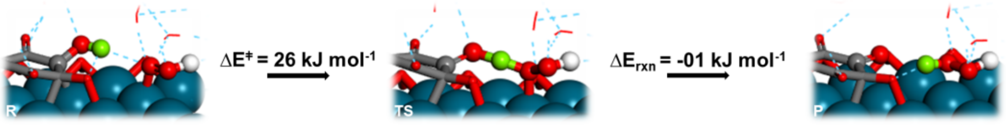

Isotope	Free Energy correction to R (kJ mol^{-1})	Free Energy correction to TS (kJ mol^{-1})	Free Energy correction to P (kJ mol^{-1})	ΔG‡ (kJ mol^{-1})	ΔΔG‡ (kJ mol^{-1})	KIE
H_2O	30.14	15.99	29.84	-14.15	4.33	5.74
D_2O	21.08	11.26	20.94	-9.82		

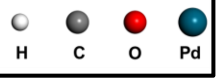

Figure S35. Calculated kinetic isotope effect for H_2O vs. D_2O for the formation of H_2O_2 from an OOH* intermediate on the HKH-covered working Pd (111) surface.

OOH* Reduction to H_2O on HKH-Pd

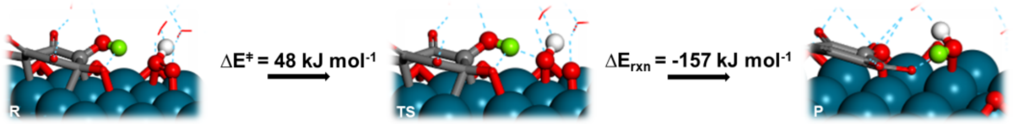

Isotope	Free Energy correction to R (kJ mol^{-1})	Free Energy correction to TS (kJ mol^{-1})	Free Energy correction to P (kJ mol^{-1})	ΔG‡ (kJ mol^{-1})	ΔΔG‡ (kJ mol^{-1})	KIE
H_2O	30.17	27.34	30.92	-2.83	0.89	1.43
D_2O	21.13	19.19	21.69	-1.94		

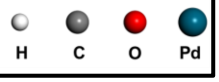

Figure S36. Calculated kinetic isotope effect for H_2O vs. D_2O for the formation of H_2O from an OOH* intermediate on the HKH-covered working Pd (111) surface.

7. Homogeneous Aqueous Phase Calculations of Quinone Redox Potentials

Different surface mediators have been employed in this work to improve the reactivity and selectivity towards two-proton two-electron reduction of O_2 to H_2O_2, as shown in Figure S37. We first discuss some key differences between these mediators. 1,4 Benzoquinone (BQ) and its derivatives (**[1-4]**) are conjugated but not aromatic. 1, 4 Naphthoquinone (NQ) (**[6]**) is conjugated, with the primary ring non-aromatic and the secondary ring aromatic. 1, 4 Anthraquinone (AQ) (**[7]**) is conjugated, with the primary ring non-aromatic and the secondary rings aromatic. Hexaketocyclohexane (HKH) (**[5]**) is a non-conjugated, non-aromatic mediator. BQ has a hydrogen bond donor count of zero and a hydrogen bond acceptor count 2. The hydrogen bonding nature of NQ and AQ is in line with BQ's hydrogen bond acceptor count of 2. HKH has a hydrogen bond donor count of zero and an acceptor count of 6. The solubility in the aqueous phase aligns with their hydrogen bonding and aromatic nature, with HKH being the most and AQ being the least soluble.

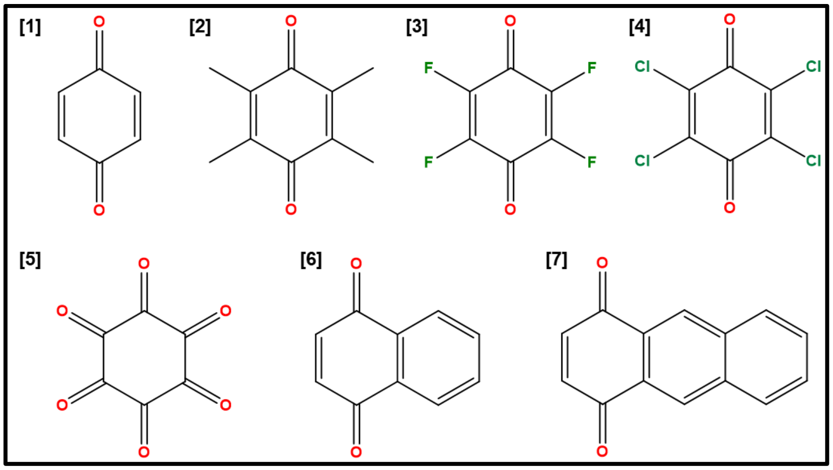

Figure S37. The different mediators investigated for homogenous phase calculations, including [1] 1,4-benzoquinone, [2] tetramethyl-1,4-benzoquinone, [3] tetrafluoro-1,4-benzoquinone, [4] tetrachloro-1,4-benzoquinone, [5] hexaketocyclohexane, [6] 1,4-napthoquinone, [7] 1,4-anthraquinone.

Homogeneous aqueous phase calculations with an implicit water solvent are employed to assess the different chemical nature of these mediators. As shown in Table S3, electron-donating substituents on 1,4 BQ reduce reduction potentials (less thermodynamically favorable), and electron-withdrawing substituents increase reduction potential (more thermodynamically favorable)). One- and two-electron reduction potentials show that HKH has the most positive and AQ the most negative potentials of all the mediators examined, indicating a thermodynamically favorable one- and two-electron reductions of HKH over other mediators. The lowest unoccupied molecular orbital (LUMO)-highest occupied molecular orbital (HOMO) gap for the neutral mediators is the lowest with HKH (481 kJ mol^{-1}) and highest with BQ (662 kJ mol^{-1}). Similarly, the LUMO-HOMO gap of the singly reduced mediators is again lowest with HKH (333 kJ mol^{-1}). These band gaps further indicate the more electrophilic nature of HKH over other mediators.

Hydrogenation of carbonyl bonds at the oxygen atom shows that the stability of the resulting carbon radical is related to the corresponding hydrogenation energies. The more stable the resulting carbon radical is, the less exergonic the reaction energy. With HKH, the resulting carbon radical is the least stable and has the most exergonic reaction energy (-73 kJ mol^{-1}). AQ has the least exergonic reaction energy as the corresponding carbon radical is the most stable (-6 kJ mol^{-1}). Similarly, hydrogenation of the carbonyl bonds at the carbon shows a similar trend, with HKH having the least endergonic reaction energy (119 kJ mol^{-1}) with the least stable oxo radical and AQ having the most endergonic reaction energy (179 kJ mol^{-1}). Hydrogenation energies show that hydrogenation at the oxygen atom will be more favorable than at the carbon atom, as the former forms a carbon radical that can delocalize in the mediator.

Table S3. LUMO-HOMO gap of the neutral and singly reduced, one and two-electron reduction potentials, and O-H and C-H hydrogenation energies of the mediators

Mediator	LUMO-HOMO gap ^{a} (kJ mol^{-1})	One e$^-$ Reduction Potential (V vs. SHE)	LUMO-HOMO gap ^{b} (kJ mol^{-1})	Two e$^-$ Reduction Potential (V vs. SHE)	Hydrogenation Energy for O-H formation (kJ mol^{-1})	Hydrogenation Energy for C-H formation (kJ mol^{-1})
1	662	0.08	403	-0.33	-41	165
2	600	-0.3	389	-0.64	-14	171
3	618	0.59	407	0.14	-39	176
4	560	0.57	385	0.14	-37	155
5	481	1.31	333	0.91	-73	119
6	620	-0.18	375	-0.54	-16	174
7	517	-0.29	363	-0.62	-6	179

^{a} neutral mediator, ^{b} singly reduced mediator

Lowest unoccupied molecular orbitals (LUMO) on the mediators show that the LUMO is majorly located in the rings for the quinone mediators and the carbonyl oxygens, as shown in Figure S38. HKH on the other hand, having six carbonyl oxygens, affords LUMO delocalization evenly over all six carbonyl oxygens. These oxygens are equivalent, thus can allow HKH to cover a higher chemisorbed surface area on the Pd surface compared to quinone mediators with one ring. Further, the ring in HKH is homogeneous in nature shown by the homogeneous distribution of the LUMO lobes. This contrasts with the quinone mediators, which show discontinuous LUMO lobes in the rings.

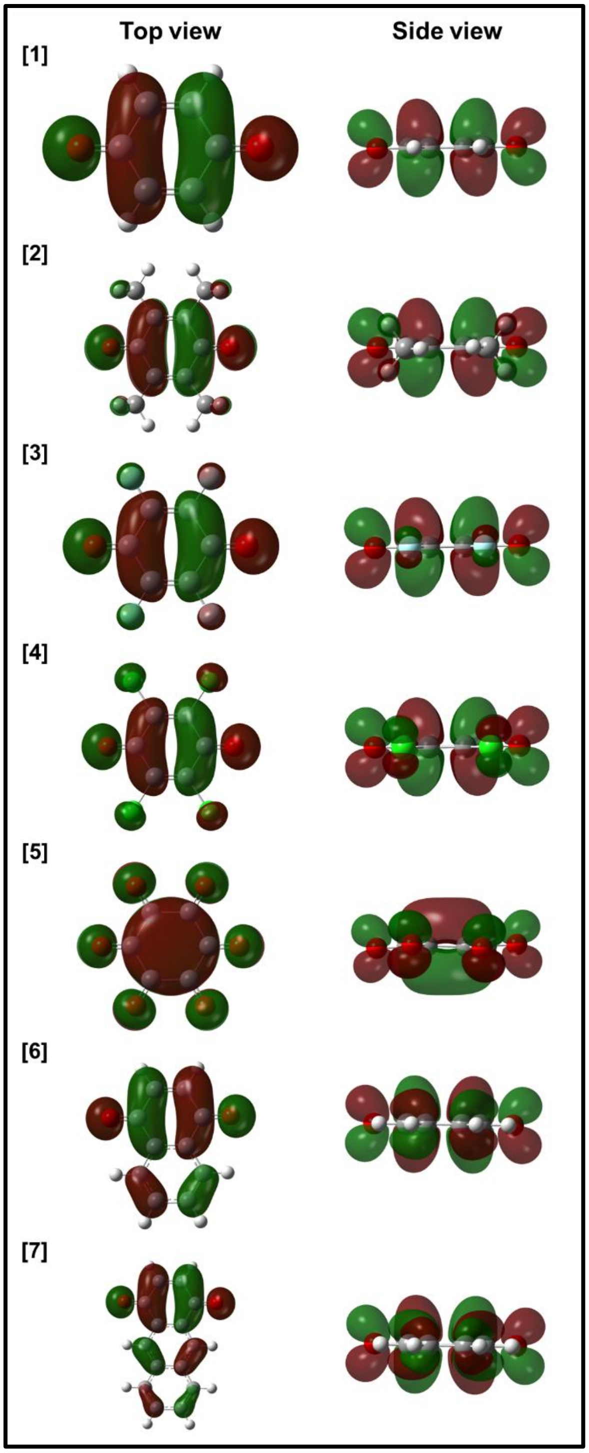

Figure S38. LUMO for the different mediators showing the top and side view of [1] 1,4-benzoquinone, [2] tetramethyl-1,4-benzoquinone, [3] tetrafluoro-1,4-benzoquinone, [4] tetrachloro-1,4-benzoquinone, [5] hexaketocyclohexane, [6] 1,4-napthoquinone, [7] 1,4-anthraquinone. Oxygen in red, carbon in gray, hydrogen in white. The two orbital colors green and maroon show the opposite phases of wave function in each region.

8. Density of States Calculations

Density of states (DOS) and partial DOS calculations were performed for BQ and HKH mediators for these different surface coverages of O* and H_s* atoms. The physisorbed state corresponds to mediator at nearly 10 Å away from the Pd surface, which then undergoes chemisorption on the Pd surface as shown in Figure S25, 28. During the adsorption, none of the surface and sub-surface bound O*, O_2* and H_s* species undergo displacement. DOS calculations are carried out employing implicit solvation with water as a solvent.

As shown in Figure S39, the p orbitals of the carbon and oxygen of the mediator show mixing with the d orbitals of the Pd surface. The degree of mixing is greater with HKH as compared to BQ. This trend is similar when increasing H_s* coverage (Figure S40) or decreasing the H_s* coverage (Figure S41). However, this mixing is reduced when increasing O* coverage (Figure S42) and is enhanced when decreasing O* coverage (Figure S43). These observations are in line with the adsorption energies in Figure S26. Thus, HKH has a stronger chemisorption with the catalyst surface, which is enhanced at lower O* coverage and diminished at higher O* coverage.

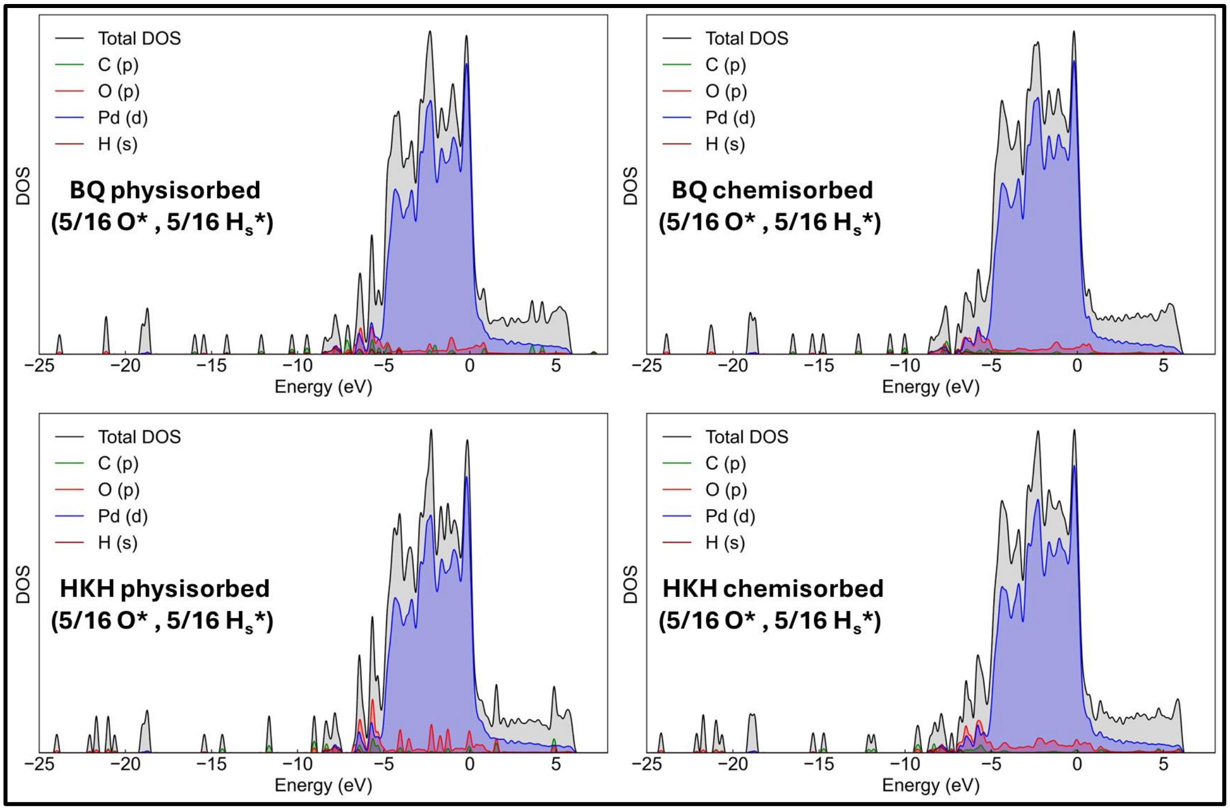

Figure S39. DOS and PDOS for BQ and HKH before and after chemisorption for (5/16) ML O* and (5/16) ML H_s* coverage in an implicit solvent. Total DOS in black, PDOS for d states of Pd in blue, p states of O in red, p states of C in green, and s state of H in maroon.

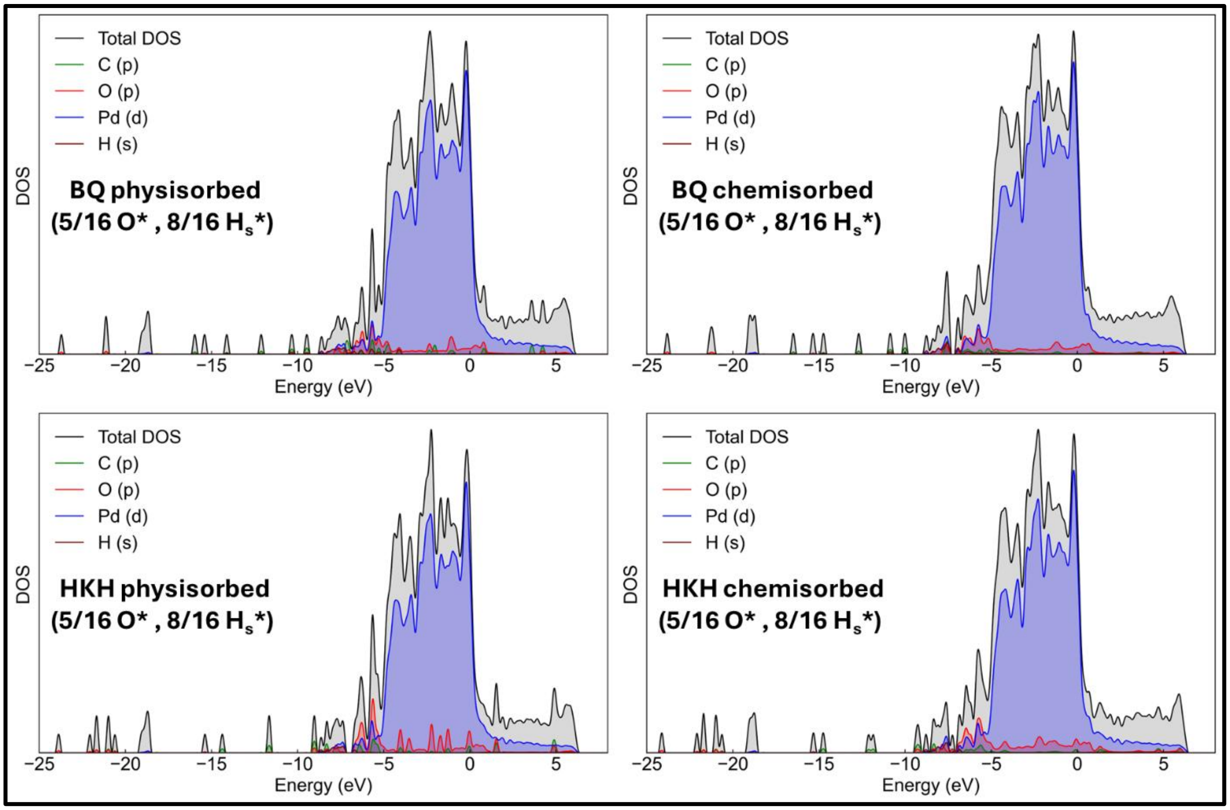

Figure S40. DOS and PDOS for BQ and HKH before and after chemisorption for (5/16) ML O* and (8/16) ML H_s* coverage in an implicit solvent. Total DOS in black, PDOS for d states of Pd in blue, p states of O in red, p states of C in green, and s state of H in maroon.

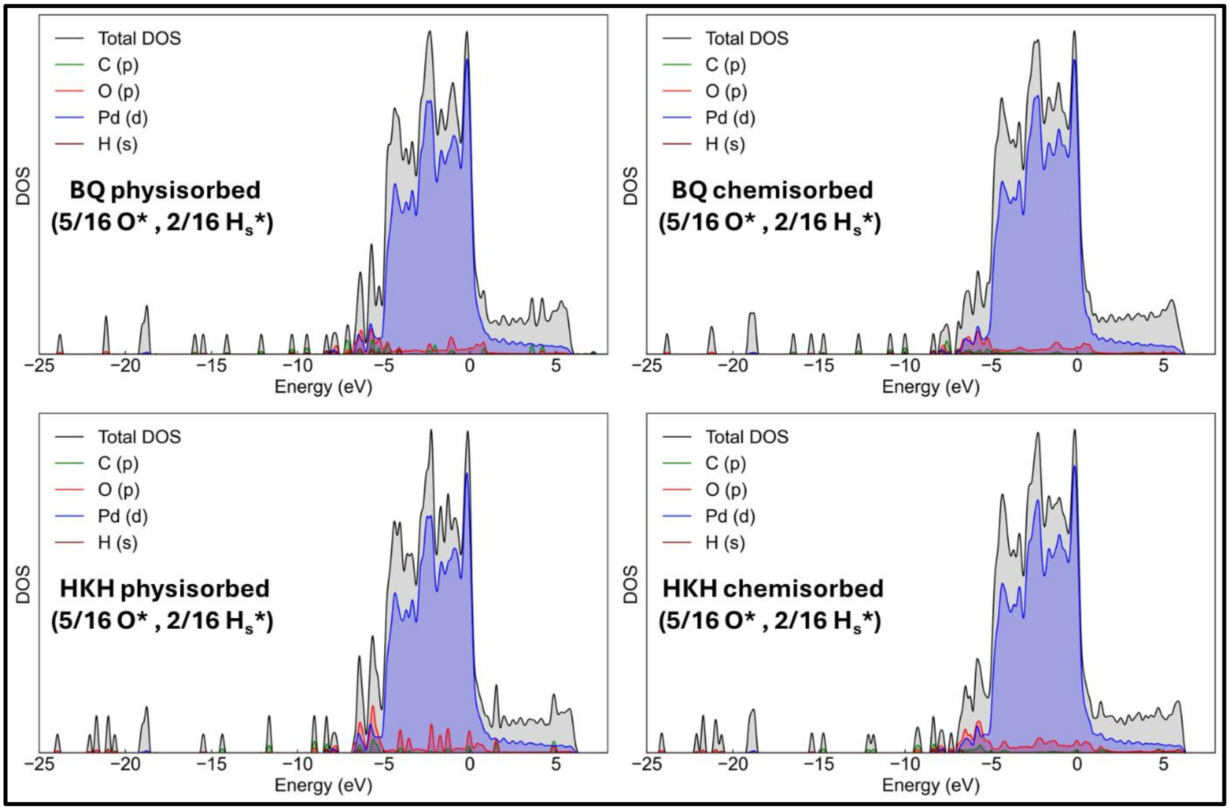

Figure S41. DOS and PDOS for BQ and HKH before and after chemisorption for (5/16) ML O* and (2/16) ML H_s* coverage in an implicit solvent. Total DOS in black, PDOS for d states of Pd in blue, p states of O in red, p states of C in green, and s state of H in maroon.

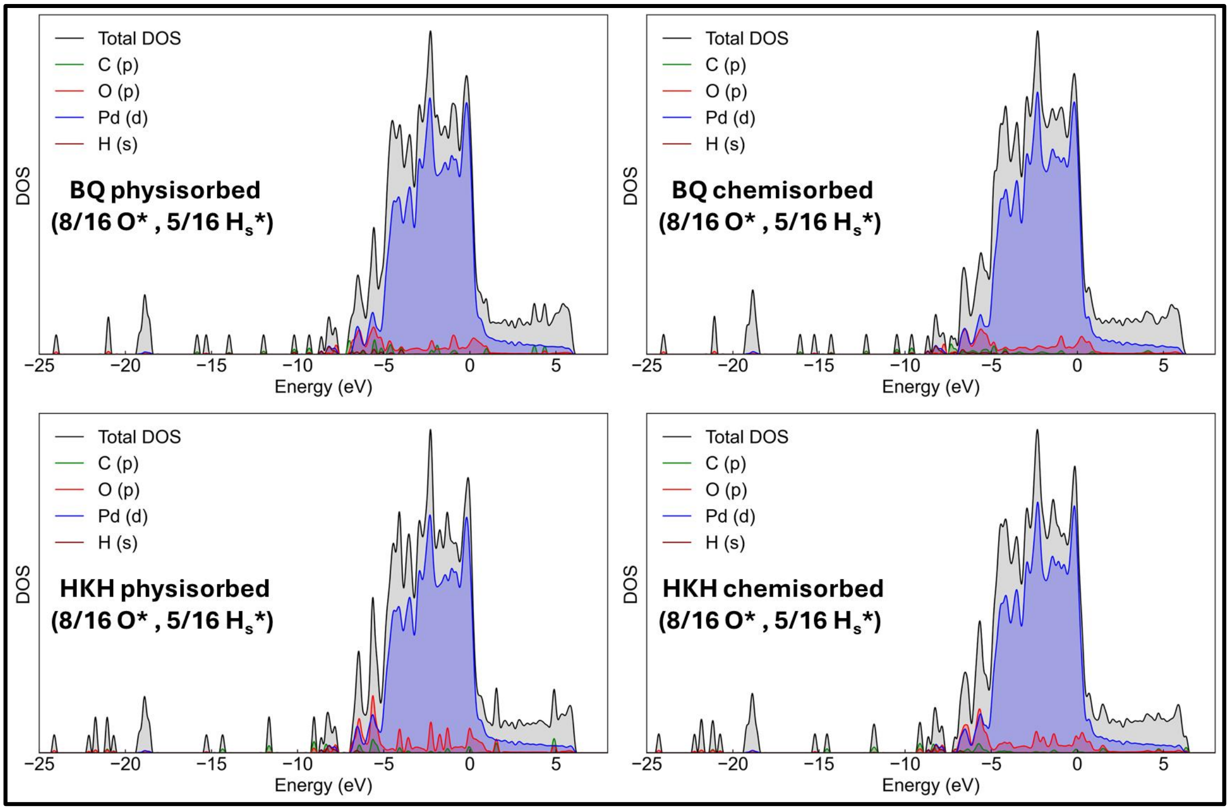

Figure S42. DOS and PDOS for BQ and HKH before and after chemisorption for (8/16) ML O* and (5/16) ML H_s* coverage in an implicit solvent. Total DOS in black, PDOS for d states of Pd in blue, p states of O in red, p states of C in green, and s state of H in maroon.

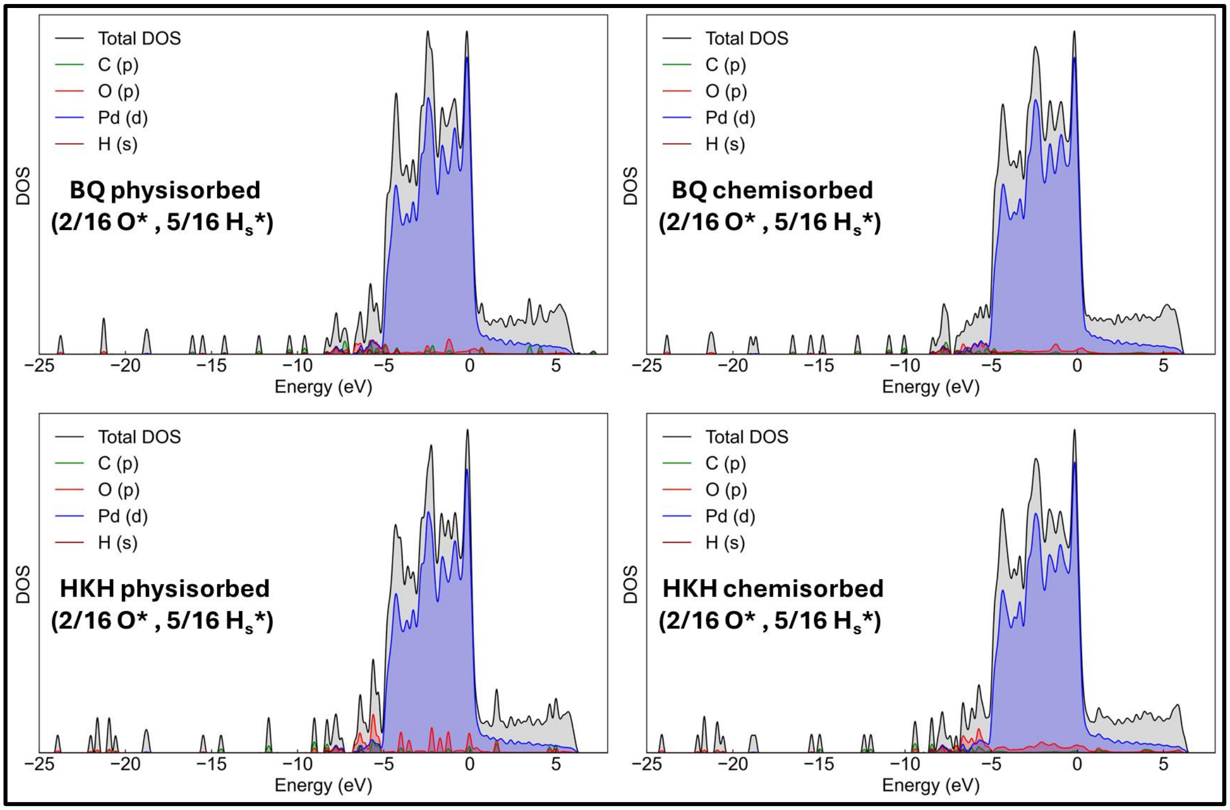

Figure S43. DOS and PDOS for BQ and HKH before and after chemisorption for (2/16) ML O* and (5/16) ML H_s* coverage in an implicit solvent. Total DOS in black, PDOS for d states of Pd in blue, p states of O in red, p states of C in green, and s state of H in maroon.

Supporting Experimental Methods

SM.1 Catalyst Preparation

The Pd-SiO_2 catalyst (0.04 wt% Pd) was synthesized by strong electrostatic adsorption of cationic Pd onto mesoporous SiO_2 (20 g; Davisil 646) using a Pd(NO_3)$_2$ precursor (Sigma-Aldrich 76070). First, a solution of 3.8 mM of the Pd salt (500 mL) was prepared. Then, in a separate beaker, the SiO_2 was soaked in a solution of DI H_2O and NH_4OH (0.5 M, 500 mL; Macron 6665) to deprotonate the hydroxylated surface of the support. The suspension was stirred continuously for 10 min, after which the solution of Pd(NO_3)$_2$ was added. The mixture was then stirred intermittently every 10 min for 1 h and left overnight to allow cationic Pd species to adsorb to the anionic surface of the support. The resulting Pd-SiO_2 precursor was washed with 1 L of DI H_2O and then vacuum filtered for 24 h. The dried material was then placed within a quartz tube furnace and heated at a rate of 5 K min^{-1} to a temperature of 673 K and held for 4 h under a flowing mixture (300 cm^3 min^{-1}) of He (67 kPa; Airgas, UHP 99.999%) and dry air (7 kPa O_2, 26 kPa N_2; Airgas, UHP 99.999%). The sample was allowed to cool to a temperature of 573 K and held for 4 h under a flowing mixture (200 cm^3 min^{-1}) of He (81 kPa; Airgas, UHP 99.999%) and H_2 (20 kPa H_2, Airgas, UHP 99.999%). Upon cooling to room temperature, the resulting Pd sample was passivated in a highly dilute mixture of air (0.1 kPa O_2, 0.4 kPa N_2; 1 cm^3 min^{-1}) and He (100 kPa; 199 cm^3 min^{-1}) for 1 h before removal from the furnace.

The Pd-SiO_2 catalyst was treated with organic molecules to prepare organic-treated catalytic materials. The organic species used in this study include: hexaketocyclohexane octahydrate (Sigma-Aldrich, >97%), 1,4-benzoquinone (Acros Organics, 99%), 1,4-naphthoquinone (Sigma-Aldrich, 97%), 1,4-anthraquinone (Sigma-Aldrich, 97%), duroquinone (Sigma-Aldrich, 97%), tetrafluoro-1,4-benzoquinone (Sigma-Aldrich, 97%), tetrachloro-1,4-benzoquinone (Sigma-Aldrich, 99%), benzene (Sigma-Aldrich, 99%), 1,2,4,5-tetrafluorobenzene (Sigma-Aldrich, >99%), 1,2,4,5-tetrachlorobenzene (Sigma-Aldrich, 98%), formalin (Electron Microscopy Science, 37 wt% CH_2O, 11 wt% CH_3OH, 52 wt% H_2O), acetaldehyde (Sigma-Aldrich, 40 wt% in H_2O), propionaldehyde (Sigma-Aldrich, 97%), acetone (Fisher 99.8%), 2-butanone (Sigma-Aldrich, >99%), glyoxal (Sigma-Aldrich, 40 wt% in H_2O), tetrahydrofuran (Sigma-Aldrich, >99.9%), and n-hexane (Supelco, 99%).

Analogs of 1,4-benzoquinone ($C_6X_4O_2$) and benzene ($C_6X_4H_2$) were dissolved in dioxane (20 mL, Sigma-Aldrich, 99.8%), a solvent that negligibly affects reaction rates (*vide infra*; Figure S17). Solutions were prepared with concentrations of 230 mM for these analogs, which was sufficient to saturate the surface of Pd nanoparticles with the organic (*vide infra;* Figure *5*). A series of solutions containing 1,4-naphthoquinone (100-1000 mM) or 1,4-anthraquinone (5-25 mM) were prepared in dioxane with varying concentrations to investigate the effects of the concentration of the organic species on rates and selectivities. Solutions containing hexaketocyclohexane (230 mM) were prepared with DI H_2O as the solvent because this molecule remains insoluble in dioxane. Finally, non-polyconjugated organics, including C_1-C_3 carbonyl compounds (formalin, acetaldehyde, propionaldehyde, acetone, 2-butanone, and glyoxal) were adsorbed to Pd-SiO_2 catalysts from the neat solution of the organic molecules due to their weak binding at surfaces. In parallel, Pd-SiO_2 was soaked in neat aliphatic solvents (tetrahydrofuran, n-

hexane, and dioxane) to create materials for control experiments. Each solution was sparged with a flowing mixture (100 cm^3 min^{-1}) of He (81 kPa) and H_2 (20 kPa H_2) for at least 10 min with the intent of removing air from the solvent and reducing the Pd nanoparticles before the adsorption of organics. Then, 300-1000 mg of the Pd-SiO_2 sample was added to the solution and allowed to soak for 1 h under a continuous flow of the H_2 and He mixture. Afterward, the gas flow was stopped, the vessel was sealed, and the sample was soaked for an additional 3 h. The resulting solids were vacuum-filtered overnight at ambient conditions to yield the organic-treated samples for catalytic measurements. Notably, catalysts were soaked in supersaturated solutions that left large factions of the solid organic mixed with the catalyst. However, any excess organic not removed by vacuum filtration was removed from the sample upon contact with a continuous flow of water (35 cm^3 min^{-1}) during catalysis. Samples treated with hexaketocyclohexane produced a physical mixture of the organic and Pd-SiO_2, containing nearly equal masses. Loading this mixture of hexaketocyclohexane and Pd-SiO_2 was critical to ensure the Pd surface was fully saturated with the organic during catalysis.

SM.2 Characterization of Catalytic Materials

SM2.1 Determination of Pd Nanoparticle Size and Composition

The numerical average diameter ($<d_{TEM,N}>$) of Pd nanoparticles was calculated from the mean diameter of particle size distributions obtained by bright-field transmission electron microscopy (TEM; Hitachi, H-9500) of more than 100 nanoparticles on each material. Each sample was prepared by grinding the catalyst into a fine powder (<200 mesh), which was dispersed in ethanol (Decon Laboratories, >99.9%) and dripped onto a Cu holey-carbon TEM grid (200 mesh, Ted Pella Inc.). The surface area normalized average diameter ($<d_{TEM,S}>$) for each catalyst was calculated using equation 1.

$$< d_{TEM,S} > = \frac{\sum_i n_i \, d_i^3}{\sum_i n_i \, d_i^2} \tag{1}$$

where n_i is the number of nanoparticles with the diameter d_i. Figure 1 shows a representative TEM image of 4 nm Pd nanoparticles on SiO_2 with an inset histogram of the particle size distribution.

SM2.2 Examination of Pd Surface Residues by Infrared Spectroscopy

Ex situ transmission infrared spectra were obtained from a Fourier transform infrared spectrometer (Bruker, Tensor 37) and used to characterize adsorbates present upon untreated and organic-treated samples before and after catalytic measurements. Specifically, complementary infrared spectra of CO adsorbed on these samples elucidate how available active sites change in the presence of organic molecules. Here, samples were prepared by grinding catalysts (~60 mg) into a fine powder and pelletizing the powder into self-supporting ~20 mm diameter discs using a laboratory hydraulic press (Carver, model C). These discs were set between two stainless steel

retaining rings and loaded into a custom-built transmission infrared cell with CaF_2 windows, as described previously.[1-3] The cell was sealed by compressing natural graphite ferrules (Chromalytic Technology Pty. Ltd.) with another stainless-steel retaining ring on the cell exterior. Gaseous H_2, CO, He, and O_2 were introduced to the cell by digital mass flow controllers (Alicat, MC Series) mounted to a gas-handling manifold connected to the cell.

Background spectra of organic-treated samples were collected in flowing (30 cm^3 min^{-1}) He gas (101 kPa) at 303 K. Then, the gas composition was changed to 20 kPa H_2 and 81 kPa He, and the sample was heated to 373 K at 5 K min^{-1} and held at 373 K for 1 h before cooling to 303 K in pure He. Subsequent background spectra were collected to determine the loss of H_2O from this initial thermal treatment. Then, the gas composition was changed to 0.2 kPa CO and 101 kPa He, and spectra were collected until the sample reached a steady state. Afterward, the gas composition was changed to 20 kPa O_2 and 81 kPa He, and the sample was heated to 573 K at 5 K min^{-1} and held at 573 K for 1 h before switching the gas to 20 kPa H_2 and 81 kPa He and held at 573 K 1 h longer. Again, the sample was cooled to 303 K in pure He, and another background was taken to determine the loss of organic features from oxidation. Last, the gas composition was changed to 0.2 kPa CO and 101 kPa He, and spectra were collected until the sample reached a steady-state, informing the structure of the catalyst in the absence of organic- or H_2O-derived adsorbates. The resulting spectra were then compared using a consistent normalization of the Si–O–Si overtones of the SiO_2 support, enabling meaningful comparisons of features normalized by an internal standard (*vide infra*).

In situ Fourier-transform infrared spectroscopy attenuated total reflectance (FTIR-ATR; Bruker, Vertex 70) was used to determine the structure of adsorbed organics on the surface of ligand-treated samples under reaction conditions reflective of steady-state rate measurements (200 kPa H_2, 60 kPa O_2). Here, a 10 wt% Pd-SiO_2 was crushed into a fine powder (200 mesh) and dispersed in acetone before dip-coating the catalyst onto a ZnSe cylindrical internal reflection element (IRE, International Crystal Laboratories). The coated IRE was then loaded into an FTIR-ATR flow cell (Axiom, TNL-120) that was mounted into the FTIR spectrometer. The cell was then pretreated in a flowing (30 cm^3 min^{-1}) mixture of O_2 (20 kPa) and He gas (81 kPa) at 373 K for 1 h using a resistive heating cartridge within the cell, controlled by an electronic temperature controller (Watlow, EZ-Zone). Then, the gas composition was changed to a mixture of H_2 (20 kPa) and He gas (81 kPa) at 373 K for 1h before cooling the sample to room temperature in pure He (101 kPa). Then, two solutions of DI H_2O were sparged with H_2 (101 kPa) and O_2 (101 kPa), respectively, before pumping them through the flow cell (10 mL min^{-1}, 75 kPa H_2, 25 kPa O_2, 298 K) using two HPLC pumps (Teledyne SSI, LS-class). Then, spectra were collected until the sample reached steady-state before taking a new background spectrum. Afterward, the pump inlet of the H_2-sparged DI H_2O was switched to a 5 mM solution of hexaketocyclohexane, separately sparged with H_2 (101 kPa). Finally, spectra were collected until reaching a new steady state in the presence of the organic solution. Note that the ambient pressure cell could not achieve the same pressure of H_2 and O_2 used in reactor measurements (200 kPa H_2, 60 kPa O_2), so a similar ratio of H_2 to O_2 (3:1) was used for in situ FTIR-ATR measurements.

SM2.3 Quantification of Organic Species by Temperature Programmed Oxidation and Desorption

Temperature-programmed oxidation (TPO) and temperature-programmed desorption (TPD) profiles were measured on untreated and organic-treated Pd materials before and after kinetic measurements to quantify the moles of organic species adsorbed to the catalyst surface. Catalytic samples (250 mg) were loaded into a fused quartz tube (12 mm outer diameter, 10 mm inner diameter, 38 cm long) containing a quartz frit to support the catalyst. A thermocouple (Omega, type K) was located within an indentation in the outer wall of the quartz tube near the catalyst bed. The thermocouple was connected to a temperature controller (Watlow, EZ-ZONE PM) to control the temperature of a split tube furnace (Applied Test Systems, Series 3210) surrounding the quartz tube and catalyst. Digital mass flow controllers (MFC; Porter, series 601) were used to flow a certified mixture of O_2 (5% O_2/N_2, Airgas, 99.999%) or He (UHP, Airgas, 99.999%) at known flow rates through a gas manifold (equipped with a reactor bypass) to the catalyst. The composition of the effluent gas was determined using a quadrupole mass spectrometer (QMS; Pfeiffer Vacuum, Thermostar) with a heated quartz capillary located beneath the catalyst bed. The inlet of the QMS was maintained at elevated temperatures (433 K) to prevent the condensation of H_2O vapor that evolved during the oxidation of organics. Each TPO profile was obtained by purging the reactor with a mixture of O_2 and N_2 (5 kPa O_2, 96 kPa N_2, 100 cm^3 min^{-1}) at ambient temperature (~303 K) to remove atmospheric CO_2 from the vessel. TPD measurements were performed equivalently to TPO measurements but used He (101 kPa He, 100 cm^3 min^{-1}) as the carrier gas. In both forms of experiments, the signal of the QMS was allowed to stabilize before heating the furnace from ambient temperature to 973 K at 5 K min^{-1} while monitoring evolving CO_2 (44 m/z) and carrier gases (e.g., N_2, He). Integration of such profiles yields the moles of CO_2 that evolve from the samples, which was calibrated using TPO and TPD measurements of the decomposition of known quantities of $NaHCO_3$ (Sigma-Aldrich, >99.5%; Figure S2).

SM2.4 Determination of Elemental Composition by EDXRF

The metal loadings were determined by energy-dispersive X-ray fluorescence (EDXRF; Shimadzu EDX-7000) spectroscopy of Pd-SiO_2 samples (Table S1). Samples were prepared by loading ~50 mg of catalytic materials into sample cups (5 mm ID), sealed on the top and bottom with a thin mylar film. Quantitative spectra were collected on the sample using a Rh X-ray source and Mn K-alpha solid-state detector. The sample chamber was purged with a He environment and given at least 2 minutes to purge before conducting the measurement. Data was collected and processed using the PCEDX-Navi and PCEDX-Pro software with appropriate elemental calibrations. The molar compositions of Si and Pd were determined and converted into elemental weight loadings of Pd by assuming all elemental contributions come strictly from Si and Pd.

SM.3 Steady-State Reaction Rate Measurements within a Fixed-bed Reactor

All steady-state rates of H_2O_2 ($r_{H_2O_2}$) and H_2O (r_{H_2O}) formation were measured in a continuous-flow fixed-bed reactor (48 cm length, 1 cm inner diameter) housed within a stainless-steel cooling jacket (Figure S3).[1, 4, 5] The reactor was loaded with catalyst (0.25-0.7 g) diluted with SiO_2 (1.8-2.2 g, Davisil 646), supported by plugs of glass wool (~10 mg) and borosilicate glass rods (8 mm diameter). These rods were secured between silver-coated fritted VCR gaskets (Swagelok, SS-4-VCR-2-60M), which were also used to seal the reactor. The temperature was controlled across the

reactor by flowing aqueous ethylene glycol (50% volume; Fisher Scientific E178, 99.8%) through the cooling jacket from a recirculating temperature bath (Cole-Parmer Polystat). The temperature within the reactor was monitored using a K-type thermocouple attached to a cooling jacket, contacting the wall surrounding the catalyst bed. H_2 and O_2 compositions in the reactor were controlled by flowing certified gas mixtures (25% H_2/N_2, 99.9% D_2, 5% O_2/N_2, Airgas, 99.999%) through digital mass-flow controllers (Bronkhorst, F-211CV). *Warning: pressurized mixtures of H_2 and O_2 are explosive when the two components exceed a mole fraction of 0.05.* Before contacting the catalyst, the gaseous reactant stream mixed with the solvent (DI water [> 17.8 MΩ cm] or 70 %(v/v) CH_3OH/H_2O [>99.8%, Macron 3016]) and was delivered by an HPLC pump (SSI, LS class). The pressure of the resultant gas-liquid mixture was maintained by a back-pressure regulator (Equilibar, LF) and controlled by an electronic pressure regulator (Equilibar, GP1). The upstream pressure of the reactor was monitored by a digital pressure transducer (Omega, PXM409-USBH).

Sampling and analysis of the liquid and gaseous effluent streams were automated and operated continuously. The reactor effluent entered a gas-liquid separator (GLS), and the gas stream flowed to a gas chromatograph (Agilent, 7890B) equipped with a capillary column (Vici, Molecular Sieve 5Å, 30 m x 0.53 mm x 20 μm) and a thermal conductivity detector using Ar gas (Airgas, 99.999%) as the reference gas. Gas chromatograms of catalytic measurements were compared to reference chromatograms using a bypass reactor without catalytic materials to calculate the rates of H_2 ($-r_{H_2}$) and O_2 ($-r_{O_2}$) consumption by a given catalyst.

The liquid fraction of the GLS was drained at 10-minute intervals by an electronic valve (ALSCO Inc., LEV025PL) and flowed into an electronic two-position valve (Vici Valco, 10 port EPC10W), which injected 1 cm^3 of the liquid effluent and 1 cm^3 of a colorimetric titrant (12 mM neocuproine [Sigma-Aldrich, >99%], 8.3 mM $CuSO_4$ [Fisher Scientific, >98.6%], 25 vol% (v/v) ethanol/deionized water mixture [Decon Laboratories, >99.9%]) into test tubes held in an automated fraction collector (Biorad, 2110). Each tube was analyzed by a UV-vis spectrophotometer (Spectronic, 20 Genesys) at a wavelength of 454 nm to measure the H_2O_2 concentration using a corresponding calibration curve to determine rates of H_2O_2 formation ($r_{H_2O_2}$). Reported formation rates were determined by normalizing the measured rates by the total metal content of the materials, and turnover rates are estimated from the number of metal atoms on the surface of nanoparticles, to determine the fraction estimated by TEM measurements (Figure S1). Reported H_2O_2 selectivities were calculated by dividing the rate of H_2O_2 formation by the rate of H_2 consumption of H_2 gas that forms the H_2O_2 product ($r_{H_2O_2}/-r_{H_2}$).

Note that reactor measurements were also conducted using untreated SiO_2 without catalytic nanoparticles, which showed no measurable formation of H_2O_2 (Figure S4). Measurements were also performed on similar SiO_2 samples prepared with 1,4-benzoquinone and hexaketocylclohexane equivalent to Pd-SiO_2 samples (Section 2.1), showing some initial reactivity with the neocuproine solution. However, these samples behave like SiO_2 after ~1 h on stream, meaning measurements of H_2O_2 formation are meaningful after this period. All experiments were conducted at a liquid flow rate of 35 cm^3 min^{-1} to avoid external mass transfer limitations.[1] Moreover, all samples satisfy the Madon-Boudart Criterion, enabling meaningful comparisons of

catalytic rates and stability without mass transfer constraints.[5-7] For activation enthalpy measurements, reported rates were corrected by accounting for the rate of deactivation, which assumes that these changes arise from an exponential reduction in the number of active sites over time.[8] These corrections were determined by returning to the initial condition and quantifying the deactivation over time.

SM.4 Transient Rate Measurements Using Semibatch Reactor

Transient concentration profiles were measured in round-bottom three-neck flasks (Wilmad Lab Glass, 50 – 100 cm^3) in a semi-batch configuration (Figure S5).[1] The top neck of each flask was connected to a condenser, which was chilled to 273 K with a 20/80 (v/v) mixture of ethylene glycol (>99.8%, Fisher Scientific E178) and DI water that flowed from a refrigerated recirculating bath (Neslab ENDOCAL). The reactant gas mixture (99.999% H_2 and 5% O_2/N_2, Airgas) was fed into the three-neck flasks using digital mass-flow controllers (Alicat MC series) connected to custom-built gas dispersion tubes (GDT). Each GDT was placed into 80 cm^3 of a solvent such that the GDT frits (40-60 μm) were completely submerged. Specifically, this study used DI water and heavy water (D_2O; Cambridge Isotopes, 99.9 atom % D) as solvents. All experiments were conducted at 298 K at ambient pressure, and the solution media was stirred at 1500 rpm with magnetic stir bars to avoid external mass transfer limitations.[1] Each solvent solution was saturated with gas for 10 min before the catalyst was injected into the flask (50 mg). Aliquots (~1 cm^3) were extracted every 5 min during the first 30 min, every 10 min for the following 30 min, and then every hour for 3 h for this measurement. These samples were titrated with a neocuproine solution to determine concentrations of H_2O_2, as described in Section 2.3. The resulting concentration profiles were then fitted to determine apparent rate constants for the formation of H_2O_2 using methods reported elsewhere.[1] Overall, semi-batch measurements were used to compare rates within small volumes of isotopic solvent since fixed-bed measurements require costly flow rates.

SM.5 Computational Methods

Homogeneous aqueous phase density functional theory (DFT) calculations were carried out to assess the electronic properties and chemical nature of the various mediators using the Gaussian 16 software package[9] with the M06-2X exchange-correlation functional,[10] 6-311++G(d,p) basis set with auto density fitting,[11] and an implicit SMD solvation model for water as a solvent.[12] Self-consistent field (SCF) cycles were converged to 10^{-8} Ha. Geometry optimizations were converged after maximum force, root-mean-square force, maximum displacement, and root-mean-square displacement were below $4.5*10^{-4}$ Ha/a_0, $3.0*10^{-4}$ Ha/a_0, $1.8*10^{-3}$ a_0, and $1.2*10^{-3}$ a_0, respectively, a_0 being the Bohr radius. These calculations were carried out using an ultrafine integration grid with no symmetry and SCF parameters of tight, intrep, and xqc. All stationary points were confirmed to have no negative vibrational frequencies for the ground state calculations.

Periodic plane-wave DFT calculations were carried out using the Vienna Ab initio Simulation Package (VASP) [13, 14] to model the mediated reactions of H_2 and O_2 on palladium. A slab comprised of a 4 x 4 unit cell with four layers of the Pd(111) surface was employed in all surface calculations as a simple first-order model of the experimental silica-supported Pd

nanoparticles. The bottom two layers of the slab were held fixed at the bulk positions of the Pd lattice while the top two layers were allowed to relax. Previous experimental and computational results suggest that the steady-state surface coverages of atomically adsorbed oxygen (O*) under working reaction conditions are ~5/16 ML. In contrast, the bulk Pd comprises ~5/16 ML of subsurface hydrogen atoms (H_s*). These same coverages are used herein as the reaction conditions are the same. These coverages are consistent with *operando* extended x-ray absorption fine structure (EXAFS) measurements and established adlayer structures.[1, 15] While the O* atoms occupy three-fold face-centered cubic (FCC) sites, subsurface H_s* atoms occupy the octahedral sites. As shown in our previous work, the dissociative adsorption of H_2 has a small barrier and is highly reversible; hence, one surface hydrogen with other surface adsorbates (O*, mediators, O_2*) has been chosen as a resting state for the calculations.[1] Initial calculations used an implicit solvation model to provide reliable initial estimates of the reactant, intermediate, transition, and product state structures and establish approximate energetics. A vacuum region of nearly 23 Å was used for all implicit solvent calculations. Density of states (DOS) and partial DOS calculations were carried out to elucidate the nature of the bonding for different surface coverages of O* and subsurface coverages of H_s*.

Ab initio molecular dynamics (AIMD) simulations were subsequently carried out to incorporate explicit solvent molecules in the model and provide more faithful models for the solution phase structures and energies. NVT ensemble dynamics at 300 K were performed with the Nosé-Hover thermostat. A gamma-centered 1x1x1 k-point mesh was used. A box size of 10x10x10 Å was first filled with solvent and the individual mediator(s) as an initial guess for the bulk solvation phase of the mediator. AIMD simulations were then carried out for five picoseconds to obtain the bulk solvation phase of the mediators. This equilibrated bulk solvated phase was then used as an initial guess for the solvent and mediator interacting with the Pd(111) surface with the surface O* adsorbates. In the condensed phase, a ~15 Å spacing was utilized in the vacuum region between the top and bottom layers to accommodate solvent and reacting molecules to mimic the density of the liquid phase solvent (~1 g cm^{-3} of water). This initial interfacial structure was equilibrated for another five picoseconds to allow the explicit solvent molecules to relax around the surface species and establish the lowest energy metal/mediator/adsorbates/solution phase structures. A vacuum of ~7 Å was then introduced at the top of the unit cell to avoid any spurious interactions of the bottom Pd surface with the solvent due to periodicity in the z-direction.

Periodic DFT calculations were carried out using a plane-wave energy cutoff of 400 eV. Corrections to the energy due to exchange and correlation effects were determined with the Perdew-Burke-Ernzerhof (PBE)[16] functional form of the generalized gradient approximation (GGA). PAW pseudopotentials[17] were used to describe the interactions between the valence and core electrons. Noncovalent long-range interactions in the system were modeled with the D3 dispersion corrections by Grimme.[18] Structural optimizations were carried out in two steps. The wavefunctions were first self-consistently optimized to within 10^{-4} eV using a 2 x 2 x 1 gamma-centered k-point mesh.[19] The atomic positions were iteratively optimized until the maximum force was less than 0.08 eV/Å. The wave functions were then optimized to within 10^{-6} eV using a 2 x 2 x 1 gamma-centered k-point mesh, and the maximum allowable force on each atom was less than 0.05 eV/Å. The nudged elastic band (NEB) method was initially used to locate the transition

states.[20] The NEB method represents the reaction path by interpolating images along the reaction coordinate, joining reactants to products. Each image was optimized until the tangential and normal forces were less than 0.08 eV/Å. Transition states were further refined using the dimer method.[21] The highest energy structure from the initial NEB path was used to provide an initial transition state structure for the dimer method, which was optimized until the forces converged to less than 0.05 eV/Å to find the true saddle point.

DOS and PDOS calculations were carried out with a 10 x 10 x 1 gamma-centered k-point mesh. The DOS plots have been generated with the Sumo toolkit.[22] Frequency calculations were carried out with a 5 x 5 x 1 gamma-centered k-point mesh. Isotope exchange from H to D was implemented in VASP by modifying the POMASS value for hydrogen to deuterium in the POTCAR file. All atoms, except the hydrogen atom being investigated for the kinetic isotope effect, were fixed during the frequency calculation. In line with the experiments, KIEs were calculated at 278 K for H vs. D and 298 K for H_2O vs. D_2O. Free energy corrections to the electronic energies for the reactant, transition state, and product structures were calculated using VASPKIT.[23]

References:

1. Adams, J. S.; Chemburkar, A.; Priyadarshini, P.; Ricciardulli, Y.; Lu, Y.; Maliekkal, V.; Sampath, A.; Winikoff, S.; M., K. A.; Neurock, M.; Flaherty, D. W., Solvent molecules form surface redox mediators in situ and cocatalyze O2 reduction on Pd. *Science* **2021,** *371* (6529), 626-632.
2. Ricciardulli, T.; Adams, J. S.; DeRidder, M.; van Bavel, A. P.; Karim, A. M.; Flaherty, D. W., H2O-Assisted O2 Reduction by H2 on Pt and PtAu Bimetallic Nanoparticles: Influences of Composition and Reactant Coverages on Kinetic Regimes, Rates, and Selectivities. *Journal of Catalysis* **2021,** *404*, 661-678.
3. Ricciardulli, T.; Gorthy, S.; Adams, J. S.; Thompson, C.; Karim, A. M.; Neurock, M.; Flaherty, D. W., Effect of Pd Coordination and Isolation on the Catalytic Reduction of O_2 to H_2O_2 over PdAu Bimetallic Nanoparticles. *Journal of the American Chemical Society* **2021,** *143*, 5445-5464.
4. Adams, J. S.; Kromer, M. L.; Rodriguez-Lopez, J.; Flaherty, D. W., Unifying Concepts in Electro- and Thermocatalysis toward Hydrogen Peroxide Production. *Journal of the American Chemical Society* **2021,** *143* (21), 7940-7957.
5. Wilson, N. M.; Flaherty, D. W., Mechanism for the Direct Synthesis of H2O2 on Pd Clusters: Heterolytic Reaction Pathways at the Liquid-Solid Interface. *Journal of the American Chemical Society* **2016,** *138* (2), 574-586.
6. Madon, R. J.; Boudart, M., Experimental Criterion for the Absence of Artifacts in the Measurements of Rates of Heterogenous Catalytic Reactions. *Industrial Engineering Chemical Fundamentals* **1982,** *21*, 438-447.
7. Scott, S. L., A Matter of Life(time) and Death. *ACS Catalysis* **2018,** *8* (9), 8597-8599.
8. Domingues, L.; Pinheiro, C. C.; Oliveira, N. M. C.; Vilelas, A.; Fernandes, J.; Ribeiro, F. R., Estimation of catalyst deactivation parameters of ethyl tert-butyl ether (ETBE) reactors based on industrial plant data. *22 European Symposium on Computer Aided Process Engineering* **2012,** *30*, 1002-1006.
9. Gaussian 16, Revision C.01, Frisch, M. J.; Trucks, G. W.; Schlegel, H. B.; Scuseria, G. E.; Robb, M. A.; Cheeseman, J. R.; Scalmani, G.; Barone, V.; Petersson, G. A.; Nakatsuji, H.; Li, X.; Caricato, M.; Marenich, A. V.; Bloino, J.; Janesko, B. G.; Gomperts, R.; Mennucci, B.; Hratchian, H. P.; Ortiz, J. V.; Izmaylov, A. F.; Sonnenberg, J. L.; Williams-Young, D.; Ding, F.; Lipparini, F.; Egidi, F.; Goings, J.; Peng, B.; Petrone, A.; Henderson, T.; Ranasinghe, D.; Zakrzewski, V. G.; Gao, J.; Rega, N.; Zheng, G.; Liang, W.; Hada, M.; Ehara, M.; Toyota, K.; Fukuda, R.; Hasegawa, J.; Ishida, M.; Nakajima, T.; Honda, Y.; Kitao, O.; Nakai, H.; Vreven, T.; Throssell, K.; Montgomery, J. A., Jr.; Peralta, J. E.; Ogliaro, F.; Bearpark, M. J.; Heyd, J. J.; Brothers, E. N.; Kudin, K. N.; Staroverov, V. N.; Keith, T. A.; Kobayashi, R.; Normand, J.; Raghavachari, K.; Rendell, A. P.; Burant, J. C.; Iyengar, S. S.; Tomasi, J.; Cossi, M.; Millam, J. M.; Klene, M.; Adamo, C.; Cammi, R.; Ochterski, J. W.; Martin, R. L.; Morokuma, K.; Farkas, O.; Foresman, J. B.; Fox, D. J. Gaussian, Inc., Wallingford CT. **2016**.
10. Zhao, Y.; Truhlar, D. G., The M06 suite of density functionals for main group thermochemistry, thermochemical kinetics, noncovalent interactions, excited states, and transition elements: Two new functionals and systematic testing of four M06-class functionals and 12 other function. *Theoretical Chemistry Accounts* **2008,** *120* (215-241).
11. Hariharan, P. C.; Pople, J. A., Accuracy of AHn equilibrium geometries by single determinant molecular orbital theory. *Molecular Physics* **1974,** *27*, 209-214.

12. Marenich, A. V.; Cramer, C. J.; Truhlar, D. G., Universal solvation model based on solute electron density and on a continuum model of the solvent defined by the bulk dielectric constant and atomic surface tensions. *Journal of Physical Chemistry B* **2009,** *113*, 6378–6396.
13. Kresse, G.; Hafner, J., Ab initio molecular-dynamics simulation of the liquid-metal-amorphous-semiconductor transition in germanium. *Phystical Review B* **1994,** *49* (20), 14251-14269.
14. Kresse, G.; Hafner, J., Ab initio molecular dynamics for liquid metals. *Physical Review B* **1993,** *47* (1), 558-561.
15. Demchenko, D. O.; Sacha, G. M.; Salmeron, M.; Wang, L. W., Interactions of oxygen and hydrogen on Pd(1 1 1) surface. *Surface Science* **2008,** *602*, 2552-2557.
16. Perdew, J. P.; Burke, K.; Ernzerhof, M., Generalized Gradient Approximation Made Simple. *Physical Review Letters* **1996,** *77*, 3865
17. Kresse, G., From ultrasoft pseudopotentials to the projector augmented-wave method. *Physical Review B* **1999,** *59*, 1758–1775
18. Stefan, G.; Antony, J.; Ehrlich, S.; Krieg, H., A consistent and accurate ab initio parametrization of density functional dispersion correction (DFT-D) for the 94 elements H-Pu. *The Journal of Chemical Physics* **2010,** *132*, 154104
19. Monkhorst, H. J.; Pack, J. D., Special points for brillouin-zone integrations. *Physical Review B* **1976,** *13* (12), 5188-5192.
20. Henkelman, G.; Uberuaga, B. P.; Jonsson, H., A climbing image nudged elastic band method for finding saddle points and minimum energy paths. *Journal of Chemical Physics* **2000,** *113* (22), 9901-9904.
21. Henkelman, G.; Jonsson, H., A dimer method for finding saddle points on high dimensional potential surfaces using only first derivatives. *Journal of Chemical Physics* **1999,** *111* (15), 7010-7022.
22. M Ganose, A.; J Jackson, A.; O Scanlon D., sumo: Command-line tools for plotting and analysis of periodic ab initio calculations. *J. Open Source Softw* **2018,** 3, 717.
23. Wang, V.; Xu, N.; Liu, J.C.; Tang, G.; Geng, W.T.; VASPKIT: A User-Friendly Interface Facilitating High-Throughput Computing and Analysis Using VASP Code, *Computer Physics Communications* **2021,** 267, 108033.
